# Interplay between the Oxygen Reduction Reaction and Atom Transfer Radical Polymerization with Molecular Cu-Based Catalysts in Water

**DOI:** 10.1021/acscatal.5c04928

**Published:** 2025-08-07

**Authors:** Phebe H. van Langevelde, Katarina Ležaić, Jorge F. J. Coelho, Dennis G. H. Hetterscheid, Francesco De Bon

**Affiliations:** † Leiden Institute of Chemistry, Leiden University, 2300 RA Leiden, The Netherlands; ‡ CEMMPRE, ARISE, Department of Chemical Engineering, 37829 University of Coimbra, Rua Sílvio Lima, Pólo II, 3030-790 Coimbra, Portugal; § IPN, Instituto Pedro Nunes, Associação para a Inovação e Desenvolvimento em Ciência e Tecnologia, Rua Pedro Nunes, 3030-199 Coimbra, Portugal

**Keywords:** oxygen reduction reaction, atom transfer radical polymerization, electrocatalysis, copper complexes, hydrogen peroxide, electrochemistry

## Abstract

Determining the relationships between the catalyst structures of Cu-based molecular complexes and their performances in the oxygen reduction reaction (ORR), hydrogen peroxide reduction reaction (HPRR), and electrochemically mediated Atom Transfer Radical Polymerization (*e*ATRP) is crucial for advancing radical polymerization in aerobic environments. Hence, Cu/tris­(2-pyridylmethyl) amine (TMPA) was compared with Cu catalysts with TMPA ligands containing electron-donating *para*-substituents for aqueous ORR/HPRR catalysis and ATRP. *Para*-substitution decreased the ORR and HPRR activities, thereby reducing the O_2_ consumption compared to that of Cu/TMPA. *Para*-substituted TMPA catalysts were suitable for acrylate polymerization, with *M*_w_/*M*_n_ < 1.3, whereas the reaction rates of methacrylates were too high, leading to polymethacrylates with high *M*_w_/*M*_n_. Our results show that ORR/HPRR and ATRP are competitive, intertwined processes in aerobic water, with reactivities diverging upon the introduction of electron-donating *para*-substituents. These findings will facilitate future efforts to optimize Cu-based molecular catalysts by tuning their ligand structures to improve the performance of both ATRP and ORR/HPRR.

## Graphical abstract

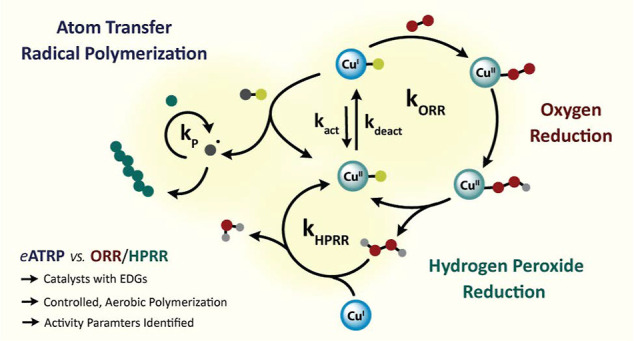

## 1 Introduction

The combination of multiple catalytic processes in a single reaction environment can lead to competitive catalysis, an interplay between individual mechanisms, and possibly mutual benefits.^1^ An example is the competition between Cu-mediated atom transfer radical polymerization (ATRP)^2–4^ and the oxygen reduction reaction (ORR),^5,6^ which enables aerobic polymerization in water. ATRP is a key technique in reversible deactivation radical polymerization (RDRP) that provides precise control over the polymer chain length and end-group functionality by maintaining a dynamic equilibrium between the active and dormant chains. This balance allows the growth of well-defined macromolecules with controlled molecular weights and low polydispersity (*D̵* = *M*_w_/*M*_n_, the ratio between the weight-average and number-average molecular weights, typically <1.5). Thus, ATRP is one of the most widely used RDRPs for synthesizing polymers with specific properties and architecture. For example, ATRP has enabled the creation of stimuli responsive and biocompatible polymers, facilitating various applications in (bio)­chemistry and material science.^7–9^

Although initially developed with iron catalysts,^10^ Cu-based complexes with multidentate *N*-coordinating ligands^11^ are the most commonly used ATRP catalysts, whereas alkyl halides (R-X, X = Br, Cl) serve as initiators.^12^ [Cu^I^L]^+^ activators and [X–Cu^II^L]^+^ deactivators (X = Br^–^ or Cl^–^) dynamically control chain activation and deactivation, which is essential for controlled polymerization reactions. Chain initiation involves the activation of R-X by [Cu^I^L]^+^ (*k*_act_) to generate radicals that propagate by adding a few monomer units to the growing chain. Chain deactivation occurs via a rapid reaction between [X–Cu^II^L]^+^ (*k*_deact_) and the propagating radical, ensuring controlled growth and minimal side reactions. The ratio of the two constants defines the equilibrium constant as *K*_ATRP_ = *k*_act_/*k*_deact_.

The reduced and active [Cu^I^L]^+^ species can be obtained using several methodologies including photochemical (photoATRP),^13–19^ thermal (ICAR ATRP),^20–23^ addition of a reducing agent (ARGET ATRP),^24–30^ and electrochemical (*e*ATRP) methods. *e*ATRP is an electrochemical variant that uses an applied potential (*E*_app_) or current (*I*_app_) to drive polymerization by generating [Cu^I^L]^+^ ions on the electrode surface. *e*ATRP can be conducted in both divided^31–34^ and undivided electrochemical cells,^35–40^ homogeneous^41^ and heterogeneous forms,^42–44^ under pressure,^45^ from surfaces,^46–49^ with alternating or pulsed currents,^50,51^ and up to a 15 L scale.^52,53^ The development of aqueous ATRP is crucial,^54–56^ as water is the most benign and economical solvent for polymerizations, either as a homogeneous solution or as dispersant in an emulsion.^57^ To control ATRP in water, the high activity of [Cu^I^L]^+^ towards alkyl halides needs to be moderated. This can be easily achieved by applying a voltage (*E*_app_) equal to or higher than the standard redox potential (*E*_1/2_) of the catalyst; thus, only a fraction of [Cu^II^L]^+^ is electroreduced at the electrode surface, according to the Nernst equation.

Inspired by the rich oxygen chemistry of various copper enzymes, molecular Cu catalysts^58^ have been investigated as catalysts for the oxygen reduction reaction (ORR). Several of these Cu complexes have been reported to be suitable catalysts for the ORR, and Cu-based catalysts with *N*-coordinating tetradentate tripodal ligands incorporating structural modifications to enhance the catalytic activity or selectivity have been developed.^59–61^ These modifications include coordinating multiple copper sites,^62–65^ altering the methyl spacers,^66,67^ and adding functional groups to the ligand.^59,67–69^ Among these catalysts, Cu/TMPA (TMPA = tris­(2-pyridylmethyl)­amine)^70^ is notable for both its high TOF_max_^ORR^ > 10^6^ s^–1^ and its ATRP activity.^54,56,71^ Cu/TMPA facilitates the ORR in a stepwise fashion, producing H_2_O_2_ as an intermediate in the initial 2e^–^ reduction (eq 1). Subsequently, H_2_O_2_ is further reduced to H_2_O via the 2e^–^ hydrogen peroxide reduction reaction (HPRR) (eq 2).

1 $$ORR:O_{2}+{2H}^{+}+{2e}^{-}\to H_{2}O_{2}$$

2 $$HPRR:H_{2}O_{2}+{2H}^{+}+{2e}^{-}\to {2H}_{2}O$$

Although Cu catalysts for ATRP and ORR were developed separately, it has been showed that both processes can be driven simultaneously thereby allowing radical polymerization to take place in the presence of oxygen,^13,14,52,72–76^ which is rapidly removed from the reaction mixture through ORR catalysis (Scheme 1).^14,74,77^

**Scheme 1:**
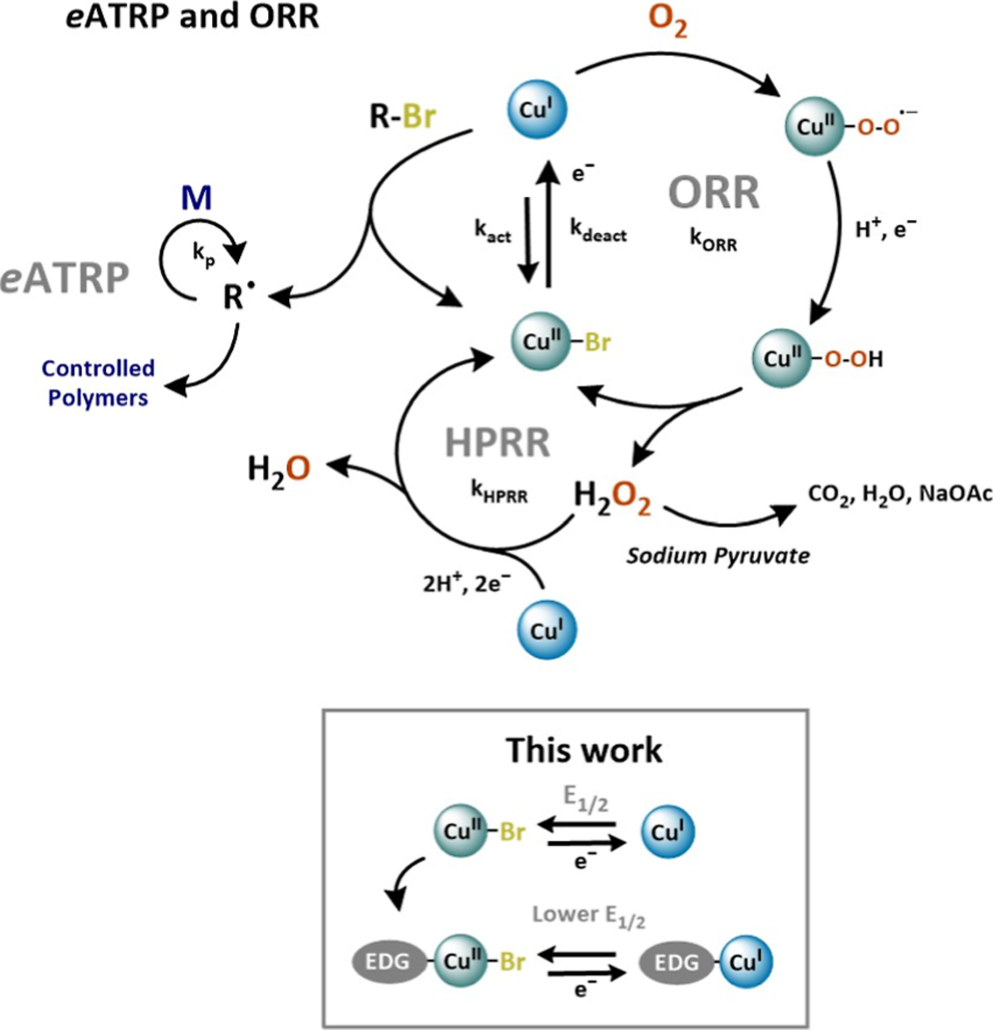
Proposed Mechanism of *e*ATRP With an Embedded O_2_ Scavenging Cycle for Aerobic Radical Polymerization^a^

a M is the monomer, R-Br is the alkyl halide ATRP initiator, and *k*_act_, *k*_deact_, and *k*_p_ are the activation, deactivation, and propagation rate constants of ATRP, respectively. In the bottom panel, EDG indicates the electron-donating group attached to the TMPA ligand, which is omitted from the scheme for clarity. All the Cu species in this scheme have an overall charge of +1.

ATRP in water enables greener polymer synthesis because the use of organic solvents at scale is discouraged due to their cost, toxicity, and environmental impact.^78^ Emulsion polymerization in water is indeed one of the dominant processes in industry for producing commodity polymers, and ATRP has been shown to operate in untreated natural waters, including river, rain, and seawater, highlighting its robustness.^79^

In free-radical polymerization, O_2_ can function as an inhibitor and even as a radical initiator in certain situations. Nevertheless, the most prevalent effect of O_2_ during free radical polymerization is inhibition; it reacts more quickly than the monomers in the propagation reaction because of its partial radical nature, which stems from its triplet ground state. When O_2_ is incorporated into a chain, it forms a relatively stable radical, called a polyperoxide. It reacts slowly with monomers to create copolymers (nonradical polyperoxides) and accumulates, leading to termination. This results in the deceleration of the reaction and net consumption of the initiator. These polyperoxides also remained thermally stable up to 100–150 °C. The presence of O_2_ impurities during free-radical polymerization generally causes a slowdown, sometimes temporary, in the polymerization rate, resulting in products with lower molecular weights and higher polydispersity.

Although ATRP techniques are adaptable and effective under various reaction conditions, they are sensitive to O_2_, which acts not only as an efficient radical scavenger but also by oxidizing (and thus deactivating) [Cu^I^TMPA]^+^ species. To prevent the problems caused by O_2_, it is essential to completely remove it before starting polymerization by introducing argon or nitrogen into the reactor and using vacuum pumps, selective absorption pumps, or gloveboxes. However, these methods can be costly and time-consuming, and may complicate the reaction setup, sometimes making them incompatible with biological substrates (proteins or cells) and hindering the industrial application of ATRP.^77,80–82^ As noted by industrial sources such as PPG, a major patent holder in ATRP technologies, one of the main barriers to the broader adoption of ATRP in the industry is its poor cost-to-performance ratio owing to O_2_ interference at large volumes.^83,84^ Despite its maturity, the oxygen sensitivity of ATRP remains a significant limitation, particularly for industrial-scale applications, where rigorous deoxygenation is either impractical or cost-prohibitive. Thus, large-scale reactions under strictly deoxygenated conditions remain poorly feasible. Thus, the development of catalysts and polymerization systems that function in the presence of oxygen is not only scientifically interesting but also critical for industrial viability. Many recent studies have demonstrated the growing importance of conducting ATRP in the presence of molecular O_2_, especially in aqueous systems, using ARGET, photoATRP, and electrochemically mediated approaches.^13,14,16,18,52,73,75,77,80,85–88^ In the presence of O_2_, aqueous ATRP and molecular ORR/HPRR (not merely two parasitic side reactions) become competitive and interconnected. When Cu-mediated aqueous ATRP is conducted under aerobic conditions, the ORR serves as the operative deoxygenation mechanism. The Cu catalyst was engaged in three equilibria (Scheme 1). [Cu^I^L]^+^ primarily participates in the fastest process, which is dictated by its highest rate constant (*k*_ORR_, *k*_HPRR_, or *k*_act_). In water, the activation rate constant (*k*_act_) of [Cu^I^TMPA]^+^ for the hydrophilic ATRP initiator 2-hydroxyethyl 2-bromoisobutyrate (HEBiB)^54,56^ is >5.6 × 10^6^ M^–1^s^–1^ and the value of TOF_max_^ORR^ is >10^6^ s^–1^, which, in the case of water saturated with oxygen, corresponds to an observed *k*_ORR_ > 10^9^ M^–1^ s^–1^, assuming a first order dependence on the oxygen concentration.^89^ Conversely, HPRR is generally slower than ORR,^66^ which may lead to the accumulation of H_2_O_2_ and other hydroperoxide intermediates in solution, posing challenges for ATRP as H_2_O_2_ promotes side reactions. Thus, H_2_O_2_ scavengers, such as the inexpensive and biocomcompatible sodium pyruvate (SP), are typically added.^52,73,88^ Previous works have shown that [Cu^I^Me_6_TREN]^+^ (Me_6_TREN = tris­[2-(dimethylamino)­ethyl]­amine) predominantly engages in ORR rather than *e*ATRP under continuous O_2_ replenishment.^52^ *e*ATRP commenced only after sealing the reactor to prevent O_2_ diffusion and after the consumption of most O_2_ in the polymerization mixture.^52^ Identifying the optimal catalyst to balance the fast ORR/HPRR and ATRP is not a trivial task, and many aspects remain unclear. For example, *K*_ATRP_ is highly monomer-dependent, which implies that reconfiguration of the balance between the ORR and ATRP rates is necessary. This may further complicate the control of the relative ORR and ATRP rates. The standard redox potential (*E*_1/2_) of the catalyst is a key parameter for both ORR and ATRP; thus, the electron-donating properties of the TMPA ligand are expected to significantly influence the ATRP and ORR activities. The addition of electron-donating groups (EDGs) to the TMPA ligand is expected to lower the *E*_1/2_ of Cu catalysts,^90–94^ thereby significantly increasing *k*_act_ and *K*_ATRP_. Previous works have shown that the introduction of EDGs onto the TMPA scaffold at the *para* position lowers *E*_1/2_ and increases *K*_ATRP_ by up to 4–6 orders of magnitude.^90,91,95^ Similarly, a more negative *E*_1/2_ results in faster activation of O_2_ by [Cu^I^L]^+^, thereby enhancing the ORR rate. Indeed, copper catalysts with tripodal tetradentate ligands achieve higher ORR rates with a more negative *E*_1/2_.^66^ However, the electronic effects on simultaneous ORR/HPRR and ATRP have not been explored thus far. Furthermore, the ORR activity and selectivity of Cu catalysts in electrolytes commonly employed in aqueous ATRP, such as NaBr, have not been previously investigated.

This work explores the parameters that are important for achieving optimal *e*ATRP in the presence of simultaneous ORR/HPRR in aqueous media. We systematically investigated the electrochemical ORR activity and H_2_O_2_ selectivity were affected by the introduction of introducing EDGs onto the TMPA scaffold, evaluated the ORR activity and H_2_O_2_ selectivity under conditions relevant to *e*ATRP, and subsequently applied these catalysts to aerobic *e*ATRP. Ultimately, our findings can be used to identify the design criteria for catalysts for simultaneous ORR/HPRR and *e*ATRP.

## 2 Results and Discussion

### 2.1 Synthesis of *Para*-substituted TMPA Ligands

Previous studies have shown that pyrrolidine is an effective *para*-EDG for TMPA ligands in ATRP.^50,90,92^ However, substitution causes both the ligand and the resulting catalyst to become highly hydrophobic, restricting their application in aqueous *e*ATRP. Therefore, we used *N*-methylaminoethanol as an EDG to maintain hydrophilicity while adjusting the electronic properties of TMPA. First, 4-chloropyridine TMPA precursors^90^ (TMPA-(Cl)_*n*_, *n* = 1–3) were synthesized (Scheme 2). The resulting precursors were reacted with *N*-methylaminoethanol via nucleophilic aromatic substitution to obtain *para*-substituted ligands (4-(aminomethyl)­ethanolpyridyl)-bis-*N*-pyridylmethylamine (TMPA-(OH)_1_), bis­(4-(aminomethyl)­ethanolpyridyl)-*N*-pyridylmethylamine (TMPA-(OH)_2_), and tris­(4-(aminomethyl)­ethanolpyridylmethyl)­amine (tmpa-(OH)_3_) (TMPA-(OH)_3_) ligands (Scheme 2). For the ORR and HPRR experiments, the ligands were reacted with Cu­(OTf)_2_ in acetonitrile to form complexes [Cu^II^(TMPA-(OH)_1_)]^2+^ (Cu/TMPA-(OH)_1_), [Cu^II^(TMPA-(OH)_2_)]^2+^ (Cu/TMPA-(OH)_2_), and [Cu^II^(TMPA-(OH)_3_)]^2+^ (Cu/TMPA-(OH)_3_). The ligands were characterized by NMR spectroscopy, and the Cu complexes were further analyzed by elemental analysis and electrochemical measurements (Supporting Information). The ligands were used to generate their respective Cu complexes, Cu/TMPA-(OH)_*n*_, by reacting with Cu­(OTf)_2_.

**Scheme 2:**
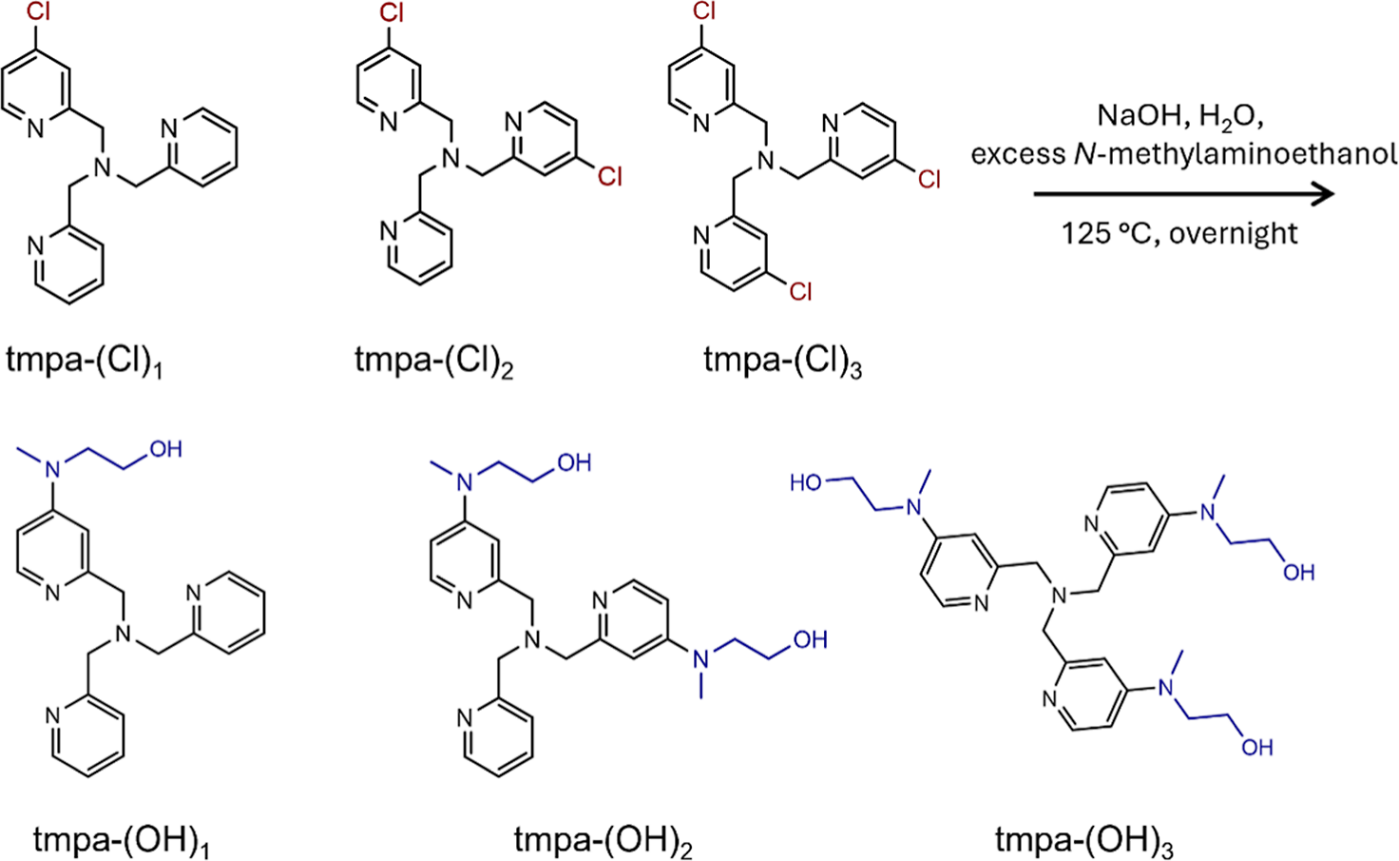
Synthesis of *Para*-substituted Tris­(2-pyridylmethyl)­amine In the Ligand Name, TMPA-(OH)_*n*_, Where *n* = 1, 2, and 3, Indicates the Number of Pyridine Rings Containing a Substituent (Top)

### 2.2 Redox Behavior in 0.1 M PB or 0.1 M NaBr

Cyclic voltammetry (CV) measurements of Cu/TMPA-(OH)_1_, Cu/TMPA-(OH)_2_, and Cu/TMPA-(OH)_3_ were performed in 0.1 M phosphate buffer (PB) at pH 7 and in 0.1 M NaBr under an Ar atmosphere and compared with those of Cu/TMPA. PB is commonly used to study Cu-based molecular ORR catalysts^62,66,70^ while NaBr is typically added in aqueous ATRP experiments to produce the deactivator [Br–Cu^II^L]^+^.^54,56^

All recorded redox couples in PB were observed at nearly the same potential (Figure 1). The analysis of the half-wave potential (*E*_1/2_) showed minimal mutual shifts, with no clear relationship between the number of EDGs and *E*_1/2_ (Table 1 for selected properties and Table S1 for all the properties). The electronic effect of the *para*-substituted group on the Cu center became more evident in 0.1 M NaBr solution. The *E*_1/2_ values of the catalysts shifted to at least 30 mV more negative than those of Cu/TMPA (Table 1 and Figure 1), correlating with the number of EDGs, with the largest shift observed after the addition of the first EDG to the TMPA scaffold. This difference between PB and NaBr is attributed to the formation of a [Br–Cu^II^L]^+^ species in NaBr, where the *E*_1/2_ values are affected more by *para*-substitution on the TMPA scaffold. In PB, an additional oxidation peak appeared in the CV signals of Cu/TMPA-(OH)_2_ and Cu/TMPA-(OH)_3_ (Figure 1a), which is likely due to the stripping of electrodeposited Cu^0^ from the GC surface and indicates the disproportionation of the electrogenerated [Cu^I^L]^+^. The increased *para*-substitution of TMPA appeared to facilitate disproportionation and increase the peak-to-peak separation (Δ*E*_p_) (Table S1). These PB results differed from the CVs in NaBr, where all catalysts showed similar Δ*E*_p_ values and no Cu^0^ stripping, likely because the bromide anions stabilized the [Cu^I^L]^+^ oxidation state. Fifty consecutive scans of Cu/TMPA-(OH)_3_ in PB indicated increased Cu^0^ deposition on the GC surface and depletion of the complex near the electrode. The same 50 scans in NaBr demonstrated the high reproducibility of the redox couple, indicating better stability of the Cu species under *e*ATRP conditions (see Supporting Information Section S6). The cathodic peak current (*I*_pc_) exhibited a linear dependence on the square root of the scan rate (see Supporting Information, Section S5), indicating a diffusion-controlled process and the formation of homogeneous Cu complexes in the electrolyte. Laviron plots, which depict the potential of the redox peaks as a function of the logarithm of the scan rate, also suggest fast electron transfer with 0.1 M NaBr compared to PB on the CV time scale (Figures S1–S3e,f and S4). The diffusion coefficients of all catalysts in both the phosphate buffer and 0.1 M NaBr were calculated from the slopes of the Randles–Sevcik plots (Table 1). These coefficients are slightly higher in NaBr, consistent with earlier trends reported for Cu catalysts.^56,96,97^

**Figure 1:**
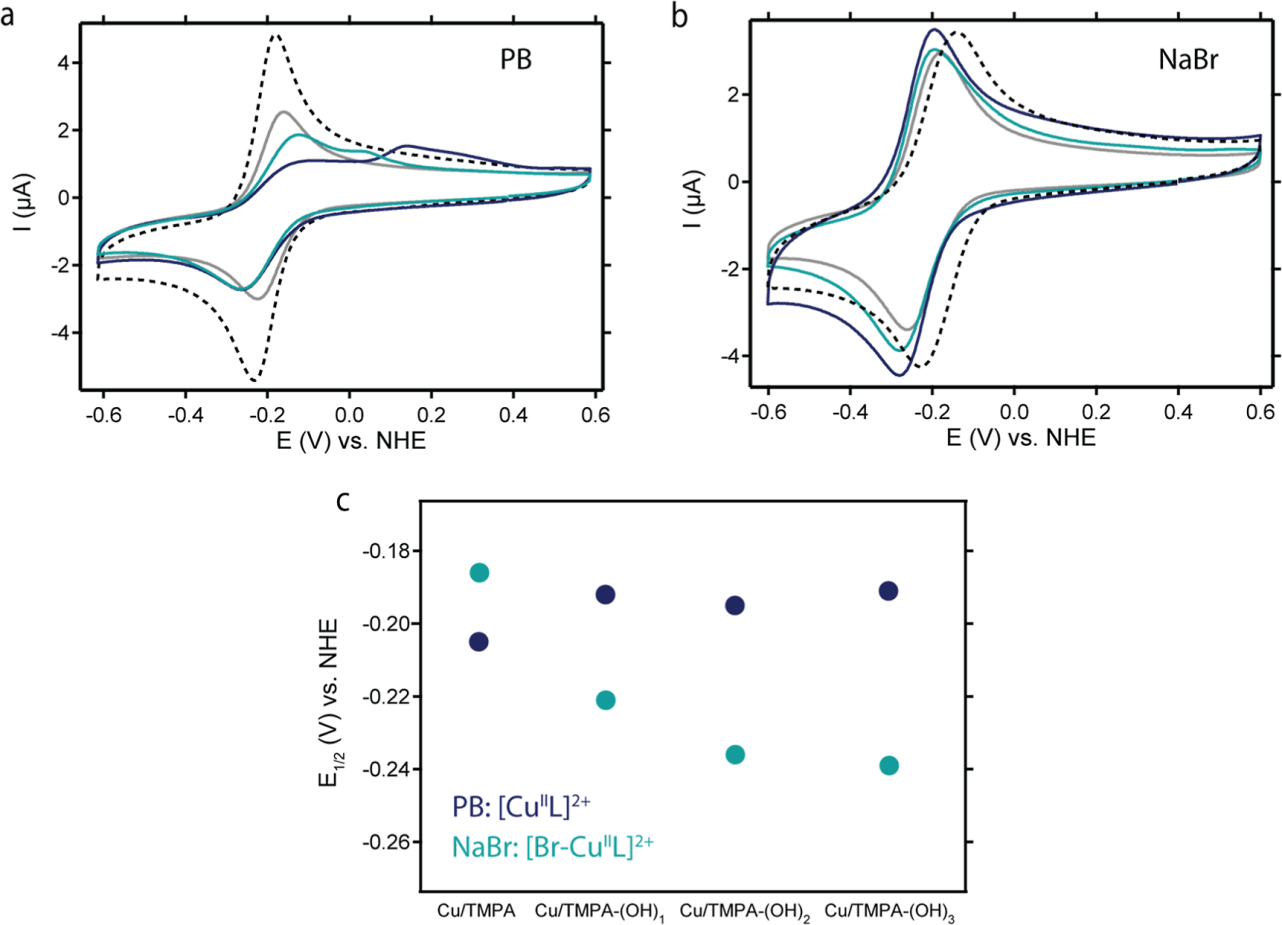
CV measurements of Cu/TMPA-(OH)_1_ (gray), Cu/TMPA-(OH)_2_ (light blue), and Cu/TMPA-(OH)_3_ (dark blue) compared to Cu/TMPA (black dotted line) recorded in 0.1 M PB pH 7 (a) or 0.1 M NaBr pH 7 (b). Evolution of *E*_1/2_ of [Cu^II^L]^2+^ in PB (dark blue) and [Br–Cu^II^L]^+^ in 0.1 M NaBr (light blue). Conditions: C_Cu_ = 0.3 mM, 1 atm Ar, all CV were recorded on a GC disk electrode at 0.1 V/s.

**Table 1:** Selected Redox Properties of All the Catalysts Were Obtained from the CV Measurements

catalyst	electrolyte	*E*_1/2_ (mV) vs NHE	*D* (cm^2^ s^–1^)^a^
Cu/TMPA	0.1 M PB^a^	–205	4.90 × 10^–6^ ^d^
	0.1 M NaBr^a^	–186	n.d.
	0.1 M SP + 0.01 M PB^b^	–157	n.d.
	0.1 M SP + 4 mM NaBr + 10 vol % OEOA_480_ ^b^	–15 (−101)^e^	n.d.
	0.1 M SP + 0.1 M NaBr + 10 vol % OEOMA_500_ ^b^	–56	n.d.
Cu/TMPA-(OH)_1_	PB^a^	–192	4.89 × 10^–6^
	NaBr^a^	–221	4.94 × 10^–6^
	0.1 M SP + 0.01 M PB^c^	–177	n.d.
	0.1 M SP + 4 mM NaBr + 10 vol % OEOA_480_ ^c^	–29	n.d
	0.1 M SP + 0.1 M NaBr + 10 vol % OEOMA_500_ ^c^	–93	n.d.
Cu/TMPA-(OH)2	PB^a^	–195	5.47 × 10^–6^
	NaBr^a^	–236	6.30 × 10^–6^
	0.1 M SP + 0.01 M PB^c^	–193	n.d.
	0.1 M SP + 4 mM NaBr + 10 vol % OEOA_480_ ^c^	–44	n.d.
	0.1 M SP + 0.1 M NaBr + 10 vol % OEOMA_500_ ^c^	–126	n.d.
Cu/TMPA-(OH)_3_	PB^a^	–191	5.18 × 10^–6^ ^b^
	NaBr^a^	–239	6.62 × 10^–6^
	0.1 M SP + 0.01 M PB^c^	–250 (−377)^e^	n.d.
	0.1 M SP + 4 mM NaBr + 10 vol % OEOA_480_ ^c^	(−224)^e^	n.d.

a CVs curves were recorded on a GC disk electrode at a scan rate of 100 mV/s at room temperature, and the diffusion coefficients were calculated from the reduction peaks.

b Calculated with high uncertainty owing to the irreproducibility of the redox couple.

c CVs were recorded on a GC disk electrode at a scan rate of 200 mV/s at 35 °C using an SCE reference electrode in the presence of 0.01 M PB. The potential values were then converted to NHE using *E*_NHE_ = *E*_SCE_ + 0.233 V.

d From ref 89.

e *E*_pc_. n.d. = not determined. PB, phosphate buffer; SP, sodium pyruvate.

### 2.3 Redox Behavior in the Presence of OEOMA_500_ and OEOA_480_

After investigating *E*_1/2_ and the stability of the catalysts in PB and NaBr, further electrochemical characterization of the Cu complexes was performed in the presence of sodium pyruvate and the hydrophilic monomers poly­(ethylene glycol) methyl ether methacrylate (OEOMA_500_) and poly­(ethylene glycol) methyl ether acrylate (OEOA_480_) (Scheme 3 and Figures 2 and 3), which are interesting monomers to produce biocompatible bottlebrush polymers by *e*ATRP.^41^

**Scheme 3:**
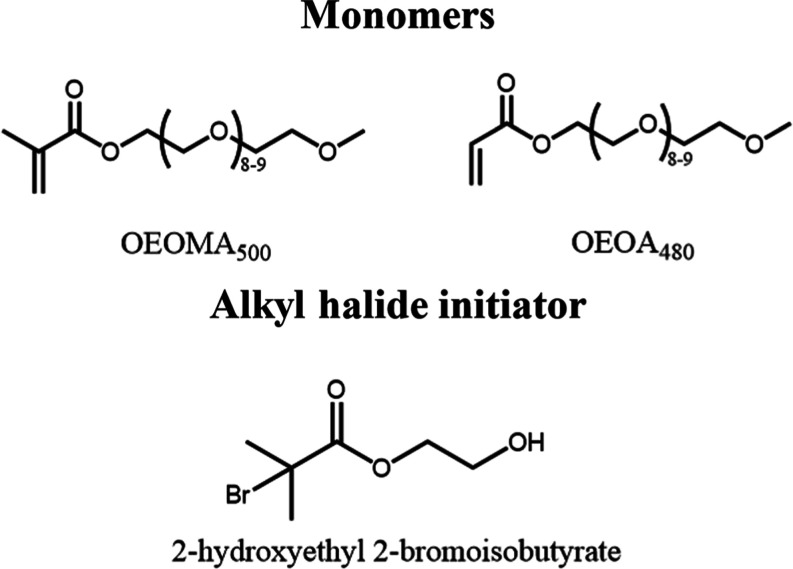
Chemical Structures of the Monomers and Alkyl Halide Initiators Used during *e*ATRP in This Work

**Figure 2:**
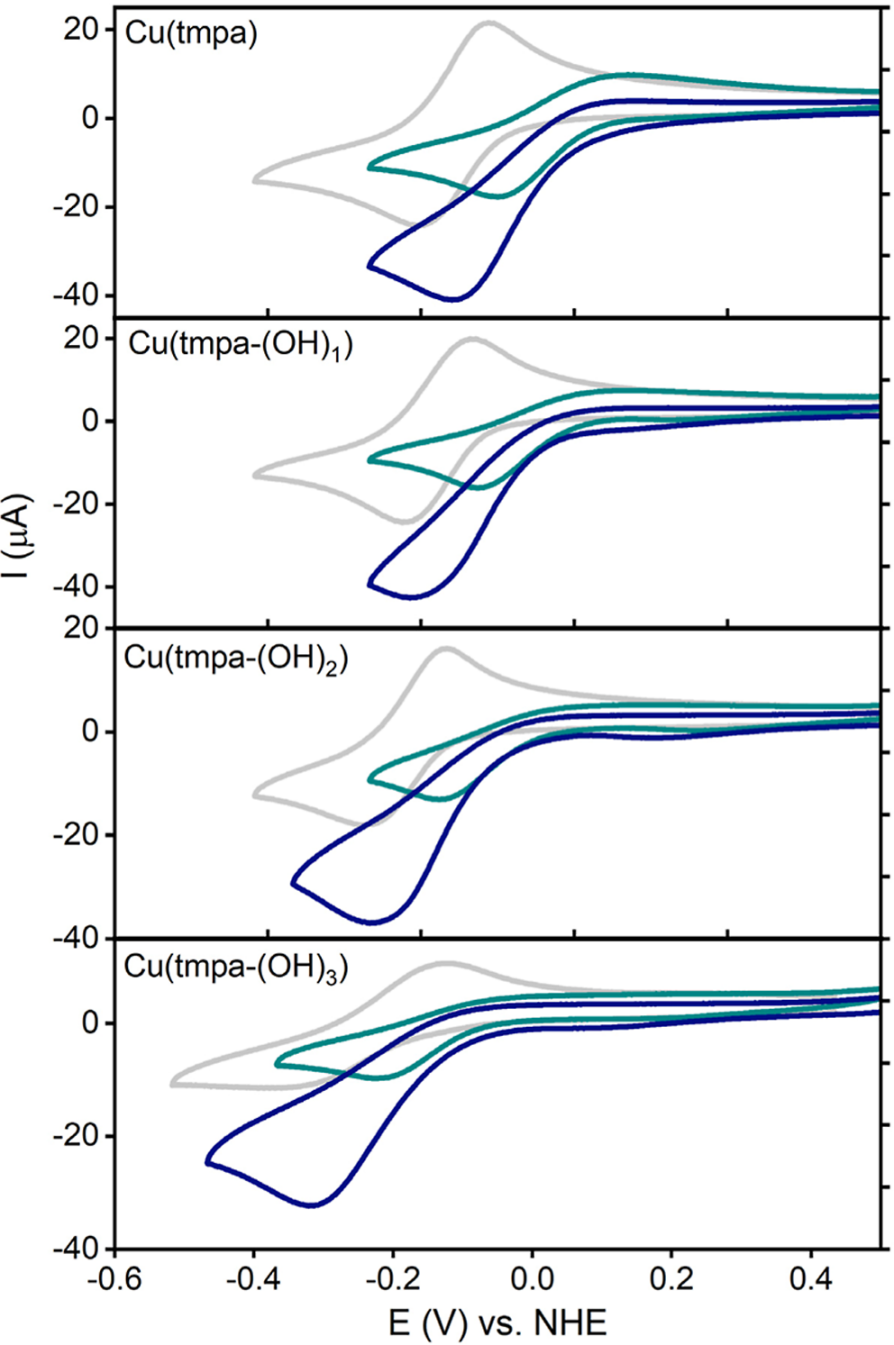
Cyclic voltammetry measurements of all copper complexes recorded before polymerization (gray), in the presence of 10 vol % OEOA_480_ in 0.1 M SP + 0.01 M PB, and 4 mM NaBr in H_2_O (light blue), and upon addition of 2 mM HEBiB (dark blue). Conditions: N_2_ atmosphere, 1 mM [Cu^II^L]^2+^, υ = 0.2 V/s, *T* = 35 °C. Ligands, in order from top to bottom: TMPA, TMPA-(OH)_1_, TMPA-(OH)_2_, and TMPA-(OH)_3_.

**Figure 3:**
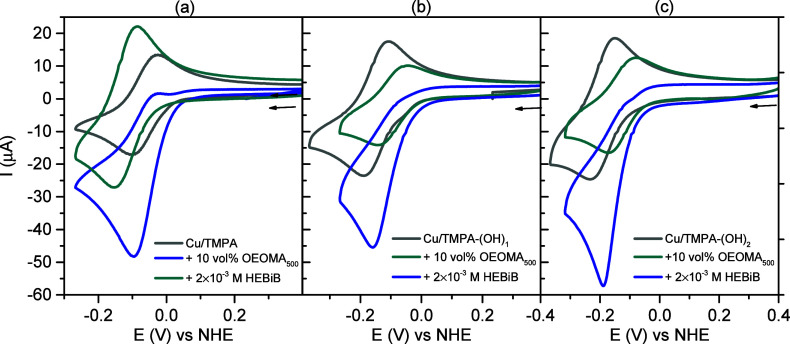
(a) Cyclic voltammetry of [Cu^II^TMPA]^2+^, [Cu^II^TMPA-(OH)_1_]^2+^, and [Cu^II^TMPA-(OH)_2_]^2+^ recorded before polymerization (gray), in the presence of 10 vol % OEOMA_500_ in 0.1 M SP + 0.01 M PB and 0.1 M NaBr (light blue), and upon the addition of 2 mM HEBiB (dark blue). Conditions: N_2_ atmosphere, 1 mM [Cu^II^L]^2+^, υ = 0.2 V/s, *T* = 35 °C.

Cu/TMPA catalyzes the HPRR at a lower rate than the reduction of oxygen.^98^ Hence, H_2_O_2_ and other hydroperoxide intermediates accumulate in solution, posing challenges for ATRP due to side reactions. Therefore, the inexpensive and biocompatible H_2_O_2_ scavenger sodium pyruvate (SP) was used as the *e*ATRP electrolyte.^52,73,88^ All the catalysts showed reversible redox couples in the presence of 0.1 M SP. The addition of 10 vol. % OEOA_480_ decreased the anodic current and reduced the cyclic voltammetry signal (Figure 2). This behavior was previously reported in water^41^ and can be attributed to lower electron transfer rates in the presence of OEOA_480_.^41^ Upon the addition of HEBiB, the CV shape changed to a catalytic wave, and the anodic current vanished owing to the initiation of ATRP catalysis.

Using 10 vol % of the hydrophilic methacrylate OEOMA_500_ (Figure 3), we observed a similar behavior; however, unlike OEOA_480_, the CV of the Cu catalysts remained reversible. The addition of HEBiB resulted in the onset of ATRP catalysis,^97^ consistent with previous reports.^56^ *e*ATRP of OEOMA_500_ was not attempted with [Cu^II^TMPA-(OH)_3_]^2+^; hence, cyclic voltammograms were not recorded.

The electrochemical characteristics of all the catalysts obtained from the cyclic voltammetry experiments are listed in Table 1, Figure 3, and Table S1. There is a good correlation between the redox potential of the catalyst and *K*_ATRP_, and between the number of substituents on the pyridine ring and the redox potential of the catalyst.^12,90,99,100^ However, as the number of substituents increases, Δ*E*_p_ (*E*_pc_–*E*_pa_) also increases, indicating a loss of reversibility. Notably, the cathodic peak potential (*E*_pc_) of [Br–Cu^II^L]^+^, measured in the absence of monomers or initiators, decreased by 43 mV per substituent, whereas the *E*_pc_ of [Cl–Cu^II^L]^+^ under identical conditions decreased by 38 mV per substituent. Such a linear Bell-Evans–Polanyi relationship has also been observed for other Cu complexes using *para*-substituted TMPA and bipyridines.^90,93,94^ Monomer addition reduces the polarity of the polymerization medium, which shifts the *E*_1/2_ in the following order: *E*_1/2_^H_2_O^ < *E*_1/2_^OEOMA_500_^ < *E*_1/2_^OEOA_480_^. The *E*_1/2_ of [Cu^II^L]^+^ was shifted up to 48 mV more positive compared to *E*_1/2_^H_2_O^ in the presence of sodium pyruvate, which can coordinate to Cu complexes, and the higher temperature (35 °C vs 25 °C) used in polymerization and the presence of the monomer (Table 1).

### 2.4 Catalytic Activity for the Oxygen Reduction Reaction (ORR) and Hydrogen Peroxide Reduction Reaction (HPRR)

We assessed the ORR activity of *para*-substituted Cu complexes in 0.1 M phosphate buffer in the presence of 0.1 M NaBr and compared them with Cu/TMPA. All the catalysts exhibited a peak-shaped catalytic wave in the presence of O_2_ (Figure 4). The peak shape from this wave results from mass diffusion limitations as O_2_ rapidly depletes in the diffusion layer. For all the catalysts, the first CV scan in 0.1 M NaBr showed a more positive onset potential (*E*_onset_) than the subsequent scans, which was attributed to proton depletion and a local pH shift (Figure S5).

**Figure 4:**
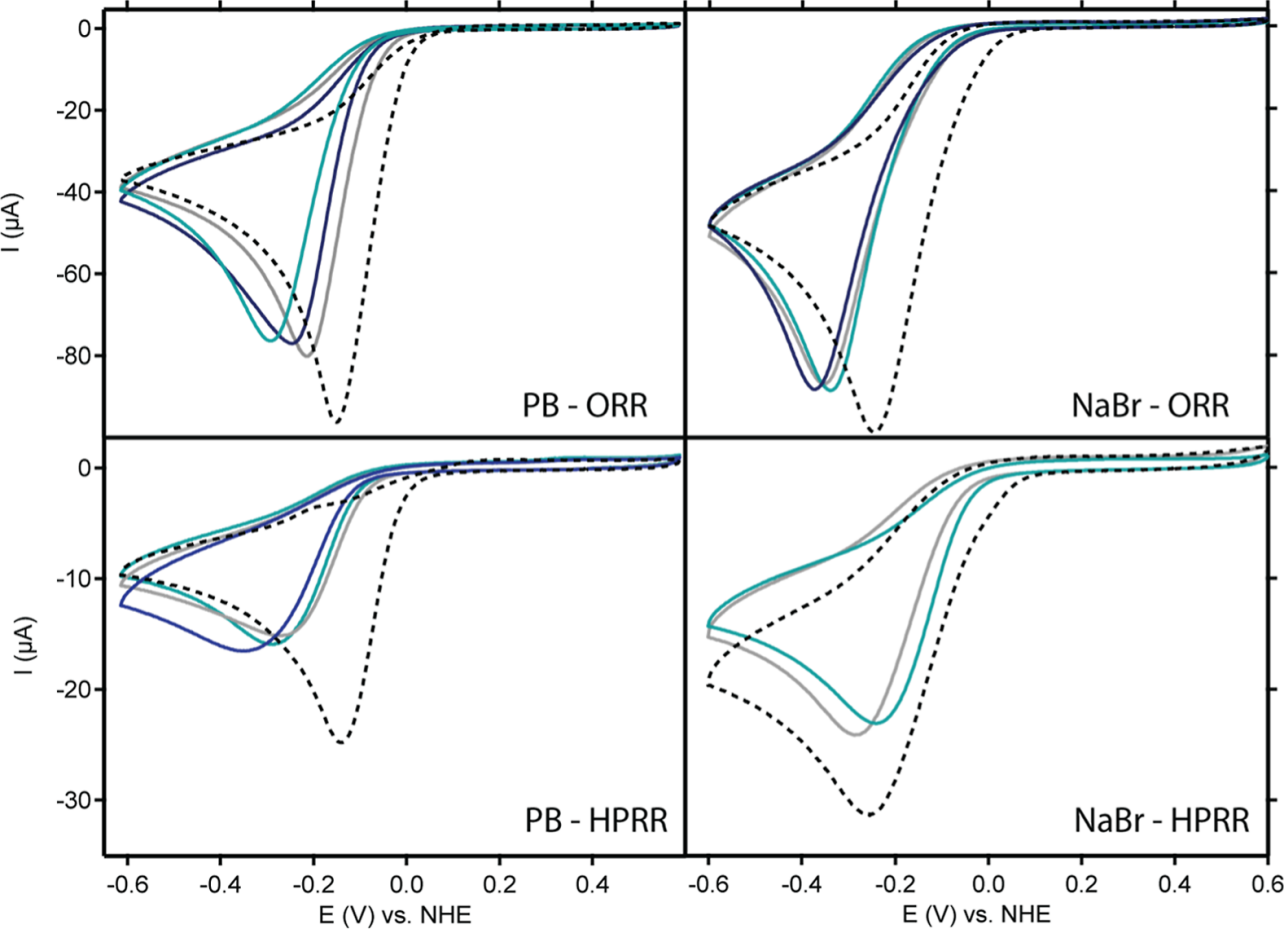
CV measurements of the ORR activity of Cu/TMPA-(OH)_1_ (gray), Cu/TMPA-(OH)_2_ (light blue), and Cu/TMPA-(OH)_3_ (dark blue) compared to Cu/TMPA (black dotted line) recorded in 0.1 M PB, pH 7, or 0.1 M NaBr. Conditions: *C*_Cu_ = 0.3 mM, *C*_H_2_O_2__ = 1.1 mM, 1 atm O_2_, υ = 0.1 V/s.

We assessed the activity of each catalyst for the HPRR, considering that mononuclear Cu catalysts such as Cu/TMPA produce H_2_O_2_ as an intermediate during the ORR (see RRDE data below).^70^ The HPRR activity was analyzed using 1.1 mM H_2_O_2_ to match the O_2_ concentration in PB. In both electrolytes, the Cu/TMPA catalytic wave was more positive than that of the other catalysts, especially in NaBr. This is consistent with the fact that the *E*_1/2_ values of all *para*-substituted catalysts shifted to a more negative potential than that of Cu/TMPA.

We determined the maximum turnover frequency (TOF_max_) of all catalysts for the ORR and HPRR through foot-of-the-wave analysis (FOWA) (see Supporting Information Section S8, Figures S6–S8).^101^ FOWA assumes that near the onset of catalysis, the reaction is under kinetic control and in absence of side reactions such as substrate depletion or catalyst deactivation. This method provides kinetic insights into the ORR, in which substrate depletion is significant. The TOF_max_ values for the ORR and HPRR for both PB and NaBr are shown in Table 2 and Figure 5. Note that while Cu/TMPA-(OH)_3_ was active for the HPRR in NaBr, its catalytic current showed irregular potential shifts across the measurements and was thus excluded from the FOWA.

**Table 2:** TOF_max_ and *k*_obs_^ORR^ Values Obtained from FOWA for the ORR or HPRR in the Presence of 1 atm O_2_ or 1.1 mM H_2_O_2_ and 0.3 mM [Cu^II^L]^2+^ ^a^

catalyst	electrolyte	TOF_max_^ORR^ (s^–1^)	$\frac{{TOF}_{\max}^{ORR}Cu-tmpa}{{TOF}_{\max}^{ORR}Cu-L}$	*k*_obs_^ORR^ (M^–1^ s^–1^)^d^	TOF_max_^HPRR^ (s^–1^)	$\frac{{TOF}_{\max}^{HPRR}Cu-tmpa}{{TOF}_{\max}^{HPRR}Cu-L}$
Cu/TMPA	0.1 M PB	(5.2 ± 0.6) × 10^6^ ^b^	1	1.67 × 10^9^	(2.1 ± 0.1) × 10^5^ ^c^	1
	0.1 M NaBr + 0.1 M PB	(1.24 ± 0.3) × 10^6^	1	1.13 × 10^9^	(1.84 ± 0.4) × 10^6^	1
Cu/TMPA-(OH)1	0.1 M PB	(8.64 ± 1.8) × 10^4^	60	7.9 × 10^7^	(3.39 ± 1.1) × 10^2^	619
	0.1 M NaBr + 0.1 M PB	(6.07 ± 2.1) × 10^4^	20	5.5 × 10^7^	(1.73 ± 0.9) × 10^4^	106
Cu/TMPA-(OH)2	0.1 M PB	(3.31 ± 0.4) × 10^3^	1571	3.0 × 10^6^	(5.39 ± 1.9) × 10^1^	3896
	0.1 M NaBr + 0.1 M PB	(9.30 ± 0.2) × 10^4^	13	8.5 × 10^7^	(2.39 ± 1.2) × 10^4^	77
Cu/TMPA-(OH)3	0.1 M PB	(2.14 ± 0.06) × 10^4^	243	1.9 × 10^6^	(1.03 ± 0.6) × 10^2^	2038
	0.1 M NaBr + 0.1 M PB	(6.67 ± 2.1) × 10^5^	1.86		n.d	n.d

a Calculated using *C*_O_2__ = 1.22 × 10^–3^ M (at 1.0 atm of O_2_ pressure and room temperature).

b Obtained from ref 1.

c Obtained from ref 98.

d Calculated assuming a first-order dependence on O_2_ concentration. n.d. = not determined.

**Figure 5:**
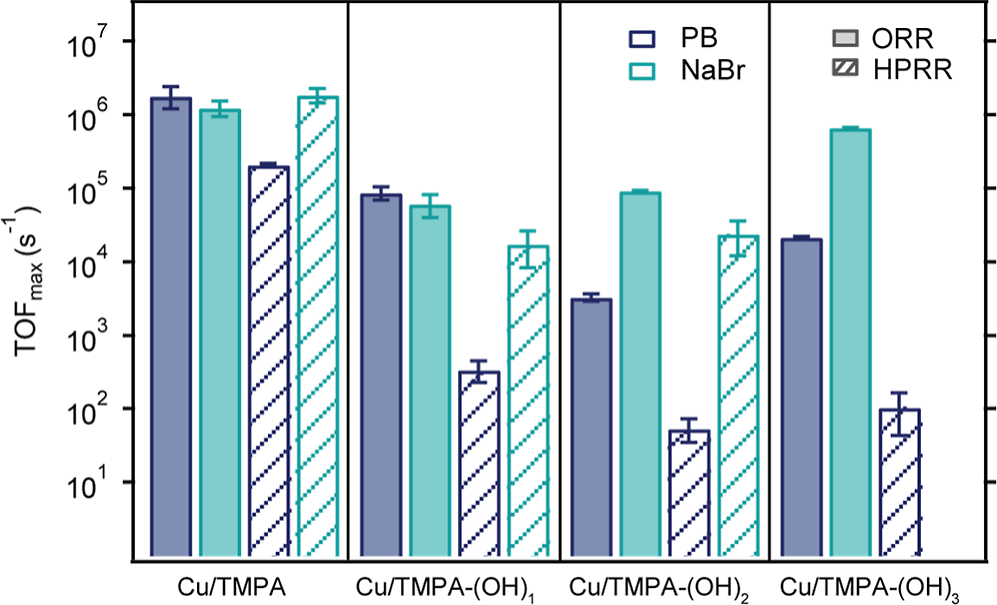
TOF_max_ values for ORR (solid bars) and HPRR (dashed bars) obtained in PB (dark blue) and NaBr (light blue) from FOWA for Cu complexes with *para*-substituted TMPA ligands compared to Cu/TMPA.

In PB, the TOF_max_ values for the *para*-substituted catalysts were at least 1 and 2 orders of magnitude lower for the ORR and HPRR, respectively, compared to those of Cu/TMPA. Cu/TMPA-(OH)_1_ catalyzed the ORR and HPRR slightly faster than Cu/TMPA-(OH)_2_ and Cu/TMPA-(OH)_3_, with Cu/TMPA-(OH)_2_ exhibiting the lowest rates among all the catalysts.

In NaBr, the TOF_max_^ORR^ values for Cu/TMPA and Cu/TMPA-(OH)_1_ were similar to those in PB. However, Cu/TMPA-(OH)_2_ and Cu/TMPA-(OH)_3_ exhibit higher TOF_max_^ORR^ values in NaBr than in PB. The HPRR trends in NaBr differed from those in PB; in PB, all the catalysts had lower HPRR rates than the ORR, whereas in NaBr, Cu/TMPA and Cu/TMPA-(OH)_1_ showed HPRR rates similar to those of the ORR. Unexpectedly, the TOF_max_ values for the HPRR in NaBr were almost as large as those for the ORR. The measurements in Figure 4 indicate that in NaBr, the onset potential of the HPRR was shifted more positively than that of the ORR for all catalysts. The TOF_max_ values obtained through FOWA are highly dependent on the precise *E*_1/2_ position of the underlying Cu^I^/Cu^II^ redox process when an EC-type mechanism is assumed. A shift in the onset potential, as observed for the HPRR in NaBr, suggests that these conditions are not necessarily fulfilled and that the FOWA analysis of the HPRR in NaBr may not be reliable and overestimated. Upon calculating k_ORR_ from the TOF_max_^ORR^ values using *C*_O_2__ = 1.1 mM and assuming a first-order dependence on O_2_, *k*_obs_ reached 1.67 × 10^9^ M^–1^s^–1^, a value 309 times that of *k*_act_ of the ATRP initiator 2-hydroxyethyl 2-bromoisobutyrate (HEBiB) (*k*_act_ = 5.4 × 10^6^ M^–1^s^–1^).^54^ Such high rate constants observed for Cu/TMPA systems demonstrate that Cu/TMPA complexes enable oxygen scavenging, which can rapidly deplete dissolved oxygen from aqueous reaction media, thereby creating favorable conditions for subsequent aerobic ATRP. Cu/TMPA can consume the typical oxygen content of air-saturated water (∼0.25 mM) within minutes, effectively transforming the reaction environment without extensive solution degassing. Additionally, this result shows that [Cu^I^TMPA]^+^ preferentially reacts with O_2_ rather than with the initiator, which explains why induction periods are sometimes observed in polymerization mixtures. The catalyst first consumes oxygen, and polymerization begins only after the O_2_ is depleted.

### 2.5 ORR Selectivity

We assessed the selectivity of the ORR via rotating ring-disk electrode (RRDE) measurements to determine whether O_2_ was reduced through a two- or four-electron pathway. In these RRDE experiments, O_2_ reduction by the copper catalysts on the GC disk resulted in the diffusion and subsequent detection of H_2_O_2_ at the Pt ring. Forced convection ensured the homogeneous transport of the catalyst and substrate to the electrode. The RRDE data for all catalysts in PB and NaBr are detailed in Section S9 of the Supporting Information. For the *para*-substituted catalysts, a linear correlation between the catalytic current and the square root of the rotation rate (ω) was observed, confirming that the electron transfer number was independent of ω. The onset potentials of the *para*-substituted catalysts were more negative than those of Cu/TMPA in both electrolytes, which is consistent with the CV results.

The RRDE measurements indicated that the ORR selectivity was strongly affected by the electronic structure of the copper site, electrolyte, and applied potential (Figure 6c). The rapid depletion of the initial O_2_ concentration at the electrode surface, followed by the depletion of H_2_O_2_ at more negative potentials, leads to an apparent H_2_O_2_ peak in the ring current near the onset of the catalytic wave. In PB, the H_2_O_2_ selectivity was in line with the maximum turnover rate of the ORR. In other words, if the TOF_max_^ORR^ is higher than TOF_max_^HPRR^, H_2_O_2_ accumulation is observed. In cases where both TOF_max_ values are high, substrate depletion occurs more rapidly, leading to relatively low H_2_O_2_ accumulation. Cu/TMPA-(OH)_2_ exhibited the lowest TOF_max_ values for ORR and HPRR, producing the highest amount of H_2_O_2_, while Cu/TMPA generated the least H_2_O_2_ due to its high TOF_max_^ORR^ and TOF_max_^HPRR^, and fast substrate depletion.

**Figure 6:**
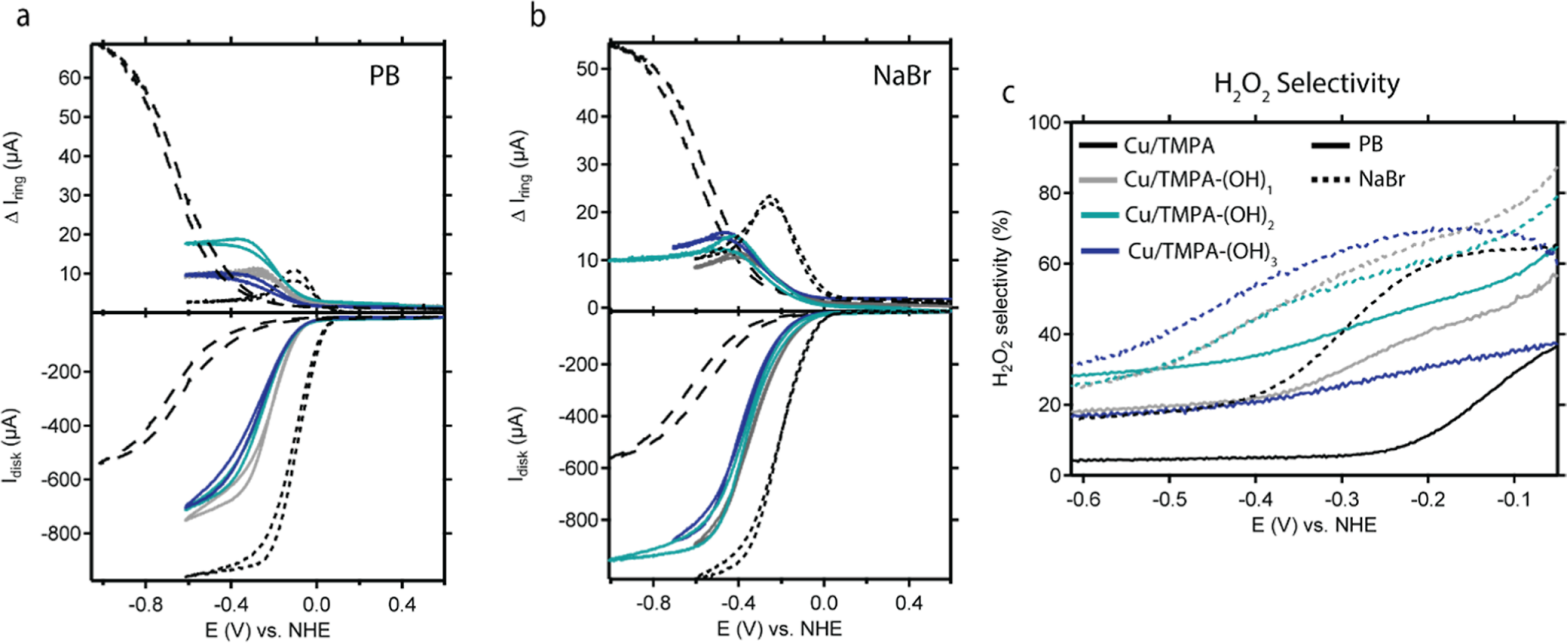
RRDE CVs recorded for Cu/TMPA-(OH)_1_ (gray), Cu/TMPA-(OH)_2_ (light blue), and Cu/TMPA-(OH)_3_ (dark blue) compared to Cu/TMPA (black dotted line) and the activity of the bare GC electrode in the absence of a catalyst (black dashed line) in PB (a) or NaBr (b). The H_2_O_2_ selectivity of all the catalysts during the ORR was determined from the RRDE measurements in both 0.1 M PB (solid lines) and 0.1 M NaBr (dashed lines) (c). Conditions: *p* = 1 atm O_2_, 0.1 M NaBr or 0.1 M PB pH 7, 0.3 mM catalyst concentration, ω = 1600 rpm, Pt ring @ 1.2 V vs RHE, υ = 0.05 V/s.

In 0.1 M NaBr, the H_2_O_2_ selectivity of all catalysts increased by up to 10% compared to that of PB, with minor differences between the catalysts. These findings differ from the FOWA results, where TOF_max_^ORR^ and TOF_max_^HPRR^ rates were shown to be more similar in NaBr compared to PB. This might stem from the overestimation of TOF_max_^HPRR^ in NaBr by the FOWA, as discussed above.

RRDE experiments were also conducted in PB and NaBr in the presence of SP to understand the reaction between H_2_O_2_ and SP (Section S10, Supporting Information). In 0.1 M PB, the addition of SP reduced the amount of H_2_O_2_ detected at the ring, indicating that part of the electrogenerated H_2_O_2_ reacted with SP during the approximately 70 ms transit from the disk to ring. Repeating these measurements in 0.1 M NaBr (with 0.01 mM PB) yielded irreproducible results, presumably due to the association/dissociation equilibria between NaBr, SP, and the Cu catalyst, preventing clear conclusions (Figure S24).

ATRP is often performed at elevated temperatures (>50 °C), particularly in emulsions. Therefore, the ORR activity of Cu/TMPA in PB was assessed at temperatures up to 60 °C (see Supporting Information Section S11). In the RRDE CV measurements at 60 °C, an additional oxidation peak at the ring emerged in the backward scan of the ORR catalysis. This peak can be attributed to the oxidation of electrodeposited Cu^0^, which formed on the disk during catalysis. This indicates that Cu/TMPA is not stable at the elevated temperatures used for ATRP. Hence, care should be taken when assessing catalyst performance at elevated polymerization temperatures. In addition, the plateau current in the RDE ORR measurements increased with increasing temperature, suggesting that the oxygen diffusion rate increased as well. Hence, at elevated temperatures, ORR catalysis is faster and therefore beneficial for the ATRP process, albeit at the expense of catalyst stability. The data presented above provide a mechanistic explanation for the often-mentioned induction period observed in nondegassed or even poorly degassed polymerization mixtures. [Cu^I^TMPA]^+^ reacts preferentially with dissolved O_2_ rather than the alkyl halide initiator (either molecular or dormant polymer chain). The induction period corresponds to the time required for [Cu^I^L]^+^ to consume all the available O_2_ via ORR, after which polymerization begins. This supports our view that the ORR is not a parasitic reaction under aerobic conditions but is, in fact, a very important component of deoxygenation in aqueous media during ATRP.

### 2.6 *e*ATRP of OEOA_480_ and OEOMA_500_ in Water

Subsequently, we polymerized OEOMA_500_ and OEOA_480_. To do so, we performed *e*ATRP using a sacrificial aluminum counter electrode (CE), known as simplified *e*ATRP (*se*ATRP),^102,103^ at *E*_app_ = *E*_1/2_ (OEOA_480_) or *E*_app_ = *E*_1/2_ + 0.06 V (OEOMA_500_), and *C*_Cu_ = 1 mM. These experimental conditions were based on previously published work on the *e*ATRP of these two monomers.^41^ We selected *C*_Br_ = 4 mM for OEOA_480_ and *C*_Br_ = 0.1 M for OEOMA_500_, as it was previously shown that these *C*_Br_ values are optimal for efficient chain deactivation and low molecular weight distribution (*M*_w_/*M*_n_).^41^ We also added 0.1 M SP to scavenge electrogenerated H_2_O_2_. In this series of experiments (Table 3 and Figure 7), the reaction volume was 20 mL, headspace volume was 80 mL, degassing time was 5 min, and N_2_ pressure was 1 bar.

**Table 3:** *e*ATRP of 10 vol % OEOA_480_ With All *Para*-substituted Copper Complexes in the Presence of 4 mM NaBr in H_2_O at 35 °C, Initiated by 2 mM HEBiB; *E*_app_ = *E*_1/2_

entry^a^	ligand	*t* (h)	conv. (%)^b^	*M*_n_^app^ × 10^–3^ ^c^	*M*_n_^th^ × 10^–3^ ^d^	*M*_w_/*M*_n_ ^e^	*k*_p_^app^ ^f^	*I*_eff_ ^g^	*Q* (C)^h^
1	TMPA	5	77	57.2	42.2	1.26	0.42	0.74	9.33
2	TMPA-(OH)_1_	5	87	56.5	47.6	1.30	0.62	0.84	6.85
3	TMPA-(OH)_2_	5	92	75.1	50.4	1.25	0.61	0.67	3.37
4	TMPA-(OH)_3_	5	86	59.1	47.0	1.41	0.51	0.79	4.74

a OEOA_480_/HEBiB/Cu­(OTf)_2_/L = 227/2/1/2, DP_T_ = *C*_OEOA_/*C*_HEBiB_ = 114, *C*_OEOA_ = 0.227 M in H_2_O; [Cu^II^L]^2+^ = 1 mM in H_2_O + 0.1 M SP.

b Determined by ^1^H NMR.

c Determined by GPC in DMF calibrated with PMMA standards.

d Theoretical molecular weight, calculated as *M*_n_^th^ = conv. × DP_T_ × MW_OEOA_ + MW_HEBiB_.

e *D̵* = *M*_w_/*M*_n_.

f The slope of the plot ln­([*M*]_0_/[*M*]).

g Initiation efficiency, determined as *I*_eff_ = *M*_n_^th^/*M*_n_^app^.

h The charge passed during polymerization.

**Figure 7:**
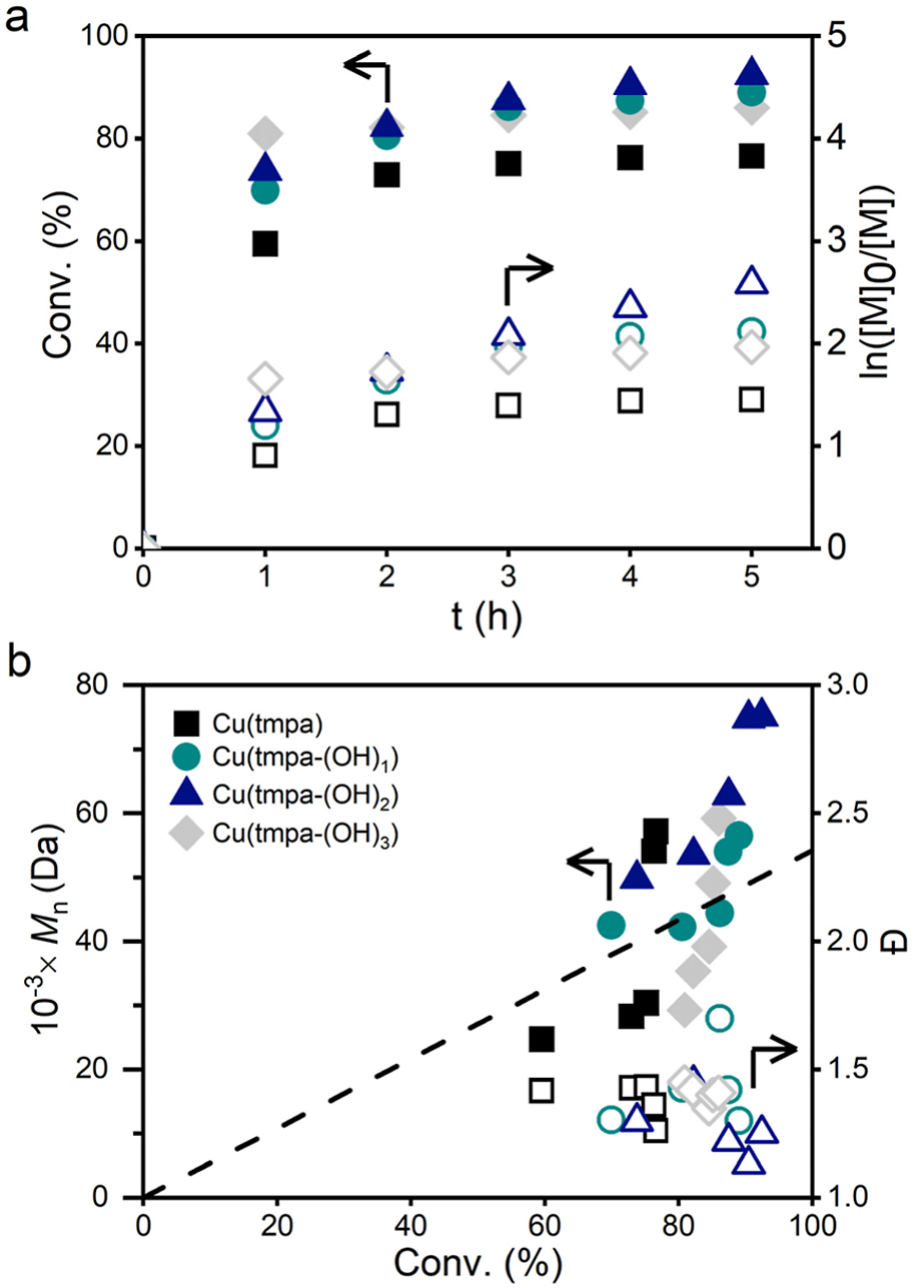
(a) Kinetic plot and (b) evolution of molecular weight and *D̵* with conversion for the *e*ATRP of OEOA_480_ at *E*_app_ = *E*_1/2_ with TMPA (■), TMPA-(OH)_1_ (teal ●), TMPA-(OH)_1_ (blue ▲), and TMPA-(OH)_3_ (gray ⧫). Filled symbols refer to the left ordinate and open symbols refer to the right ordinate. The straight black line in (b) corresponds to the theoretical molecular weights.

The conversion of OEOA_480_ ranged between 77% and 92% in 5 h for all the catalysts. At least 60% of OEOA_480_ was converted during the first hour of the polymerization reaction, after which the polymerization rate decreased (Figure 7). We obtained POEOA_480_ with a *M*_w_/*M*_n_ of 1.26–1.38. For the Cu/TMPA and Cu/TMPA-(OH)_1_ catalysts, the apparent number-average molecular weight (*M*_n_^app^) is in good agreement with the theoretical molecular weight: *M*_n_^th^ = conv. × DP_T_ × MW_Monomer_ + MW_HEBiB_, where DP_T_ = *C*_M_/*C*_RBr_ is the targeted degree of polymerization (DP_T_). A slightly higher *M*_n_^app^ than *M*_n_^th^ was observed for Cu/TMPA-(OH)_2_, the catalyst that generated more H_2_O_2_ and was the least effective for the ORR.

In an anaerobic environment, the charge passed during electrolysis quantifies radical termination events and can be used to determine the number of living polymer chains. When the Cu species concentration is low, the terminated chain fraction is determined by the charge passed (in Scheme 1, when the ORR and HPRR cycles are absent).^55,104,105^ The Cu^II^ complex forms a radical-termination event and is reduced to Cu^I^ at the WE, consuming one electron. Initially, all copper was present as [Cu^II^L]^2+^; therefore, the total charge Q was the sum of Q_0_ (initial reduction of all Cu^II^ to Cu^I^) and *Q*_T_ (reduction of Cu^II^ formed by the termination events). This is true only if no other electrochemical processes occur at the set *E*_app_ potential. During *e*ATRP in the presence of O_2_, *Q* also includes the charge consumed during the ORR and potentially the HPRR. The HPRR charge consumption may vary depending on the extent to which the HPRR competes with SP for H_2_O_2_ elimination. Although it is not possible to separately quantify the contribution of each process to the total charge, the *Q* consumption mirrors the trends of TOF_max_ for HPRR/ORR in case of *e*ATRP of OEOA_480_ (Figure 8). This also indicates that for the slower catalysts, not all O_2_ was consumed from the mixture during *e*ATRP, or less charge was consumed as the generated H_2_O_2_ reacted with SP instead of through the HPRR.

**Figure 8:**
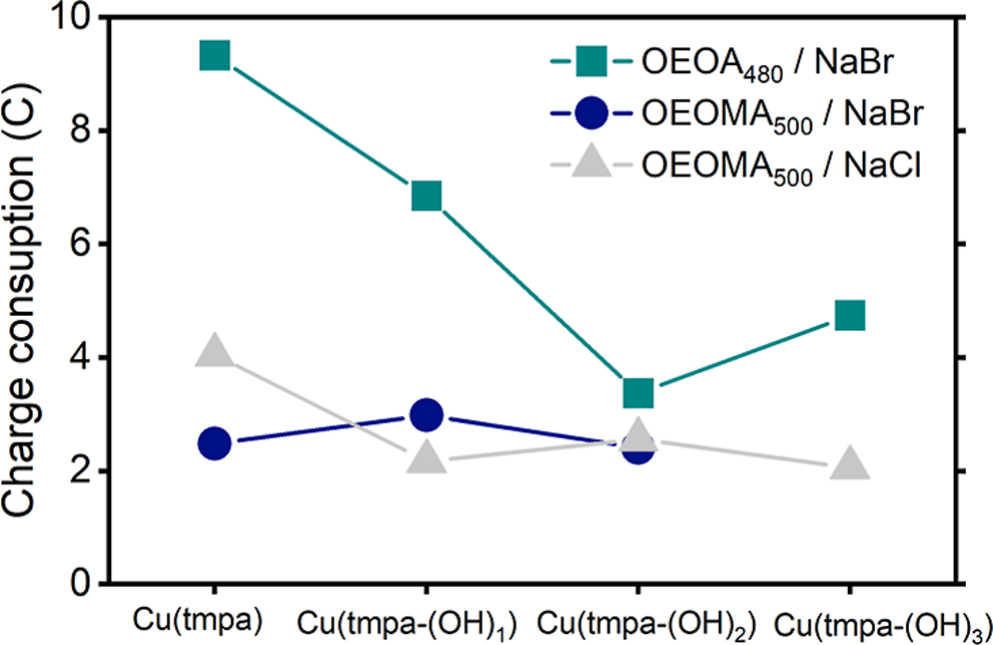
*Q* recorded during *e*ATRP of OEOA_480_ in H_2_O + 0.1 M NaBr (teal ■) and OEOMA_500_ in H_2_O + 0.1 M NaBr (blue ●) and NaCl (gray ▲). *Q* is shown for 5 h of reaction with OEOA_480_ and 3 h of reaction with OEOMA_500_.

We then proceeded with the *e*ATRP of methacrylate, OEOMA_500_. The applied potential in this case was set to *E*_app_ = *E*_1/2_ + 0.06 V. Applying a more positive potential than *E*_1/2_ generates only a small amount of [Cu^I^L]^+^ at the surface of the electrode (with a Cu^I^/Cu^II^ ratio of ∼0.1) and provides additional control over the polymerization of methacrylates.^54,97,106^ Highly active *para*-substituted Cu catalysts are expected to provide excessively high *K*_ATRP_ values for methacrylate monomers. Therefore, we investigated *e*ATRP with catalytic halogen exchange and replaced NaBr with NaCl. This method alters the dormant chain-end from C–Br to C–Cl, which should reduce *K*_ATRP_ because the reinitiation of a C–Cl dormant species is slower than that of C–Br, thereby reducing the polydispersity of the polymer. The results of these experiments are presented in Tables 4 and S28.

**Table 4:** *e*ATRP of 10 vol % OEOMA_500_ With all *Para*-substituted Copper Complexes in Presence 0.1 M NaBr or NaCl at *T* = 35 °C Initiated by 2 mM HEBiB; *E*_app_ = *E*_1/2_ + 0.06 V

entry^a^	ligand	*t* (h)	conv. (%)^b^	*M*_n_^app^ × 10^–3^ ^c^	*M*_n_^th^ × 10^–3^ ^d^	*M*_w_/*M*_n_ ^e^	*k*_p_^app^ ^f^	*I*_eff_ ^g^	*Q* (C)^h^
0.1 M NaBr									
1	TMPA	5	94	52.5	50.9	1.30	0.50	0.97	2.48
2	TMPA-(OH)_1_	3	92	142.5	49.9	1.56	0.87	0.35	3.17
3	TMPA-(OH)_2_	5	37	374.6	20.2	1.60	0.07	0.05	3.15
0.1 M NaCl									
4	TMPA	4	60	38.6	32.7	1.16	0.22	0.85	2.26
5	TMPA-(OH)_1_	4	69	58.2	37.5	1.18	0.17	0.64	2.72
6	TMPA-(OH)_2_	5	52	323.0	29.1	1.17	0.05	0.09	3.21
7	TMPA-(OH)_3_	3	55	85.7	30.5	2.82	0.26	0.36	2.51

a General conditions: OEOMA_500_/HEBiB/Cu­(OTf)_2_/L = 216/2/1/2, DP_T_ = *C*_OEOMA_/*C*_HEBiB_ = 108, *C*_OEOMA_ = 0.216 M in H_2_O + 0.1 M SP + 0.1 NaX (X = Cl, Br), unless otherwise stated; [Cu^II^L]^2+^ = 1 mM.

b Determined by ^1^H NMR.

c Determined by GPC in DMF calibrated with PMMA standards.

d Theoretical molecular weight, calculated as *M*_n_^th^ = conv. × DP_T_ × MW_OEOMA_ + MW_HEBiB_.

e *D̵* = *M*_w_/*M*_n_.

f The slope of the plot ln­([*M*]_0_/[*M*]).

g Initiation efficiency, determined as *I*_eff_ = *M*_n_^th^/*M*_n_^app^.

h The charge passed during the polymerization.

Cu/TMPA (with both NaBr and NaCl) and Cu/TMPA-(OH)_1_ (with NaCl only) produced POEOMA_500_ with a low *M*_w_/*M*_n_. The other catalysts exhibited excessive activity, resulting in POEOMA_500_ having a high *M*_w_/*M*_n_ and *M*_n_^app^, which were much higher than *M*_n_^th^. This is caused by a high concentration of propagating radicals and, in turn, a high number of termination events (because the rate of radical termination is second-order in the radical concentration), which affects *M*_w_/*M*_n_. This behavior was in good agreement with the expected faster ATRP and lower ORR and HPRR rates observed for Cu/TMPA-(OH)_2_ and Cu/TMPA-(OH)_3_ samples. The *e*ATRP of OEOMA_500_ with Cu/TMPA-(OH)_3_ was not attempted in the presence of NaBr based on the results obtained with Cu/TMPA-(OH)_1_ and Cu/TMPA-(OH)_2_, as a further increase in *K*_ATRP_ would likely exacerbate radical generation, leading to even more ill-defined polymerization.

The targeted molecular weight, determined by the ratio of the monomer to initiator concentrations (*C*_M_/*C*_HEBiB_), also affects the sensitivity of aerobic *e*ATRP to O_2_. A low *C*_M_/*C*_HEBiB_ ratio (i.e., DP_T_) reduces the inhibitory effects of O_2_ because its concentration is lower than the initiator concentration. Conversely, higher *C*_M_/*C*_HEBiB_ ratios are more sensitive to oxygen.^80^ We observed an extended inhibition period of up to 2 h before the onset of *e*ATRP of OEOMA_500_ when using Cu/TMPA-(OH)_1_ and Cu/TMPA-(OH)_2_ (Figure S29), which can be attributed to the lower TOF_max_^ORR^ compared to Cu/TMPA. Simultaneously, the higher flux of methacrylic radicals compared to acrylic radicals, and thus, a lower *C*_M_/*C*_RBr_ ratio, can also favor the polymerization-through-oxygen mechanism. This leads to the formation of unstable peroxide oligomeric species that can decompose into additional radical species, further amplifying side reactions.^107,108^ This process ultimately leads to an inhibition period in which a high number of termination events occur, thus increasing the *M*_w_/*M*_n_ and *M*_n_^app^ in poor agreement with *M*_n_^th^, as observed for the polymerization of OEOMA_500_ (Figure S29). In the case of acrylates, which have a lower *k*_act_ than methacrylates, the radical flux is much lower, resulting in a lower *M*_w_/*M*_n_ than *M*_n_^app^, which is in good agreement with *M*_n_^th^.

For the *e*ATRP of OEOMA_500_, we observed lower charge consumption than that of OEOA_480_. This is not only because of the shorter reaction times but also because of the suppressed ORR activity in the presence of higher *K*_ATRP_ values (more [Cu^I^L]^+^ is engaged in ATRP than in the ORR). This is further supported by the fact that no correlation exists between Q and TOF_max_^ORR^, as observed for the *e*ATRP of OEOA_480_ (Figure 8).

Two chain extension experiments were performed to assess the livingness of the POEOA_480_-Br macroinitiators. The macroinitiators were prepared using Cu^II^/TMPA and Cu^II^/TMPA-(OH)_1_, with *M*_n_^app^ = 5.8 kDa and *M*_w_/*M*_n_ = 1.32 (Cu^II^/TMPA) and *M*_n_^app^ = 5.6 kDa and *M*_w_/*M*_n_ = 1.38 (Cu^II^/TMPA-(OH)_1_), respectively. Specifically, we extended POEOA_480_-Br macroinitiators with methacrylate OEOMA_500_ under catalytic halogen exchange (cHE) conditions. cHE is suitable when a less reactive acrylate is going to be extended with a more reactive methacrylate, by adding 0.1 M NaCl to the reaction mixture. Both chain extension experiments showed the retention of the chain-end functionality (C–Br terminal) of POEOA_480_-Br. We obtained the block copolymers P­(OEOA_480_)-*b*-P­(OEOMA_500_)-Cl with *M*_n_^app^ = 84.4 kDa and *M*_w_/*M*_n_ = 1.24 (Cu^II^/TMPA) and *M*_n_^app^ = 83.0 kDa and *M*_w_/*M*_n_ = 1.50 (Cu^II^/TMPA-(OH)_1_), respectively (Figure 9).

**Figure 9:**
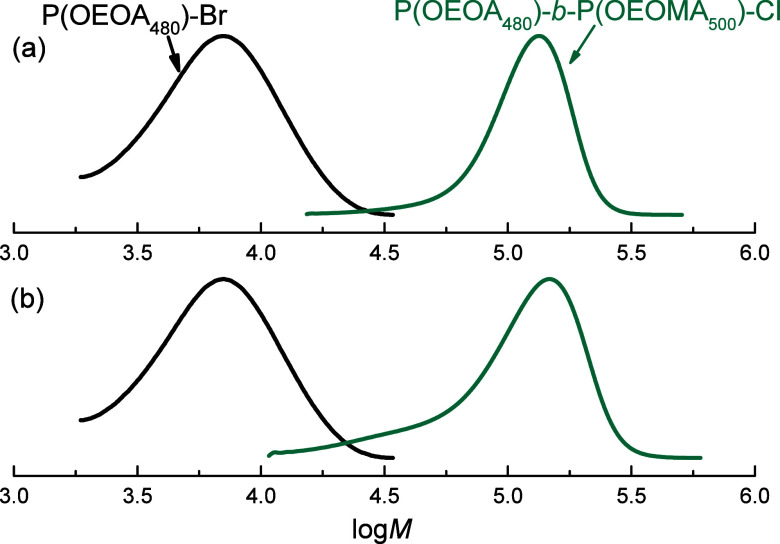
Molecular weight distributions recorded during the chain extension experiments by *e*ATRP showing P­(OEOA_480_)-Br macroinitiators before (black —) and after being extended using OEOMA_500_ in the presence of 0.1 M NaCl to produce the block copolymers P­(OEOA_480_)-*b*-P­(OEOMA_500_)-Cl (teal —): (a) using Cu^II^/TMPA and (b) Cu^II^/TMPA-(OH)_1_ as catalysts. Conditions: *C*_OEOMA500_/*C*_Cu_/*C*_Ligand_/*C*_NaCl_/*C*_(POEOA480)‑Br_ = 432:1:2:100:4; *T* = 35 °C, *E*_app_ = *E*_1/2_ + 0.06 V.

## 3 Factors to be Considered during Competitive ATRP and ORR/HPRR Catalysis

Understanding the competitive catalysis between ATRP and ORR/HPRR is crucial for enhancing the applicability of ATRP in aerobic environments. Thus, rationalizing the ORR/HPRR rates of Cu/TMPA and the structures of its *para*-substituted analogs can provide direction for future efforts in this competitive catalysis scenario. Our analysis of the ORR/HPRR and *e*ATRP is summarized in Scheme 4 and discussed below.

**Scheme 4:**
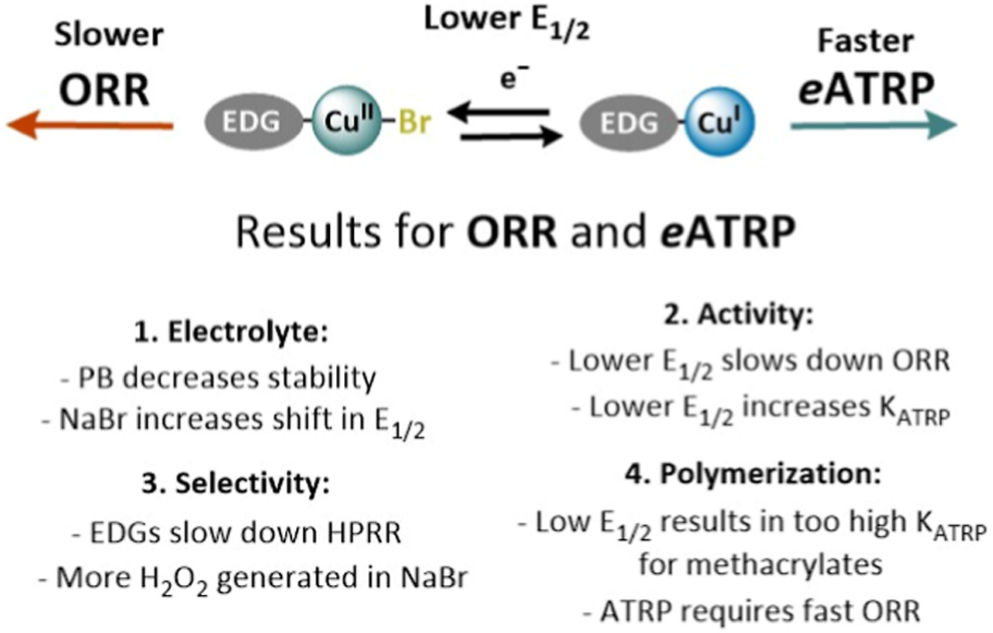
Overview of the Main Observations Regarding the Electrolyte, Catalyst Activity, Selectivity, and Polymerization for Both ORR and *e*ATRP Upon the Introduction of EDGs on the Ligand Framework to Lower the *E*_1/2_ of Cu Catalysts

### 3.1 Effect of Electrolyte

The supporting electrolyte affects the stability and redox properties of Cu complexes because phosphate, bromide, and pyruvate anions can coordinate with Cu center. Cyclic voltammetry in 0.1 M PB, the electrolyte commonly used in ORR/HPRR experiments, showed that the shift in the *E*_1/2_ of the redox couple was small upon addition of EDGs to TMPA, whereas in NaBr and SP, which are usually present in aqueous aerobic ATRP, the *E*_1/2_ of the redox couple is shifted significantly. Our work also indicates that the addition of EDGs decreases the stability of the catalyst in PB, whereas the presence of NaBr does not cause any destabilization. Furthermore, the presence of monomer also affected the redox properties of the catalysts, which is in line with previous reports.^41^

### 3.2 Effect of EDGs

The structure of the ligand significantly influences the ORR/HPRR and ATRP catalytic activities. The addition of EDGs to TMPA decreased *E*_1/2_ and increased *k*_act_ and, in turn, *K*_ATRP_, as reported in previous studies.^90,93–95^ As a result, Cu/TMPA-(OH)_1_ and Cu/TMPA-(OH)_2_ provided polyacrylates with low *M*_w_/*M*_n_, but their activity for the ATRP of methacrylates was too high, and polymethacrylates were obtained with high *M*_w_/*M*_n_. During *e*ATRP, the TOF_max_ values of the ORR/HPRR should be maximized. According to a previous study by one of our groups, a more negative *E*_1/2_ would result in a higher binding energy of O_2_ to the Cu^I^ site and increased ORR activity.^66^ Contrary to these expectations, the addition of EDGs to the TMPA ligand led to a decrease in the ORR and HPRR rates. We propose that this is due to the excessive stabilization of the ORR intermediates and O_2_ binding by the electron-rich environment of *para*-substituted catalysts. Thus, Cu/TMPA appears to be the optimal catalyst when the ORR/HPRR and ATRP processes coexist in water. Consequently, slow ORR/HPRR affects the polymerization reaction in the presence of O_2_. Ultimately, *para*-substitution has a divergent impact on the ORR/HPRR and ATRP activities, making it challenging to optimize both processes simultaneously.

### 3.3 Effect on ORR Selectivity

The TOF_max_ values obtained by FOWA show that the ORR and HPRR rates are strongly impacted by the number of EDGs, eventually leading to catalysis of the ORR with an increased selectivity toward H_2_O_2_ in both PB and NaBr, when the number of EDGs on the TMPA ligand increases. In line with this, RRDE experiments showed that the *para*-substituted complexes catalyzed the ORR with increased selectivity toward the accumulation of H_2_O_2_, especially in NaBr. This highlights the need for sodium pyruvate as a H_2_O_2_ scavenger in ATRP to prevent side reactions caused by H_2_O_2_.

### 3.4 Effect of Charge Consumption

In anaerobic environments, the charge consumption during ATRP can be correlated with radical termination. This is especially important for scales much larger than those investigated in this work. When O_2_ is present, the ORR and HPRR consume charge; hence, it is impossible to quantify radical termination unambiguously. However, the measured charge consumption can still provide insights into the polymerization process and the extent of ORR and HPRR catalysis. During the *e*ATRP of OEOA_480_, we observed that Cu/TMPA, having the highest TOF_max_, exhibited a higher charge consumption than the other catalysts, suggesting increased reduction of oxygen and hydrogen peroxide (Table 3 and Figure 8). Additionally, the *M*_w_/*M*_n_ of POEOA_480_ was the lowest for Cu/TMPA, suggesting that the fast and efficient removal of O_2_ and H_2_O_2_ is beneficial for ATRP, as previously reported. An even faster ORR and HPRR could potentially eliminate the need for sodium pyruvate. In contrast, all the catalysts performed the *e*ATRP of OEOMA_500_ with much higher activity and lower charge consumption, but in the presence of an induction period. We think that these high polymerization rates further suppress the ORR/HPRR, resulting in uncontrolled polymerization in the presence of oxygen.

### 3.5 Diffusion Limitations

Both ATRP and ORR are rapid reactions in water and can approach diffusion-limited rates under ideal conditions. The ORR rate is dependent on the concentration of O_2_ and the catalyst and follows the Bell-Evans–Polanyi relationship, where ln­(TOF) scales with the redox potential of the Cu­(II)/Cu­(I) couple.^109^ This suggests that, at first, the synthesis of Cu complexes with more negative Cu­(II)/Cu­(I) redox potentials may further enhance their TOFs. However, at such high rates, the ORR becomes diffusion limited. Diffusion limitations are also likely to become significant during ATRP because of the significant increase in viscosity due to polymer formation. However, this has not been extensively discussed in the literature, and there is a noticeable lack of reports on how viscosity affects *k*_act_, *k*_deact_ and *K*_ATRP_.

Assuming that [Cu^I^TMPA]^+^ and [Cu^II^TMPA]^2+^ have the same diffusion coefficient, a diffusion-controlled rate constant *k*_d_ of ∼8 × 10^10^ M^–1^ s^–1^ can be estimated. In polymerization mixtures, which become increasingly viscous, the diffusion-limited rates are expected to decrease significantly. In viscous media, the interplay between ORR and *e*ATRP is likely to be dominated by diffusion kinetics, highlighting the challenge of maintaining the structure–activity correlations of Cu catalysts optimized for both ORR and *e*ATRP under such conditions.

Nevertheless, our findings suggest that the *k*_obs_ values for ORR are already near the diffusion-controlled limit, especially in the case of Cu^I^/TMPA, which is a catalyst that best balances both ORR and ATRP kinetics. These results provide a strong motivation for conducting detailed ORR structure–activity correlations to identify the optimal catalysts for both ATRP and ORR. Understanding these correlations is central to designing catalysts that remain effective under diffusion-constrained conditions and enable more efficient polymerization processes across a broader range of monomers in aerobic media.

## 4 Conclusions

In this work, we investigated and rationalized the competitive catalysis between ATRP and ORR/HPRR in water using three newly synthesized *para*-substituted TMPA ligands with varying numbers of *N*-methylaminoethanol EDGs. Unlike hydrophobic pyrrolidine, *N*-methylaminoethanol renders these catalysts hydrophilic and, therefore, suitable for aqueous *e*ATRP. Systematic electrochemical investigations clarified the impact of ligand structure on the redox properties and catalysis of both ORR/HPRR and *e*ATRP, showing a delicate balance between ligand design and catalytic outcomes. The introduction of *para*-EDGs oppositely changed the ATRP and ORR/HPRR directions, enhancing the ATRP activity while reducing the ORR/HPRR rates compared to those of Cu/TMPA. *Para*-EDGs also increased the ORR selectivity toward H_2_O_2_ in the presence of NaBr, justifying the need for sodium pyruvate as a hydrogen peroxide scavenger during ATRP. The observed induction periods in aerobic ATRP are mechanistically explained by the preferential O_2_ reduction by [Cu^I^TMPA]^+^, which precedes radical initiation. This confirms that the ORR serves as an intrinsic deoxygenation step in *e*ATRP and is not merely a competing side reaction. *Para*-substituted Cu catalysts can be used for the *e*ATRP of acrylates; however, their activity is too high to achieve the controlled polymerization of methacrylates. This work advances our understanding of Cu-mediated molecular ORR/HPRR in the context of aqueous ATRP, supports the rational design of Cu catalysts for scalable ATRP in aerobic aqueous systems, and identifies the factors that need to be considered for the future development of Cu-based catalysts for ORR and ATRP.

## 5 Materials and Methods

All information pertaining to the materials and methods used in this study is reported in the Supporting Information.

## Supplementary Material



## Notes

The Supporting Information includes detailed experimental procedures, methods, and instrumentation (synthesis of *para*-substituted TMPA Ligands and synthesis of copper complexes), an overview of all redox properties, electrochemical measurements (scan rate dependence measurements, CV measurements, foot-of-the-wave analysis, rotating ring-disk electrode experiments, Koutecky–Levich analysis, catalysis in the presence of sodium pyruvate, catalysis at higher temperatures), additional polymerization details, and Supporting References (PDF)

## Abbreviations

Δ*E*_p_: peak-to-peak separation
ATRP: atom transfer radical polymerization
CE: counter electrode
DP_T_: degree of polymerization (targeted)
*E*_1/2_: half-wave potential
*E*_onset_: onset potential
*E*_pa_: anodic peak potential
*E*_pc_: cathodic peak potential
*e*ATRP: electrochemically mediated ATRP
EDGs: electron donor group(s)
EWGs: electron withdrawing group(s)
FOWA: foot of the wave analysis
HEBiB: 2-hydroxyethyl α-bromoisobutyrate
HPRR: hydrogen peroxide reduction reaction
NHE: normal hydrogen electrode
OEOA_480_: poly­(ethylene glycol) methyl ether acrylate 480
OEOMA_500_: poly­(ethylene glycol) methyl ether methacrylate 500
ORR: oxygen reduction reaction
RDRP: reversible deactivation radical polymerization
RRDE: rotating ring-disk electrode
SCE: saturated calomel electrode
TMPA: tris­(2-pyridylmethyl)­amine
