# Supplementary material for: Interplay between the Oxygen Reduction Reaction and Atom Transfer Radical Polymerization with Molecular Cu-Based Catalysts in Water
Source: ACS Catal. 2025 Aug 7;15(16):14548–63. doi: 10.1021/acscatal.5c04928 (PMC12818763; doi:10.1021/acscatal.5c04928)
Supplement: Supplementary file 1 [file cs5c04928_si_001.pdf]

Supporting Information for

Interplay between the Oxygen Reduction Reaction and Atom Transfer Radical Polymerization with Molecular Cu-based Catalysts in Water

Phebe H. van Langevelde[a], Katarina Ležaić[a], Jorge F. J. Coelho[b,c], Dennis G. H. Hetterscheid[a] and Francesco De Bon[b*]*

[a] Leiden University, Leiden Institute of Chemistry, 2300 RA, Leiden, The Netherlands

[b] CEMMPRE, ARISE, Department of Chemical Engineering, University of Coimbra, Rua Sílvio Lima, Pólo II, 3030-790 Coimbra, Portugal

[c] IPN, Instituto Pedro Nunes, Associação para a Inovação e Desenvolvimento em Ciência e Tecnologia, Rua Pedro Nunes, 3030-199 Coimbra, Portugal

**Correspondence to: Dennis G.H. Hetterscheid (d.g.h.hetterscheid@chem.leidenuniv.nl) and Francesco De Bon (francesco@eq.uc.pt)*

Contents

1. Experimental details and methods 3
1.1 General 3
1.2 Electrochemistry Experiments 3
1.2.1 General Cleaning Procedure 3
1.2.2 Electrochemical measurements in absence of monomer 4
1.2.3 Rotating Ring-Disk Electrode (RRDE) Measurements 4
1.3 Electrochemically Mediated ATRP 5
1.3.1 Typical procedure for eATRP of $OEOMA_{480}$ or $OEOMA_{500}$ using Cu/TMPA 5
2. Synthesis of *para*-substituted TMPA Ligands 7
3. Synthesis of Copper Complexes 9
4. Overview of all Redox Properties 11
5. Scan Rate Dependence Measurements 12
6. CV Measurements of Cu/TMPA-$(OH)_3$ 15
7. CV Measurements in Presence of both PB and NaBr 16
8. Foot-of-the-wave Analysis 16
8.1 ORR Measurements in PB 18
8.2 HPRR Measurements in PB 21
8.3 ORR Measurements in NaBr 24
8.4 HPRR Measurements in NaBr 28
9. Rotating Ring-Disk Electrode Experiments 31
9.1 Koutecky-Levich Analysis 31
10. Catalysis in presence of Sodium Pyruvate 36
11. Catalysis at Higher Temperatures 37
12. Electrochemical Data Recorded against SCE before Polymerization 38
13. *se*ATRP of $OEOMA_{500}$ in the presence of NaCl 41
14. References 42

1. Experimental Details and Methods

1.1 General.

Commercially available solvents and reagents were used as received, without additional purification. Copper(II) trifluoromethanesulfonate ($Cu(OTf)_2$, 99 %) was obtained from Alfa Aesar. The acetonitrile was dried using a PureSolve 400 solvent dispenser. All electrolyte solutions were prepared using Milli-Q Ultrapure grade water, Na_2HPO_4 (Honeywell Fluka traceSELECT, ≥99.999 %), NaH_2PO_4 (Merck Suprapur, 99.99 %) and NaBr (Thermo Scientific Puratronic, 99.9955 %), $NaOH{\cdot}H_2O$ (≥99.9995% TraceSELECT, Honeywell Fluka), H_2SO_4 (96% in H_2O, Merck, Suprapur). Hydrogen peroxide (30 wt%, 9.8 M solution in water) was provided by Merck. Sodium pyruvate (97%) was purchased from TCI Chemicals and was stored at 4 °C. Tris(2-pyridylmethy)amine (TMPA) was prepared by reacting bis(2-pyridylmethyl)amine (TCI Chemicals, >98%) with pyridine 2-carboxaldehyde (Sigma Aldrich, 99%) in $CHCl_3$ followed by reduction with sodium triacetoxyborohydride (Sigma Aldrich, 97%), according to a published procedure.[1] Polyethylene glycol methyl ether acrylate ($OEOA_{480}$, 99%) and methacrylate ($OEOMA_{500}$, 99%) were purchased from Sigma Aldrich and percolated in a basic alumina column to remove the radical inhibitor before the polymerizations. They were then stored at -20 °C. The ATRP initiator 2-hydroxyethyl 2-bromoisobutyrate was prepared according to a published procedure.[2]

The pH values of the prepared electrolyte solutions were measured using a Hanna Instruments HI 4222 pH meter calibrated with IUPAC standard buffers. The ^1H and ^{13}C NMR spectra were collected using a 400 MHz Bruker Avance III HD spectrometer, equipped with a 5 mm TXI triple resonance detection probe, in deuterated chloroform ($CDCl_3$) and dimethyl sulfoxide (DMSO-d_6). Residual $CHCl_3$ and DMSO peaks (δ = 7.26 ppm and δ = 2.50 ppm, respectively) were used as an internal reference. Elemental analysis was performed using a Mikroanalytisches Laboratorium Kolbe, Germany. $[Cu(TMPA)MeCN](OTf)_2$ (TMPA = tris(2pyridylmethyl)amine) was synthesized as published previously.[3]

1.2 Electrochemistry Experiments

1.2.1 General Cleaning Procedure

All glassware used in the electrochemical measurements was cleaned by regular overnight soaking in a solution of H_2SO_4 (0.5 M) and $KMnO_4$ (1 g/L), after which all glassware was rinsed five times with Milli-Q water. Next, all glassware was soaked in a diluted solution of H_2SO_4 and H_2O_2 in Milli-Q water for 30 min. Thereafter, the glassware was boiled in Milli-Q water thrice. Before every experiment, the glassware was boiled for an additional time in Milli-Q water.

1.2.2 Electrochemical measurements in absence of monomer

All electrochemical experiments were carried out in a custom-build 10 mL glass cell using a three-electrode setup. Measurements were recorded on an Autolab PGSTAT 12, 204 and 128N potentiostats in combination with Autolab NOVA 2 software. A PEEK-encapsulated glassy carbon (GC) electrode (*A*= 0.071 cm^2, Metrohm) was used as the working electrode. This electrode was polished before each measurement using a Struers LaboPol-30 polishing machine. Polishing was performed with 1.0 µm diamond polishing for 60 s, followed by a 0.04 µm silica suspension for 90 s on Dur-type polishing cloths. After polishing, GC was sonicated in Milli-Q water for 15 min. After every polishing step, the CV of the GC electrode was recorded in the electrolyte solution in the absence of a catalyst to verify its cleanliness. A gold wire was used as the counter electrode. This electrode was flame-annealed and rinsed with Milli-Q water daily. A Reversible Hydrogen Electrode (RHE) made from a platinum mesh and hydrogen gas bubbling through the electrolyte solution was used as the reference electrode in the presence of a buffer. RHE was connected to the main cell compartment using a Luggin capillary. In the sodium bromide solutions, a double-junction Ag/AgCl reference electrode filled with 3 M KCl from Metrohm was used. All gases for the electrochemical measurements (Ar, H_2, and O_2) were supplied by Linde and bubbled through the solution for a minimum of 15 min before the measurement, and a flow of the desired gas over the solution was applied during all measurements. The pH of all NaBr electrolyte solutions was adjusted to 7 before every measurement using concentrated H_2SO_4 and NaOH solutions. After pH adjustment, the desired catalyst was dissolved in the electrolyte. For Cu/TMPA-$(OH)_1$, Cu/TMPA-$(OH)_2$, and Cu/TMPA-$(OH)_3$, a concentrated catalyst solution (30 mM) was prepared in MeCN, and the desired amount was added to the electrolyte solution.

1.2.3 Rotating Ring-Disk Electrode (RRDE) Measurements

A custom-made electrochemical cell with a minimal volume of 40 ml was used for the RRDE experiments. A 5 mm glassy carbon disk electrode (*A* = 0.196 cm^2) combined with a platinum ring in an E6R1PK ChangeDisk configuration from Pine was used in combination with an MSR rotator from Pine. The ring and disk were polished separately before every experiment using a Struers LaboPol-30 polishing machine, 1.0 µm diamond and 0.04 µm silica suspensions for 2 minutes on Dur-type polishing cloths. Polishing was followed by sonication in Milli-Q water for 15 min. The counter and reference electrodes were prepared as previously described. The gold counter electrode was separated from the main compartment by using a glass frit. Before each experiment, the electrolyte solutions were saturated with oxygen for 20 min, and oxygen gas was bubbled through the solution during the experiment.

For the experiments with variable temperatures, a smaller custom-made electrochemical cell (V = 25 mL) was used, which was placed in a heating bath and heated to the desired temperature while the temperature was monitored with a thermometer.

1.3 Electrochemically Mediated ATRP

*se*ATRP at a scale of 20 mL were performed in a Pine Research 5-neck glass cell, equipped with three electrodes, and connected to a BioLogic SP150 potentiostat/galvanostat, connected to a computer with EC-Lab software (BioLogic, France). Cyclic voltammetry (CV) experiments were performed on a glassy carbon (GC) disk electrode (Pine Research), Pt wire counter electrode (CE, Pine Research), and saturated calomel electrode (SCE). Before each experiment, the GC disk was cleaned by polishing with a 0.25 µm alumina abrasive paste, followed by a 5 min ultrasonic rinse in ethanol. For electrolysis, the working electrode (WE) was a Pt mesh (Alfa Aesar, 99.9% metal basis) with a geometric area of approximately 6 cm^2, which was cleaned by sonication in concentrated (65%) HNO_3 before each experiment and rinsed with abundant water and acetone. The CE was an aluminum wire immersed directly in the polymerization mixture. The cell was maintained at the desired temperature (35 °C) using a thermostatic water bath. All electrolyses were performed after venting the headspace of the electrochemical cell (~80 cm^3) with N_2. Stirring was magnetically provided with a 2 cm PTFE-coated magnet at 700 rpm during electrolysis. The molecular weight parameters of $POEOA_{480}$ and $POEOMA_{500}$ were determined using a size exclusion chromatography (GPC/SEC) setup from Viscotek (Viscotek TDAmax) equipped with a differential viscometer (DV), right-angle laser-light scattering (RALLS, Viscotek), and refractive index (RI) detectors. The column set consisted of a PLgel 5 µm guard column, followed by one Viscotek T5000 column and one Viscotek T4000 column. The dual-piston pump was set at a flow rate of 1 mL/min. The eluent (DMF + 0.03% LiBr) was filtered through a 0.2 µm filter. The analysis was performed at 60 °C using an Elder CH-150 heater. Prior to injection (100 µL), the samples were filtered through a 0.2 µm pore size PTFE membrane. The system was calibrated using six narrow poly(methyl methacrylate) standards (M_n = 4520–96000 Da). The molecular weight (M_n^{GPC}) and $Đ$ ($Đ = M_w/M_n$) of the synthesized polymers were determined by multidetector calibration (dn/dc= 0.0445 for $POEOA_{480}$ and 0.0487 for $POEOMA_{500}$) using OmniSEC software version 4.6.1.354. Monomer conversion was determined by ^{1}H-NMR spectroscopy using a Bruker Avance III HD 400 MHz instrument with D_2O as the solvent and 2 vol% DMF as the internal standard.

1.3.1 Typical procedure for seATRP of $OEOMA_{480}$ or $OEOMA_{500}$ using Cu/TMPA or para-substituted ligands

The electrochemical cell was placed in a thermostatic bath and equipped with an SCE reference electrode, a 14 cm aluminum counter electrode, and both the GC disc and Pt mesh working electrodes. Sodium pyruvate (0.220 g, 2 mmol), NaBr (0.205 g, 2 mmol for $OEOMA_{500}$ or 8.2 mg, 0.08 mmol for $OEOA_{480}$), a pH = 7.4 phosphate buffer (10 mM) and $OEOA_{480}$ (V_{total} = 20 mL) were inserted into the cell.

Then, $Cu^{II}(OTf)_2$ (5.4 mg, 0.015 mmol), TMPA (9.6 mg, 0.032 mmol) (or any of the other ligands, 2 eq with respect to Cu^{II}) were added in solution. After stirring and degassing the headspace, the CV of the catalyst was recorded to measure its standard reduction potential and to select the E_{app} value for electrolysis. Then, HEBiB (8.44 mg, 0.04 mmol) was introduced, and a CV was recorded to observe the catalytic effect. Finally, the headspace was degassed for 5 min before applying the selected E_{app} value and starting electrolysis. The samples were withdrawn periodically during polymerization and analyzed using NMR and GPC.

2. Synthesis of *para*-substituted TMPA Ligands

Synthesis of the TMPA-(Cl)$_n$ (n = 1, 2, 3) precursors.

The 4-chloro substituted precursors were synthesized according to published procedures.[4] The synthesis of TMPA-(Cl)$_2$ is provided as an example. Briefly, to 2-aminomethyl pyridine (0.5 g, 0.452 mL, 4.65 mmol) in dry $CHCl_3$ are added 1.316 g (9.30 mmol) of 4-chloropyridine-2-carboxaldehyde under positive N_2 pressure. The mixture was then stirred at room temperature for three hours. The mixture was then cooled to 0 °C in an ice/water bath and sodium triacetoxyborohydride (2.07 g, 9.76 mmol) was added slowly. The mixture was allowed to rise to room temperature and was stirred overnight. The mixture was then transferred to a separation funnel and washed three times with a saturated $NaHCO_3$ solution, followed by washing with water and brine. The organics were collected and dried using anhydrous sodium sulfate. The solvent was removed by rotary evaporator, and the title compound was obtained as a viscous yellowish oil (1.352 g, 81 %). ^{1}H-NMR matched literature results.[5]

Synthesis of TMPA-(OH)$_n$ (n = 1, 2, 3) ligands.

p-substituted TMPA-(OH)$_n$ were prepared according to a published procedure with modifications, replacing pyrrolidine with *N*-methylaminoethanol.[6]

Synthesis of (4-(aminomethyl)ethanolpyridyl)-bis-N-pyridylmethylamine TMPA-(OH)$_1$

0.3215 g of the crude *p*-substituted TMPA-Cl$_1$ (0.97 mmol) was dissolved in excess *N*-methylaminoethanol (5.467 mL, 48.62 mmol) while stirring into an Ace Glass pressure tube. Thereafter, the atmosphere was exchanged with pure N_2 via vacuum/N_2 cycling. After sealing the tube in N_2, the mixture was heated to 125 °C for 18 h with stirring in a silicone oil bath. The day after, the mixture was poured into $CHCl_3$ and extracted three times with H_2O and twice with brine. The chloroform layer was separated, dried with Na_2SO_4, filtered, and the solvent evaporated under reduced pressure. The ligand was collected as a yellowish powder (yield = 95 %, 0.336 g) after standing overnight under a fume hood. ^{1}H-NMR ($CDCl_3$) ∂ 8.42-8.41 (d, 2H), 7.94-7.92 (d, 1H), 7.55-7.51 (m, 2H), 7.40 (m, 4H), 7.04 (m, 1H), 6.94 (s, 1H), 3.74-3.70 (s, 4+2H), 3.6 (2H), 3.48-3.45 (m, 2H), 2.09 (s, 3H); ^{13}C-NMR (100 MHz, $CDCl_3$, ppm): δ = 158.88 (s, Ar-CH), 155.2 (s, Ar-CH), 148.71 (s, Ar-CH), 136.76 (d, Ar-CH), 130.31 (s, Ar-CH), 122.92 (s, Ar-CH), 108.4 (d, Ar-CH), 63.8 (s, CH_2), 60.43 (s, CH_2), 58.6 (s, CH_2), 41.9 (s, CH_3).

Elemental analysis: calculated (%) for $C_{21}H_{25}N_5O$: C 69.40, H 6.93, N 19.27; determined (%): C 69.41, H 6.99, N 19.33.

Synthesis of bis(4-(aminomethyl)ethanolpyridyl)-N-pyridylmethylamine TMPA-(OH)$_2$

1 g of the crude *p*-substituted TMPA-Cl_2 (2.78 mmol) was redissolved in excess *N*-methylaminoethanol (7.83 mL, 97.42 mmol) while stirring into an Ace Glass pressure tube. The atmosphere was exchanged with pure N_2 via vacuum/N_2 cycling. After sealing the tube in N_2, the mixture was heated to 125 °C for 18 h with stirring in a silicone oil bath. The day after, the mixture was poured into $CHCl_3$ and extracted three times with H_2O and twice with brine. The chloroform layer was separated, dried with Na_2SO_4, filtered, and the solvent evaporated under reduced pressure. The ligand was collected as a yellowish powder (yield = 86 %, 1.076 g) after standing overnight under a fume hood. ^{1}H-NMR (DMSO-d_6) ∂ 8.50 (d, 1H), 8.01-8.00 (d, 2H), 7.77 (m, 1H), 7.62-7.60 (d, 2H), 7.25 (m, 2H), 6.92 (d, 1H), 6.51-6.49 (m, 1H), 3.54 (s, 2H), 3.6 (4H), 3.42-3.45 (m, 4H), 2.97 (s, 6H); ^{13}C-NMR (DMSO-d_6) ∂ 159.69 (s, Ar-CH), 158.7 (s, Ar-CH), 154.5 (s, Ar-H), 148.6 (s, Ar-CH), 137.13 (d, Ar-CH), 122.7 (d, Ar-CH), 105.7 (d, Ar-CH), 59.8 (s, CH_2), 58.49 (s, CH_2), 53.5 (s, CH_2), 39.9 (s, CH_3).

Elemental analysis – calculated (%) for $C_{24}H_{32}N_6O_2$: C 66.03, H 7.39, N 19.25, found (%): C 66.10, H 7.43, N 19.29.

Synthesis of tris 2-(4-(aminomethyl)ethanolpyridylmethyl)amine TMPA-(OH)$_3$

TMPA-Cl_3 (0.518 g, 1.32 mmol) was added to excess 2-(aminomethyl)ethanol (3.7 mL, 46.05 mmol) while stirring under N_2 atmosphere inside an Ace Glass pressure tube. The homogeneous brown mixture was heated at 125 °C for 18 h in a silicone oil bath and then cooled to room temperature. The mixture was diluted with water and added dropwise to ice-cold acetone (0 °C) under gentle stirring. The off-white solid precipitating out was the ligand TMPA-(OH)$_3$, leaving a dark brown liquor of impurities. The liquor was removed carefully by suction with a syringe, the solid washed once with cold acetone (0 °C), and dried open to air in a fume hood. The recovered liquor was again concentrated under reduced pressure and added to ice-cold acetone, which afforded another small off-white solid crop of ligand. **NOTE:** Of the many workups attempted, only the addition of cold acetone to the mother liquor effectively afforded the target compound. Alternatively, we also found that the crude mixture could be diluted with water, layered slowly with acetone at room temperature, and allowed to cool in a refrigerator (4 °C) for 2 days. The ligand precipitated out of the mixture as an off-white solid. Yield = 77 %. ^{1}H-NMR (DMSO-d_6) ∂ 8.50 (d, 3H), 6.80 (d, 3H), 6.21 (dd, 3H), 3.86 (t, 6H), 3.76 (s, 6H), 3.59 (m, 6H), 3.00 (s, 9H); ^{13}C-NMR (DMSO-d_6) ∂ 159.28 (s, Ar-CH), 149.9 (s, Ar-CH), 109.71 (s, Ar-CH), 107.1 (s, Ar-CH), 59.8 (s, CH_2), 58.7 (s, CH_2), 53.9 (s, CH_2), 39.8 (s, CH_3).

Elemental analysis – calculated (%) for $C_{27}H_{39}N_7O_3$: C 63.63, H 7.71, N 19.24; found (%): C 63.64, H 7.79, N 19.28.

3. Synthesis of Copper Complexes

Synthesis of [Cu(tmpa-(OH)$_1$(CH$_3$CN)](OTf)$_2$ (Cu/TMPA-(OH)$_1$)

Cu(OTf)$_2$ (79.7 mg. 0.22 mmol), and TMPA-(OH)$_1$ (89.0 mg, 0.24 mmol) were dissolved in 60 mL of dry MeCN. The reaction mixture was stirred under N_2 flow at RT for 3 h, after which it was filtered, and a clear dark green filtrate was obtained. The solvent was evaporated under reduced pressure to obtain a dark-green oily product. The green oil was dissolved in a minimal amount of MeCN and Et_2O was added dropwise until the solution became cloudy. This solution was placed at −18°C and after 2 days more Et_2O was added after two days. After two more days, the solvent was removed and a green oil was obtained at the bottom of the flask. The oil was sonicated in Et_2O multiple times. Thereafter, the recrystallization was repeated using MeOH instead of MeCN. The final oil was placed in a vacuum oven at 80 °C for three days to obtain pure [Cu(tmpa-(OH)$_1$(CH$_3$CN)](OTf)$_2$.

Elemental analysis: calculated (%) for $C_{25}H_{28}CuF_6N_6O_7S_2$: C 39.19, H 3.68, N 10.97; found (%): C 38.87, H 3.61, N 10.91.

LC-MS (m/z) – 182.8 [tmpa-(OH)$_1$ + 2H$^+$]$^{2+}$, 233.8 [Cu(tmpa-(OH)$_1$(CH3CN)]$^{2+}$, 364.3 [tmpa-(OH)$_1$ + H$^+$]$^+$, 426.2 [Cu(tmpa-(OH)$_1$]$_2^{2+}$, 575.2 [(Cu(tmpa-(OH)$_1$)$_2$ + 2OTf$^-$]$^{2+}$

Synthesis of [Cu(tmpa-(OH)$_2$(CH$_3$CN)](OTf)$_2$ (Cu/TMPA-(OH)$_2$)

Cu(OTf)$_2$ (40.4 mg. 0.11 mmol) and tmpa-(OH)$_2$ (60.9 mg, 0.14 mmol) were each dissolved in 10 mL of dry CH_3CN. The two solutions were mixed and stirred for 2.5 h at RT under N_2 flow. Subsequently, the dark green reaction mixture was filtered, and a clear dark green filtrate was obtained. The solvent was evaporated under reduced pressure to obtain a dark-green oily product. The green product was dissolved in a minimal amount of CH_3CN and Et_2O was added dropwise until the solution became cloudy. Thereafter, the solution was placed at −18°C and after 2 days more Et_2O was added after two days. After two more days, a green oil was obtained at the bottom of the flask. This recrystallization was repeated one more time. The liquid was removed and the product was placed in a vacuum oven at 80 °C. After drying for 2 d under vacuum, [Cu(tmpa-(OH)$_2$(CH$_3$CN)](OTf)$_2$ was obtained as a green solid (yield: 86.2 mg, 93 %).

Elemental analysis: calculated (%) for $C_{28}H_{35}CuF_6N_7O_9S_2$: C 40.07, H 4.20, N 11.68; found (%): C 39.61, H 4.23, N 11.54.

LC-MS (m/z) – 219.3 [tmpa-(OH)$_2$ + 2H$^+$]2+, 271.1 [Cu(tmpa-(OH)$_2$ (CH$_3$CN)]$^{2+}$, 437.3 [tmpa-(OH)$_2$ + H$^+$]$^+$, 648.2 [(Cu(tmpa--(OH)$_2$)$_2$ + 2OTf$^-$]$^{2+}$

Synthesis of [Cu(tmpa-$(OH)_3$(CH_3OH)]$(OTf)_2$ (Cu/TMPA-$(OH)_3$)

Cu$(OTf)_2$ (12.2 mg, 0.03 mmol) and TMPA-$(OH)_3$ (21.5 mg. 0.04 mmol) were separately dissolved in 10 mL of dry CH_3CN. The two solutions were mixed and stirred overnight at RT under a N_2 flow. Subsequently, the dark green reaction mixture was filtered under vacuum, and a clear dark green filtrate was obtained. The solvent was evaporated under reduced pressure to obtain a dark-green oily product. The green product was dissolved in a minimal amount of CH_3CN and Et2O was added dropwise until the solution became cloudy. The solution was placed at −18°C and after 2 days more Et_2O was added after two days. After two more days, the liquid was removed and a dark green oil was formed at the bottom of the flask. The recrystallization was repeated using MeOH instead of MeCN. Finally, the product was heated in a vacuum oven at 80 °C. After drying for 2 d under vacuum, [Cu(tmpa-$(OH)_3$(MeOH)]$(OTf)_2$ (Cu(tmpa-$(OH)_3$)) was obtained as a green solid (yield: 21.9 mg, 71 %).

Elemental analysis: calculated (%) for $C_{30}H_{43}CuF_6N_7O_{10}S_2$: C 39.89, H 4.80, N 10.85; determined (%): C 39.16, H 4.51, N 10.72.

LC-MS (m/z) – 255.9 [tmpa-$(OH)_3$ + $2H^+$]$^{2+}$, 306.7 [Cu(tmpa-$(OH)_3$)(MeOH)]$^{2+}$, 510.3 [(tmpa-$(OH)_3$) + H^+]$^+$, 721.2 [(Cu(tmpa-$(OH)_3$))$_2$ + $2OTf^-$]$^{2+}$

4. Overview of all Redox Properties

Table S1 Electrochemical parameters of the Cu complexes obtained from cyclic voltammetry.

Catalyst	Electrolyte	E_{red} (mV)	E_{ox} (mV)	ΔE_p (mV)	$E_{1/2}$ (mV)	D (cm²/s)[b]
**Cu/TMPA**	0.1 M PB[a]	-231	-179	52	-205	4.90×10^{-6} [d]
	0.1 M NaBr[a]	-230	-142	88	-186	/
	0.1 M SP + 0.01 M PB[b]	-200	-113	87	-157	/
	0.1 M SP + 4 mM NaBr + 10 vol% $OEOA_{480}$[b]	-101	71	172	-15	/
	0.1 M SP + 0.1 NaBr + 10 vol% $OEOMA_{500}$[b]	-87	-25	62	-56	/
**Cu/TMPA-(OH)$_1$**	PB[a]	-223	-160	63	-192	4.89×10^{-6}
	NaBr[a]	-262	-179	83	-221	4.94×10^{-6}
	0.1 M SP + 0.01 M PB[b]	-221	-132	89	-177	n.d.
	0.1 M SP + 4 mM NaBr + 10 vol% $OEOA_{480}$[b]	-126	69	195	-29	n.d.
	0.1 M SP + 0.1 M NaBr + 10 vol% $OEOMA_{500}$[b]	-142	-44	98	-93	n.d.
**Cu/TMPA-(OH)$_2$**	PB[a]	-267	-123	144	-195	5.47×10^{-6}
	NaBr[a]	-279	-193	86	-236	6.30×10^{-6}
	0.1 M SP + 0.01 M PB[b]	-275	-169	106	-222	n.d.
	0.1 M SP + 4 mM NaBr + 10 vol% $OEOA_{480}$[b]	-177	89	>250	-44	n.d.
	0.1 M SP + 0.1 NaBr + 10 vol% $OEOMA_{500}$[b]	-174	-77	95	-126	n.d.
**Cu/TMPA-(OH)$_3$**	PB[a]	-265	-116	149	-191	[e]5.18×10^{-6}
	NaBr[a]	-281	-196	85	-239	6.62×10^{-6}
	0.1 M SP + 0.01 M PB[b]	-377	-122		-250	n.d.
	0.1 M SP + 4 mM NaBr + 10 vol% $OEOA_{480}$[b]	-224	/[f]	/[f]	/[f]	n.d.

[a]CVs recorded on a GC disk electrode at a scan rate of 100 mV/s at RT. [b.]CVs recorded on a GC disk electrode at a scan rate of 200 mV/s at 35 °C using an SCE reference electrode. The potential values were then converted to the NHE using $E_{NHE} = E_{SCE} + 0.233$ V. Numbers in brackets in the case of Cu/TMPA-(OH)$_3$ correspond to E_{pc} values. [d.]Obtained from Ref [7]. [e.]Calculated with high uncertainty owing to irreproducibility of the redox couple. [f.]Irreversible cyclic voltammetry.

5. Scan Rate Dependence Measurements

The Cu^{II}/Cu^{I} redox couples of Cu/TMPA-$(OH)_1$, Cu/TMPA-$(OH)_2$, and Cu/TMPA-$(OH)_3$ were measured in CV experiments with varying scan rates between 10 mV/s to 500 mV/s in a randomized order (See **Figure S1a**, **Figure S2a**, **Figure S3a**). From these measurements, the diffusion coefficients of all species were calculated using the Randles-Sevcik plot (**Figure S1b**, **Figure S2b**, **Figure S3b**) and **Equation 1**, where i_p is the peak current of the redox couple, n is the number of electrons transferred (n = 1), T is the temperature (T = 298 K), A is the surface of the electrode (A = 0.071 cm^2), C^0 is the concentration of catalyst in the bulk (C^0 = 0.3 mM), and v is the scan rate. The derived diffusion coefficients can be found in **Figure S1b**, **Figure S2b**, **Figure S3b** and **Table 1** of the main text.

$$i_p = 0.446 nFAC^0 \left(\frac{nFvD}{RT}\right)^{\frac{1}{2}} \qquad \textbf{(1)}$$

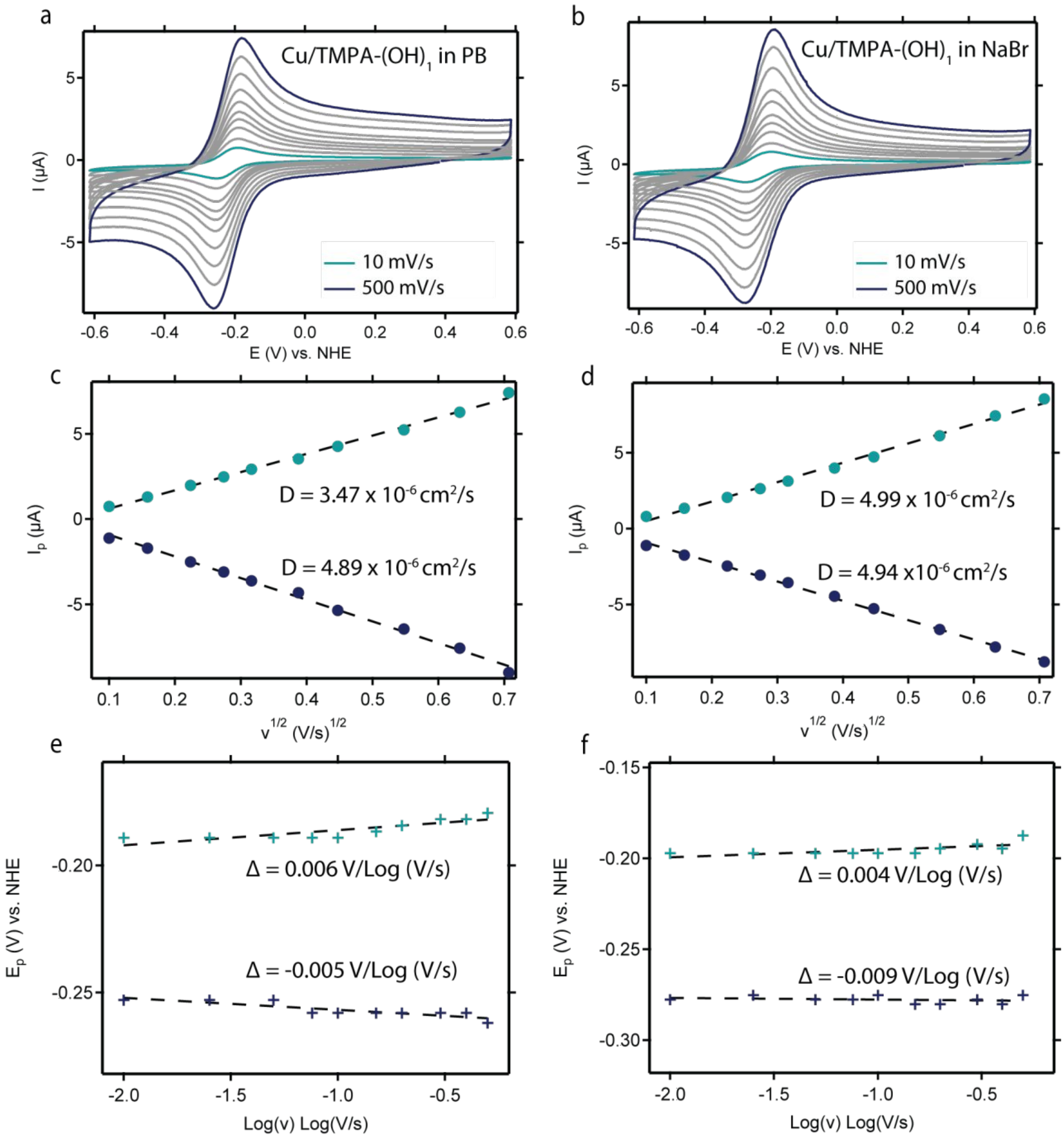

Figure S1 Scan rate dependence of the Cu/TMPA-$(OH)_1$ redox couple at varying scan rates between 10 mV/s (light blue) and 500 mV/s (dark blue) in PB (**a**) and NaBr (**b**). Randles-Sevcik plot of the anodic (light blue, R^2 = 0.99) and cathodic (dark blue, R^2 = 0.99) peak currents of Cu/TMPA-$(OH)_1$ as a function of the square root of the scan rate in PB (**c**) and NaBr (**d**). Laviron plot of the anodic (light blue) and cathodic (dark blue) peak potentials of Cu/TMPA-$(OH)_1$ redox couple in PB (**e**) and NaBr (**f**). Conditions: 0.1 M NaBr and PB pH 7, Ar atmosphere, 293 K, 0.3 mM Cu(TMPA-$(OH)_1$).

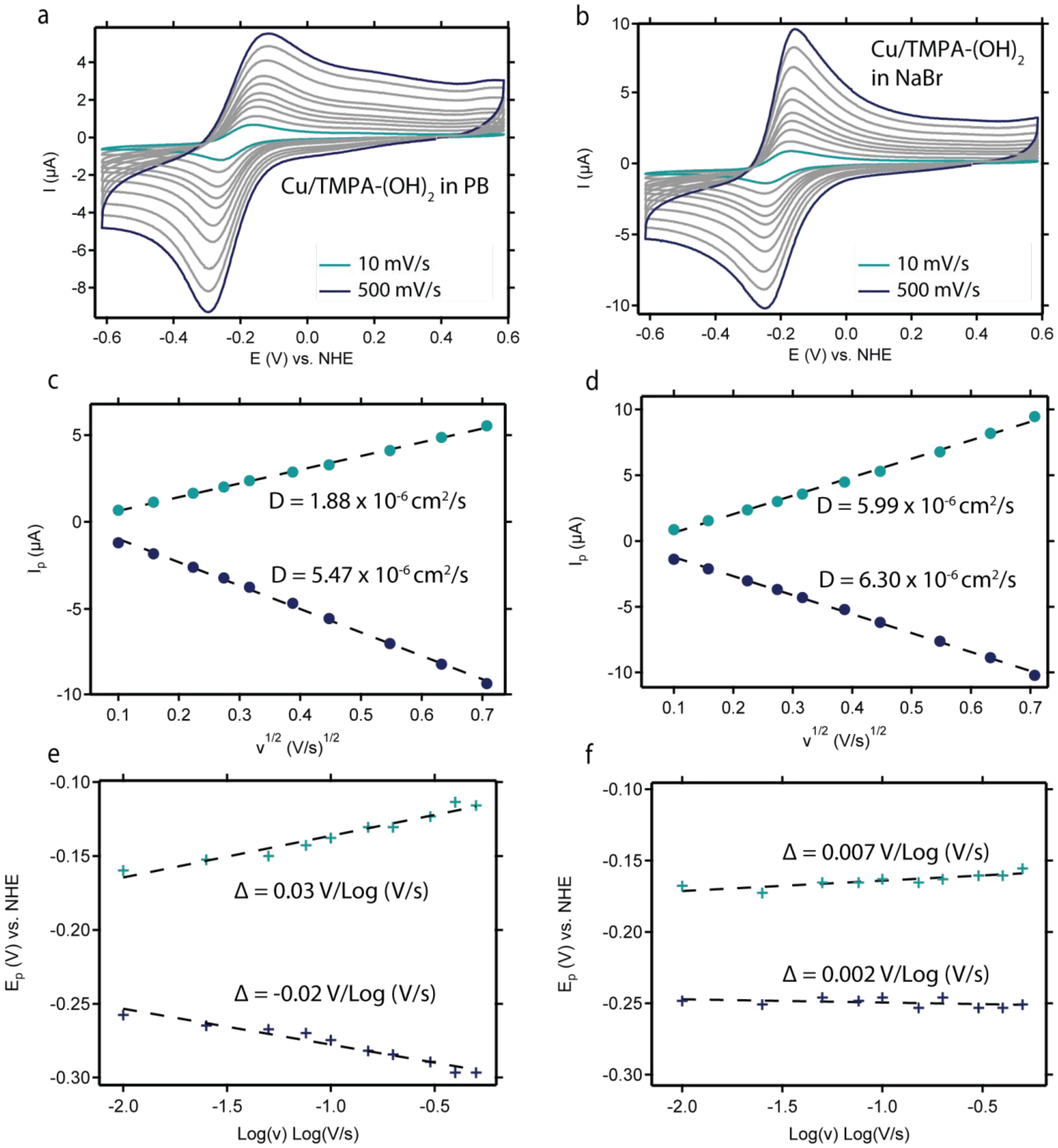

Figure S2 Scan rate dependence of the Cu/TMPA-$(OH)_2$ redox couple at varying scan rates between 10 mV/s (light blue) and 500 mV/s (dark blue) in PB (**a**) and NaBr (**b**). Randles-Sevcik plot of the anodic (light blue, R^2 = 0.99) and cathodic (dark blue, R^2 = 0.99) peak currents of Cu/TMPA-$(OH)_2$ as a function of the square root of the scan rate in PB (**c**) and NaBr (**d**). Laviron plot of the anodic (light blue) and cathodic (dark blue) peak potentials of Cu/TMPA-$(OH)_2$ redox couple in PB (**e**) and NaBr (**f**). Conditions: 0.1 M NaBr and PB pH 7, Ar atmosphere, 293 K, 0.3 mM Cu/TMPA-$(OH)_2$.

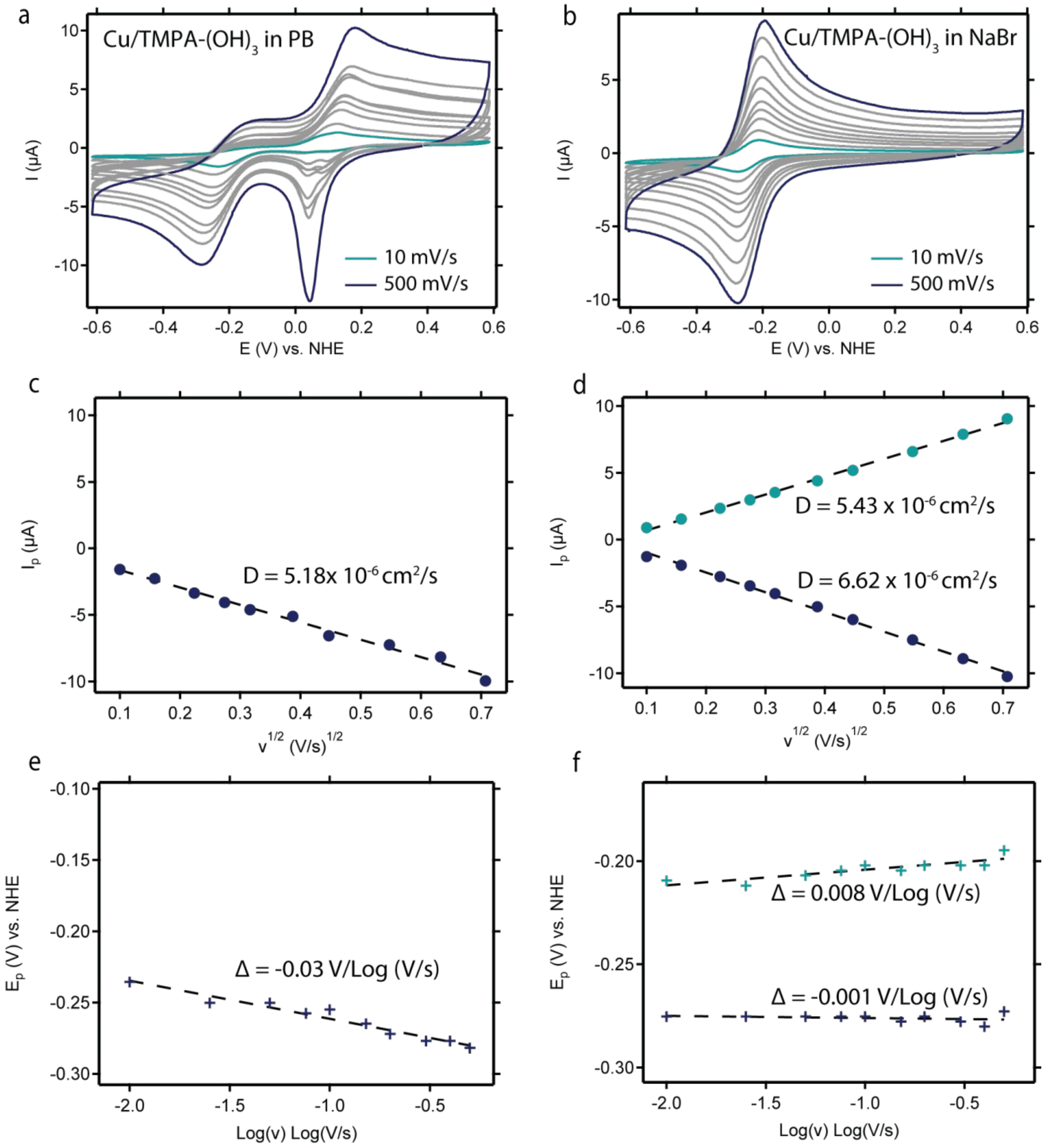

Figure S3 Scan rate dependence of the Cu/TMPA-$(OH)_3$ redox couple at varying scan rates between 10 mV/s (light blue) and 500 mV/s (dark blue) in PB (**a**) and NaBr (**b**). Randles-Sevcik plot of the anodic (light blue, R^2 = 0.99) and cathodic (dark blue, R^2 = 0.99) peak currents of Cu/TMPA-$(OH)_3$ as a function of the square root of the scan rate in PB (**c**) and NaBr (**d**). Laviron plot of the anodic (light blue) and cathodic (dark blue) peak potentials of Cu/TMPA-$(OH)_3$ redox couple in PB (**e**) and NaBr (**f**). Conditions: 0.1 M NaBr and PB pH 7, Ar atmosphere, 293 K, 0.3 mM Cu/TMPA-$(OH)_3$. The oxidation peak in PB was left out of the analysis.

6. CV Measurements of Cu/TMPA-$(OH)_3$

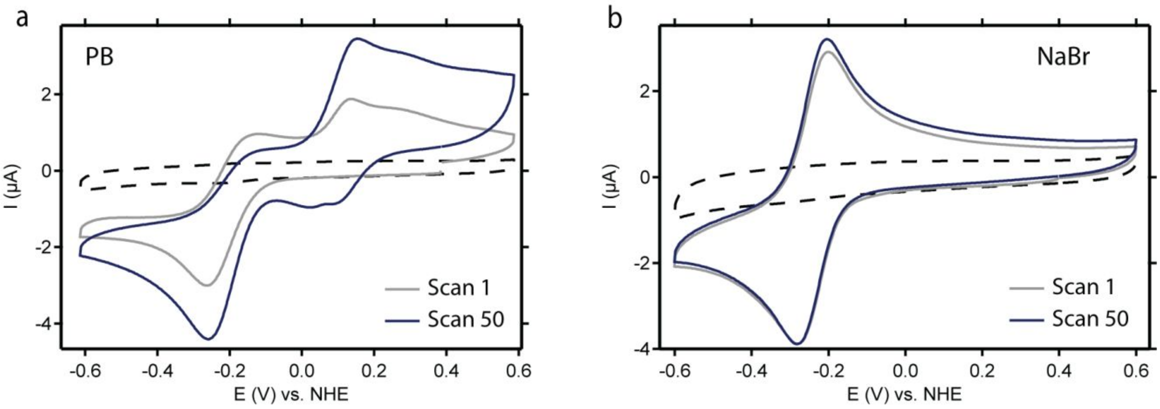

Figure S4 50 consecutive CV scans of Cu/TMPA-$(OH)_3$ recorded in a) PB and b) NaBr electrolytes. Conditions: Ar atmosphere, 100 mV/s scan rate, 0.3 mM Cu/TPMA-$(OH)_3$, 0.1 M PB and 0.1 M NaBr of pH 7.

7. CV Measurements in Presence of both PB and NaBr

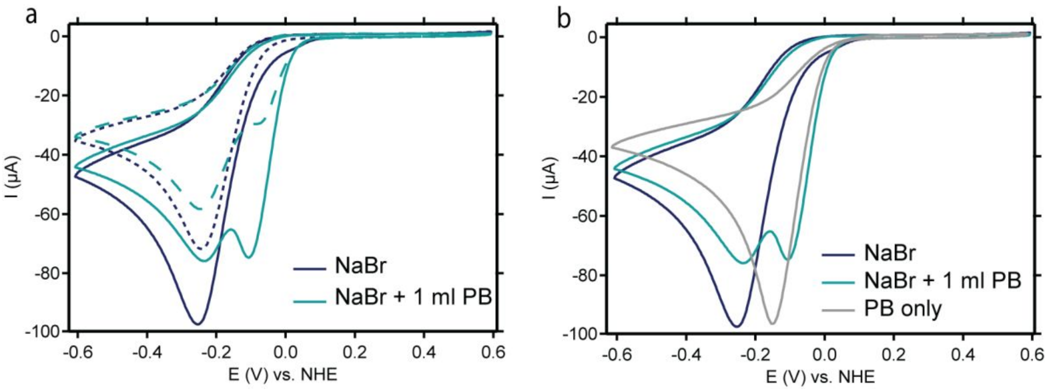

Figure S5 a) CV measurements of Cu/TMPA in NaBr (dark blue) and NaBr + 1ml PB (light blue), comparing scan 1 (solid line) and scan 2 (dotted line). And **b**) CV measurement of Cu/TMPA in NaBr (dark blue), NaBr + 1 ml PB (light blue) compared to measurements in PB only (grey). Conditions: 1 atm O_2, 100 mV/s scan rate, 0.3 mM Cu/TMPA, 0.1 M PB pH 7, 0.1 M NaBr pH 7.

8. Foot-of-the-wave Analysis

Foot-of-the-wave analysis (FOWA) was carried out to obtain the maximum turnover frequency (TOF_{max}) of the ORR and HPRR for every catalyst in both PB and NaBr electrolyte. The graphs relevant for the FOWA of Cu/TMPA-$(OH)_1$ are shown in **Figure S**, **Figure S**, **Figure S6**, and **Figure S10**, for Cu/TMPA-$(OH)_2$ in **Figure S**, **Figure S**, **Figure S7**, and **Figure S11**, and for Cu/TMPA-$(OH)_3$ in **Figure S**, **Figure S5**, and **Figure S8**. For Cu/TMPA, the TOF_{max} values for the ORR and HPRR in PB have been reported in previous studies,[3, 8] and the measurements in NaBr are shown in **Figure S9** and **Figure S12**. It was established that for Cu/TMPA, **Equation 2** can be used to extract the TOF_{max}.[3] This equation is applicable when the potential determining step of the catalytic cycle is the reduction of the Cu(II) center and the rate-determining step is the binding of O_2, resulting in an EC' type mechanism.[9] Herein, we have assumed that the *p*-substituted TMPA-derivatives will follow a catalytic mechanism similar to Cu/TMPA, and have thus used the same FOWA equations.

$$\frac{i_c}{i_p} = \frac{2.24 n_{cat}\sqrt{\frac{RT}{Fv} TOF_{max}}}{1 + exp\left[\frac{F}{RT}\left(E - E_{1/2}\right)\right]} \qquad \textbf{(2)}$$

In **Equation 2**, i_c is the catalytic current measured in the presence of the catalyst and substrate at a certain potential *E*, and i_p is the peak current of the reduction of the Cu(II) center in the absence of the substrate. In all the cases, the TOF_{max} values were determined based on a minimum of two catalytic CVs in the presence of O_2 or H_2O_2. Thereafter, plots of i_c/i_p vs. 1/(1+exp((E-$E_{1/2}$)F) were used to derive the TOF_{max} from the slope of a linear fit in the region where $i_c/i_{redox} \geq 2$, and the R^2 of the linear fit was ≥ 0.99. In this case, i_{redox} is the current measured in the presence of a catalyst but in the absence of a substrate. All calculated TOF_{max} values are listed in **Table S2-4**.

8.1 ORR Measurements in PB

Table S2. TOF_{max} values of ORR determined for Cu/TMPA-$(OH)_1$, Cu/TMPA-$(OH)_2$, and Cu/TMPA-$(OH)_3$ from CV measurements recorded in PB at pH 7 under 1 atm O_2.

Catalyst	#1	#2	#3	Average
Cu(TMPA)				$^{a}1.8 \times 10^6 \pm 0.6\times10^6$
Cu/TMPA-$(OH)_1$	1.28×10^5	8.03×10^4	5.08×10^4	$8.64\times10^4 \pm 1.8\times10^4$
Cu/TMPA-$(OH)_2$	2.61×10^3	4.35×10^3	2.96×10^3	$3.31\times10^3 \pm 0.4\times10^3$
Cu/TMPA-$(OH)_3$	2.27×10^4	7.37×10^3	3.42×10^4	$2.14\times10^4 \pm 0.6\times10^3$

[a.] *Obtained from Ref* [7].

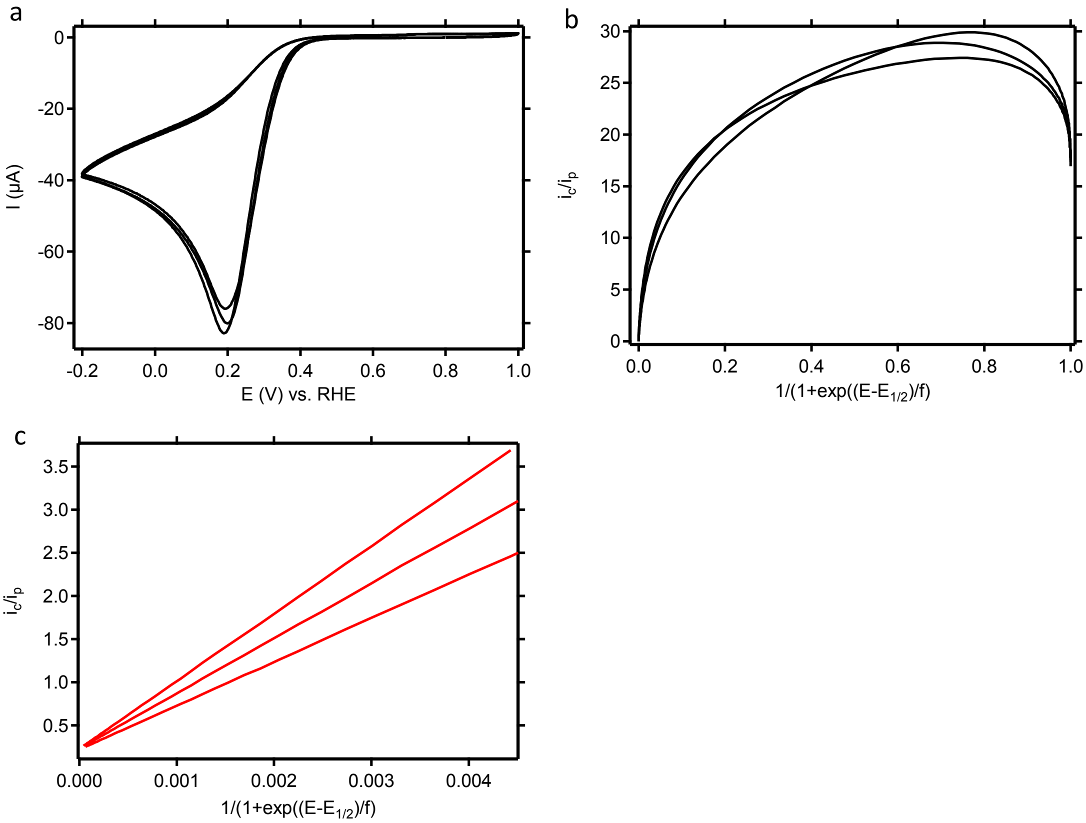

Figure S6 a) ORR CVs recorded in presence of 0.3 mM Cu/TMPA-$(OH)_1$, **b**) corresponding FOWA graphs of the ORR, where f = F/RT, and **c**) fits of the linear regions of the FOWA graphs, for which $R^2 \geq 0.99$. Conditions: pH 7 0.1 M PB, 293 K, 100 mV s^{-1} scan rate, 1 atm O_2.

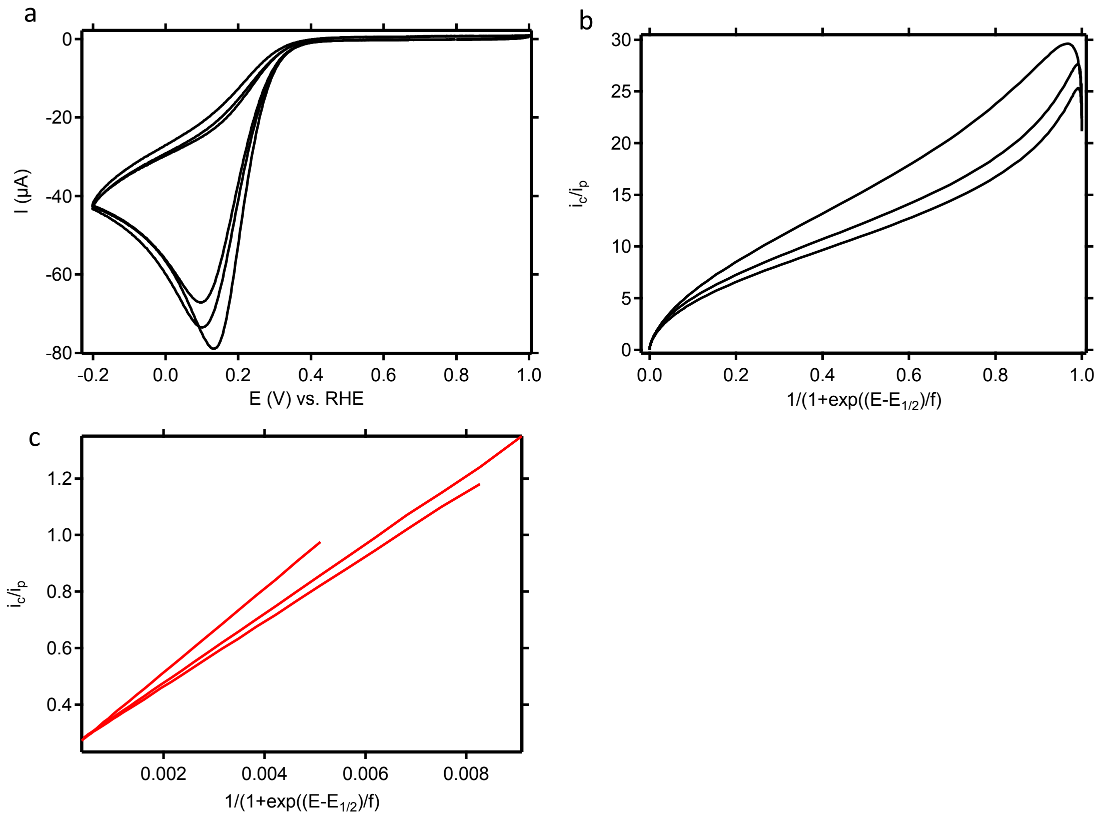

Figure S7 a) ORR CVs recorded in presence of 0.3 mM Cu/TMPA-$(OH)_2$, **b**) corresponding FOWA graphs of the ORR, where f = F/RT, and **c**) fits of the linear regions of the FOWA graphs, for which $R^2 \geq 0.99$. Conditions: pH 7 0.1 M PB, 293 K, 100 mV s^{-1} scan rate, 1 atm O_2.

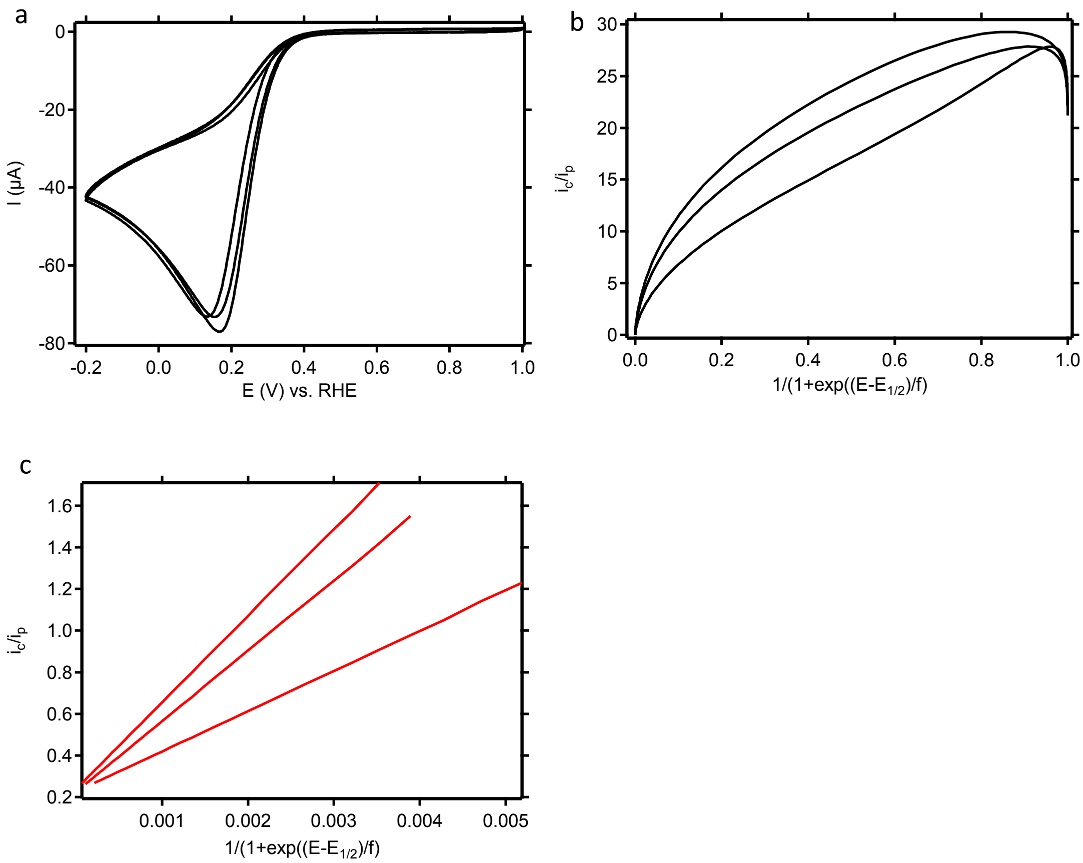

Figure S8 a) ORR CVs recorded in presence of 0.3 mM Cu/TMPA-$(OH)_3$, **b**) corresponding FOWA graphs of the ORR, where f = F/RT, and **c**) fits of the linear regions of the FOWA graphs, for which $R^2 \geq 0.99$. Conditions: pH 7 0.1 M PB, 293 K, 100 mV s^{-1} scan rate, 1 atm O_2.

HPRR Measurements in PB

Table S3 TOF_{max} values of HPRR determined for Cu/TMPA-$(OH)_1$, Cu/TMPA-$(OH)_2$, and Cu/TMPA-$(OH)_3$ from CV measurements recorded in PB at pH 7 in the presence of 1.1 mM H_2O_2.

Catalyst	#1	#2	#3	Average
Cu/TMPA				$^{a}2.1\times10^5 \pm 0.1\times10^5$
Cu/TMPA-$(OH)_1$	6.06×10^2	2.24×10^2	1.87×10^2	$3.39\times10^2 \pm 1.1\times10^2$
Cu/TMPA-$(OH)_2$	9.30×10^1	5.73×10^1	1.13×10^1	$5.39\times10^1 \pm 1.9\times10^1$
Cu/TMPA-$(OH)_3$	6.15×10^1	2.18×10^2	3.06×10^1	$1.03\times10^2 \pm 0.6\times10^2$

[a.] Obtained from Ref [7].

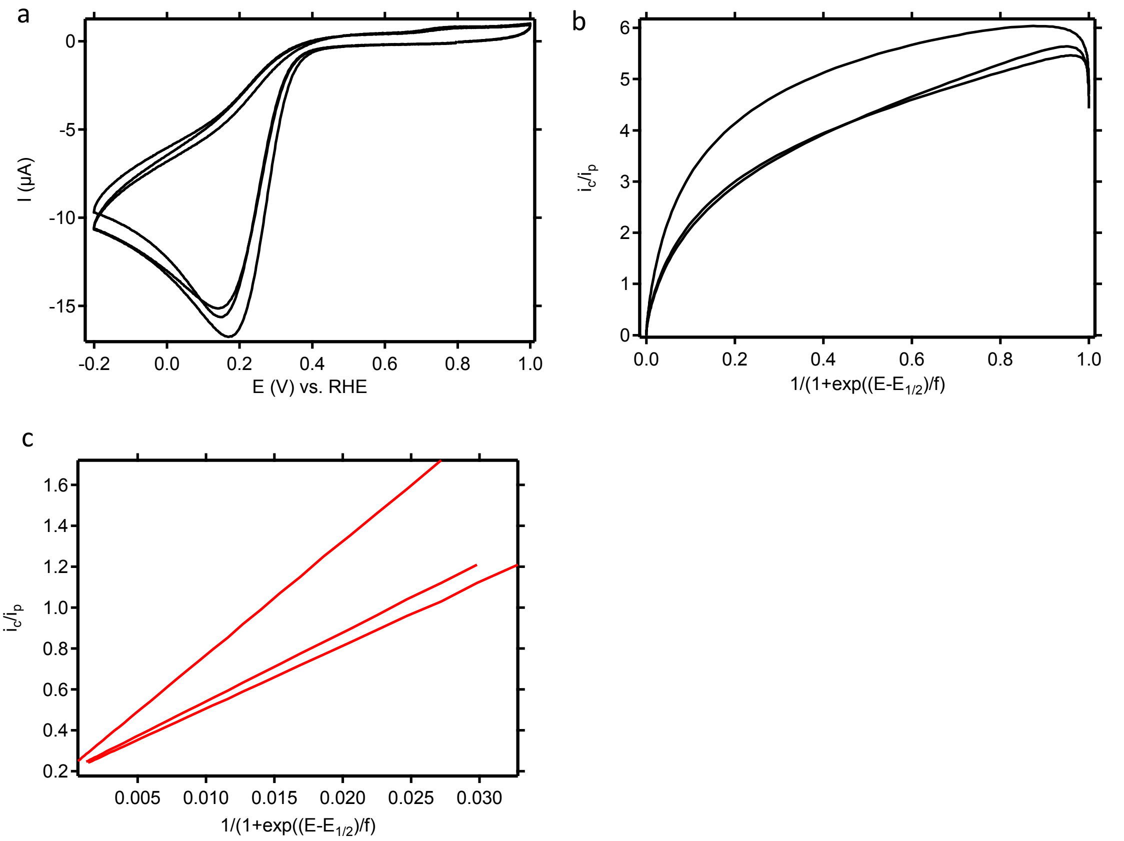

Figure S9 a) HPRR CVs recorded in presence of 0.3 mM Cu/TMPA-$(OH)_1$, **b**) corresponding FOWA graphs of the HPRR, where f = F/RT, and **c**) fits of the linear regions of the FOWA graphs, for which $R^2 \geq 0.99$. Conditions: pH 7 0.1 M PB, 293 K, 100 mV s^{-1} scan rate, 1.1 mM H_2O_2.

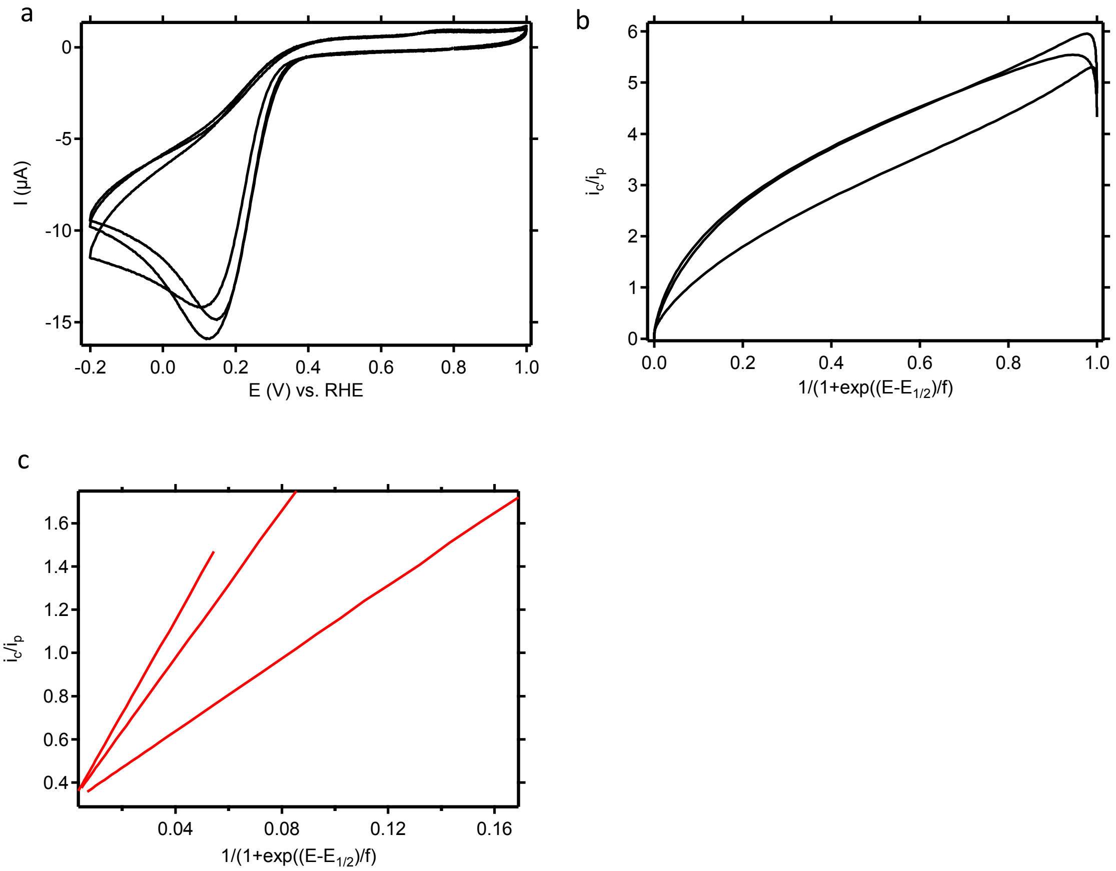

Figure S10 a) HPRR CVs recorded in presence of 0.3 mM Cu/TMPA-$(OH)_2$, **b**) corresponding FOWA graphs of the HPRR, where f = F/RT, and **c**) fits of the linear regions of the FOWA graphs, for which $R^2 \geq 0.99$. Conditions: pH 7 0.1 M PB, 293 K, 100 mV s^{-1} scan rate, 1.1 mM H_2O_2.

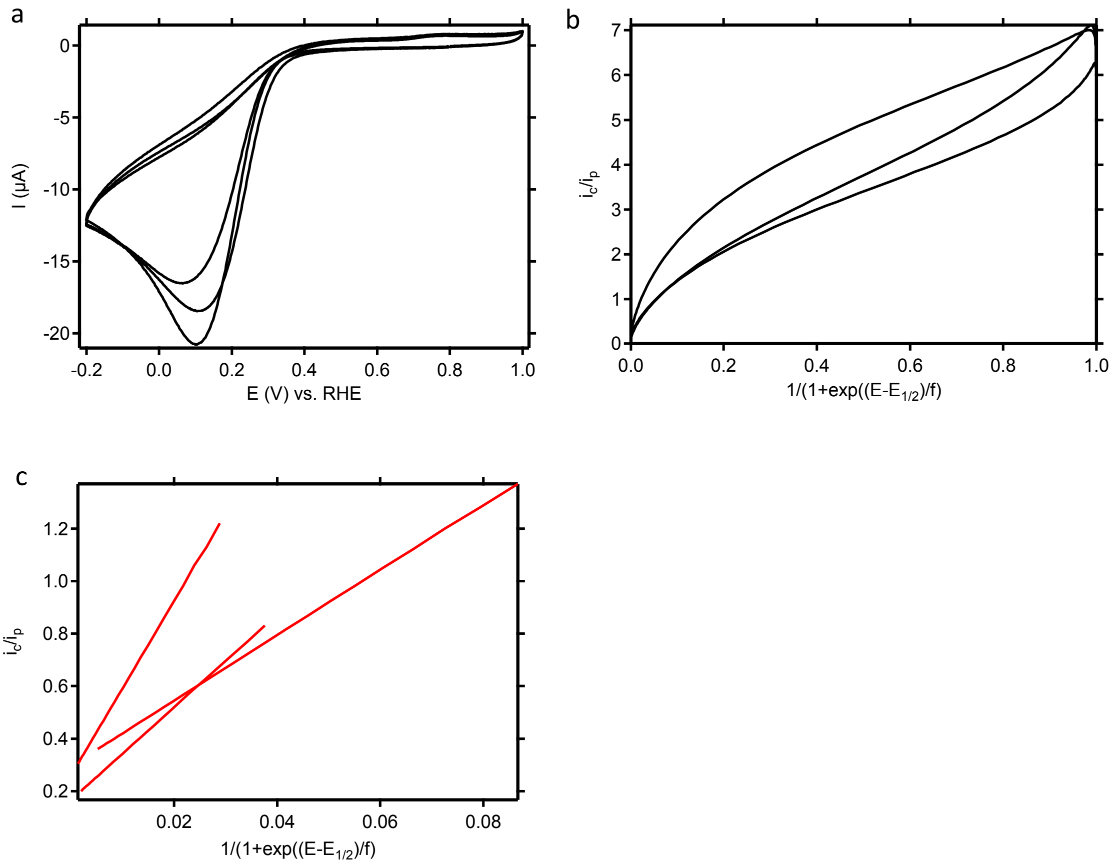

Figure S5 a) HPRR CVs recorded in presence of 0.3 mM Cu/TMPA-OH)$_3$, **b**) corresponding FOWA graphs of the HPRR, where f = F/RT, and **c**) fits of the linear regions of the FOWA graphs, for which $R^2 \geq 0.99$. Conditions: pH 7 0.1 M PB, 293 K, 100 mV s^{-1} scan rate, 1.1 mM H_2O_2.

ORR Measurements in NaBr

Table S4 TOF_{max} values of the ORR determined for Cu/TMPA-$(OH)_1$, Cu/TMPA-$(OH)_2$, and Cu/TMPA-$(OH)_3$ from CV measurements recorded in 0.1 M NaBr under 1 atm O_2.

Catalyst	#1	#2	#3	Average
Cu/TMPA	1.55×10^6	9.26×10^5		$1.24\times10^6 \pm 0.3\times10^6$
Cu/TMPA-$(OH)_1$	7.50×10^4	8.85×10^4	1.85×10^4	$6.07\times10^4 \pm 2.1\times10^4$
Cu/TMPA-$(OH)_2$	8.40×10^4	8.90×10^4	1.06×10^5	$9.30\times10^4 \pm 0.2\times10^4$
Cu/TMPA-$(OH)_3$	3.67×10^5	9.68×10^5		$6.67\times10^5 \pm 2.1\times10^5$

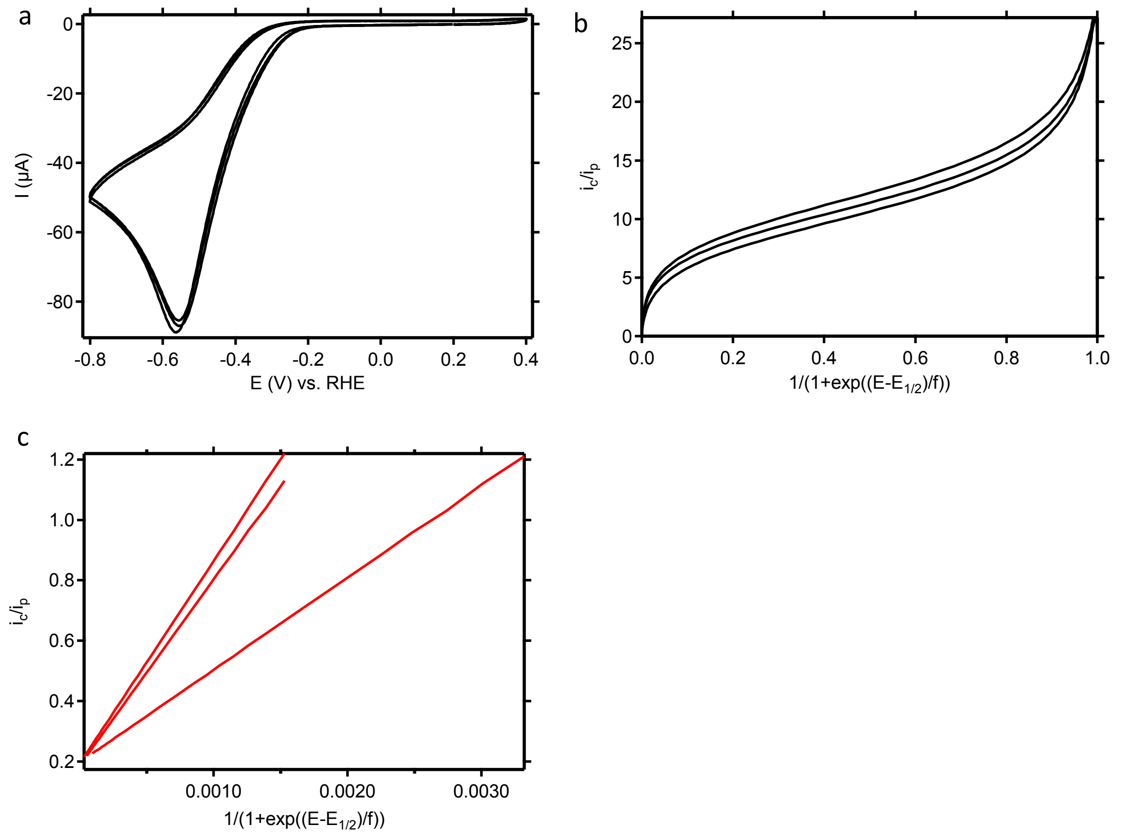

Figure S6 a) ORR CVs recorded in presence of 0.3 mM Cu/TMPA-$(OH)_1$, **b**) corresponding FOWA graphs of the ORR, where f = F/RT, and **c**) fits of the linear regions of the FOWA graphs, for which $R^2 \geq 0.99$. Conditions: 0.1 M NaBr, 293 K, 100 mV s^{-1} scan rate, 1 atm O_2.

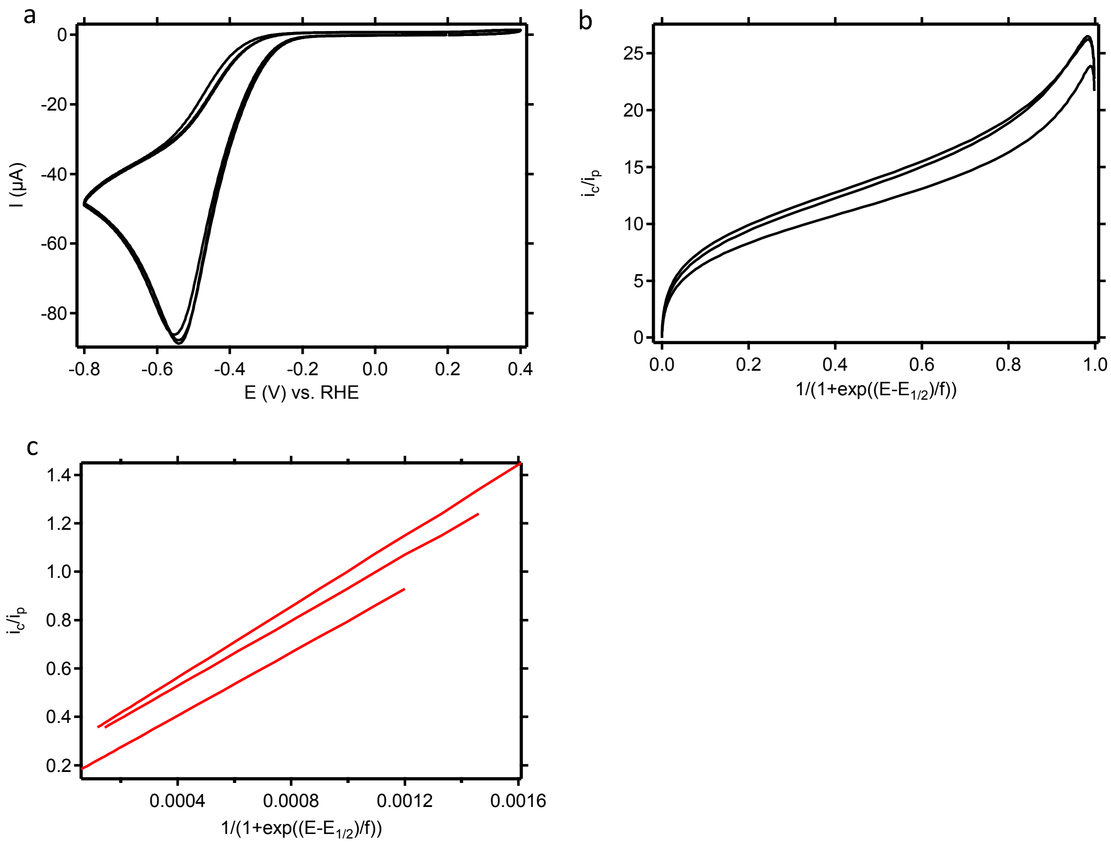

Figure S7 a) ORR CVs recorded in presence of 0.3 mM Cu/TMPA-$(OH)_2$, **b**) corresponding FOWA graphs of the ORR, where f = F/RT, and **c**) fits of the linear regions of the FOWA graphs, for which $R^2 \geq 0.99$. Conditions: 0.1 M NaBr, 293 K, 100 mV s^{-1} scan rate, 1 atm O_2.

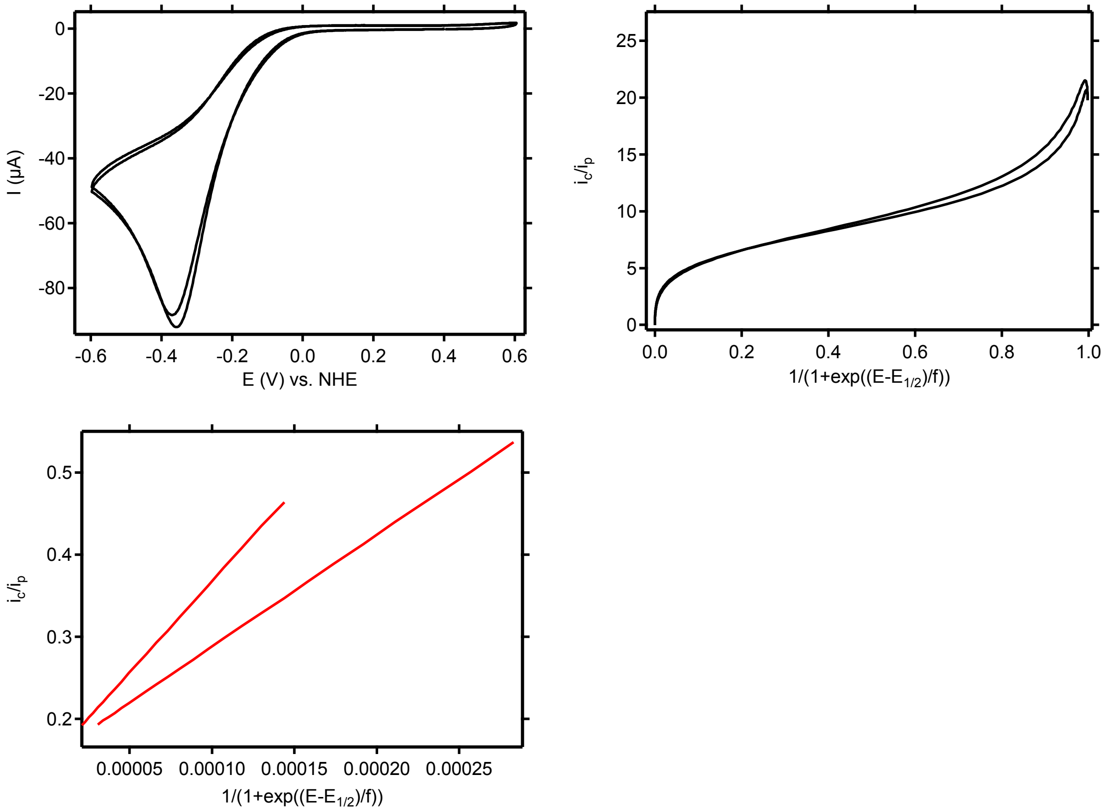

Figure S8 a) ORR CVs recorded in presence of 0.3 mM Cu/TMPA-$(OH)_3$, **b**) corresponding FOWA graphs of the ORR, where f = F/RT, and c) fits of the linear regions of the FOWA graphs, for which $R^2 \geq 0.99$. Conditions: 0.1 M NaBr, 293 K, 100 mV s^{-1} scan rate, 1 atm O_2.

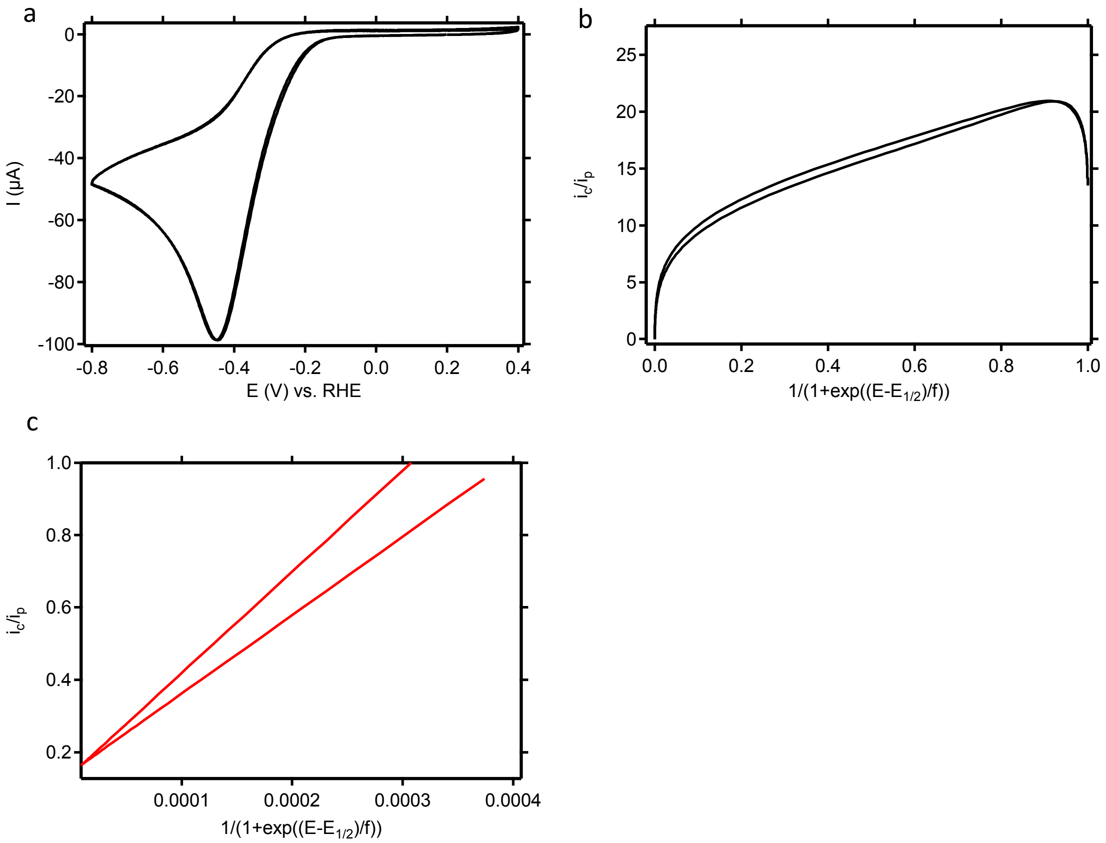

Figure S9 a) ORR CVs recorded in presence of 0.3 mM Cu/TMPA, **b**) corresponding FOWA graphs of the ORR, where f = F/RT, and **c**) fits of the linear regions of the FOWA graphs, for which $R^2 \geq 0.99$. Conditions: 0.1 M NaBr, 293 K, 100 mV s^{-1} scan rate, 1 atm O_2.

HPRR Measurements in NaBr

Table S5 TOF_{max} values of the HPRR determined for Cu/TMPA-$(OH)_1$, Cu/TMPA-$(OH)_2$, and Cu/TMPA-$(OH)_3$ from CV measurements recorded in 0.1 M NaBr in the presence of 1.1 mM H_2O_2.

Catalyst	#1	#2	#3	Average
Cu/TMPA	$2.84{\times}10^6$	$1.06{\times}10^6$	$1.60{\times}10^6$	$1.84{\times}10^6 \pm 0.4{\times}10^6$
Cu/TMPA-$(OH)_1$	$1.89{\times}10^3$	$3.10{\times}10^4$	$3.62{\times}10^3$	$1.73{\times}10^4 \pm 0.9{\times}10^4$
Cu/TMPA-$(OH)_2$	$7.30{\times}10^3$	$4.06{\times}10^4$		[a] $2.39{\times}10^4 \pm 1.2{\times}10^4$

[a.] *Average of two values.*

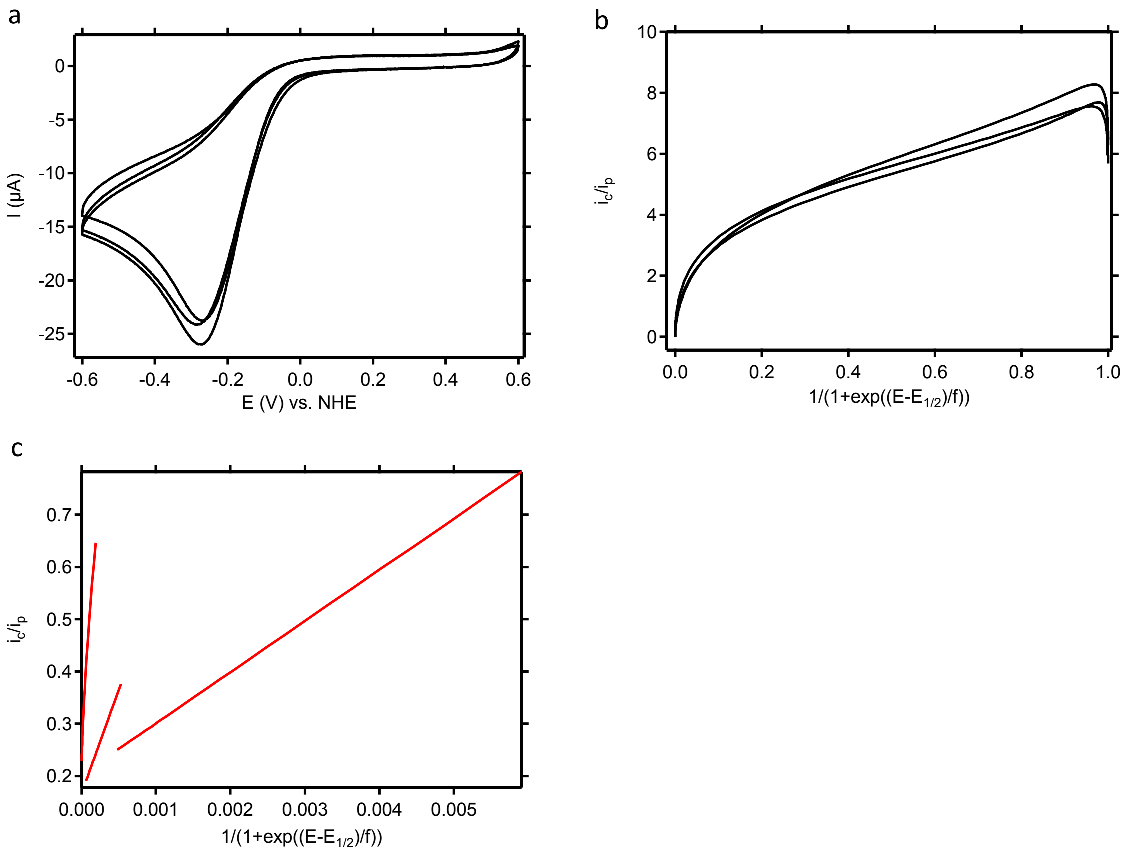

Figure S10 a) HPRR CVs recorded in presence of 0.3 mM Cu/TMPA-$(OH)_1$, **b**) corresponding FOWA graphs of the HPRR, where f = F/RT, and **c**) fits of the linear regions of the FOWA graphs, for which $R^2 \geq 0.99$. Conditions: 0.1 M NaBr, 293 K, 100 mV s^{-1} scan rate, 1.1 mM H_2O_2, Ar atmosphere.

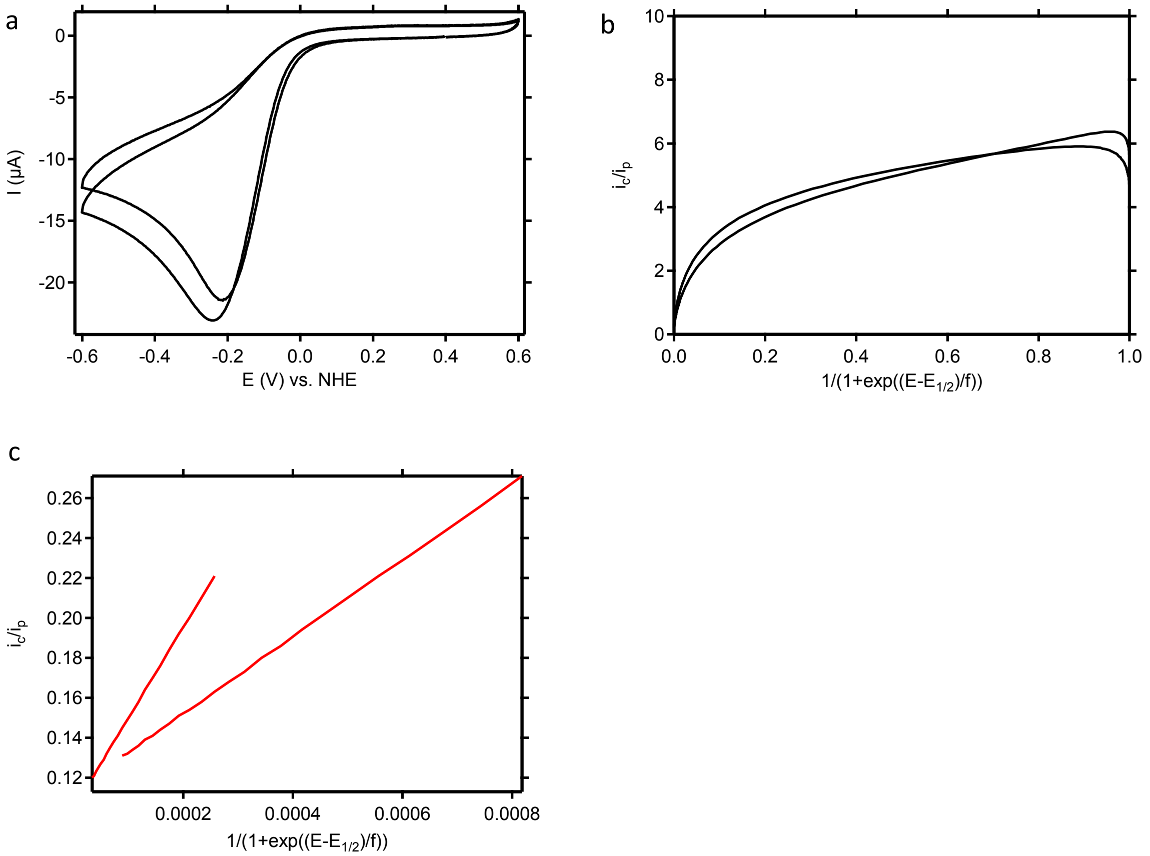

Figure S11 a) HPRR CVs recorded in presence of 0.3 mM Cu/TMPA-$(OH)_2$, **b**) corresponding FOWA graphs of the HPRR, where f = F/RT, and **c**) fits of the linear regions of the FOWA graphs, for which $R^2 \geq 0.99$. Conditions: 0.1 M NaBr, 293 K, 100 mV s^{-1} scan rate, 1.1 mM H_2O_2, Ar atmosphere.

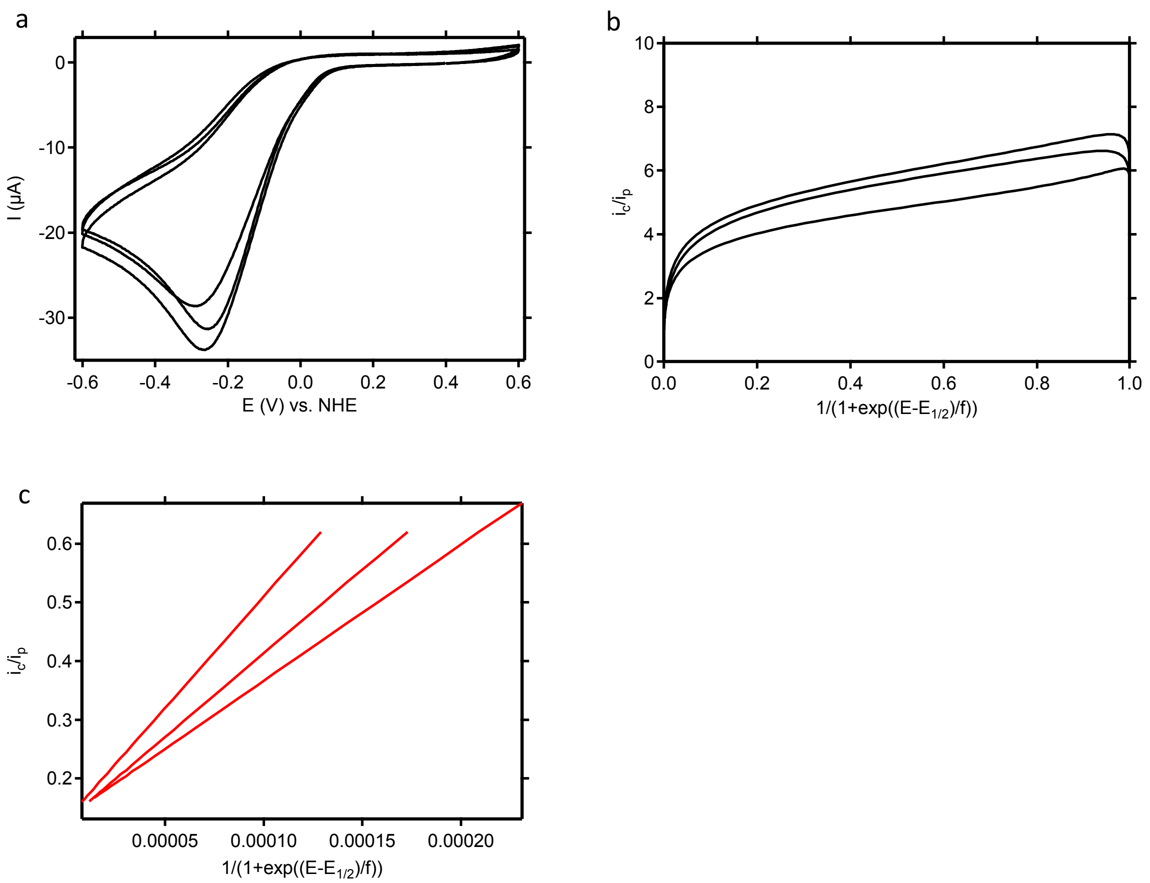

Figure S12 a) HPRR CVs recorded in presence of 0.3 mM Cu/TMPA, **b**) corresponding FOWA graphs of the HPRR, where f = F/RT, and **c**) fits of the linear regions of the FOWA graphs, for which $R^2 \geq 0.99$. Conditions: 0.1 M NaBr, 293 K, 100 mV s^{-1} scan rate, 1.1 mM H_2O_2, Ar atmosphere.

9. Rotating Ring-Disk Electrode Experiments

Prior to each scan, the collection efficiency of the setup (N) was determined to quantify the generated H_2O_2 during ORR catalysis (**Equation 3**), using the current at the disk (I_{disk}) and ring (I_{ring}). The percentage of H_2O_2 generated during the experiments was calculated using **Equation 4**.

$$N = \frac{I_{ring}}{I_{disk}} \quad \textbf{(3)}$$

$$\mathrm{H_2O_2}\ \% = \frac{2\frac{I_{ring}}{N}}{I_{disk} + \frac{I_{ring}}{N}}\ x\ 100\% \quad \textbf{(4)}$$

9.1 Koutecky-Levich Analysis

RRDE CVs of all para-substituted catalysts were recorded at varying rotation rates between 400 RPM and 2800 RPM, as shown in **Figure S13** to **Figure S14**. By plotting the inverse of the limiting current as a function of the square root of the rotation rate, Koutecky-Levich plots were obtained, which showed linearity over the complete range. This confirms that the plateau current in the ORR was independent of the rotation rate in all cases. Koutecky–Levich plots were not used to determine the electron transfer number of the ORR, as this is not suitable for this reaction.[10] Instead, the selectivity of the ORR was determined from RRDE measurements (see below).

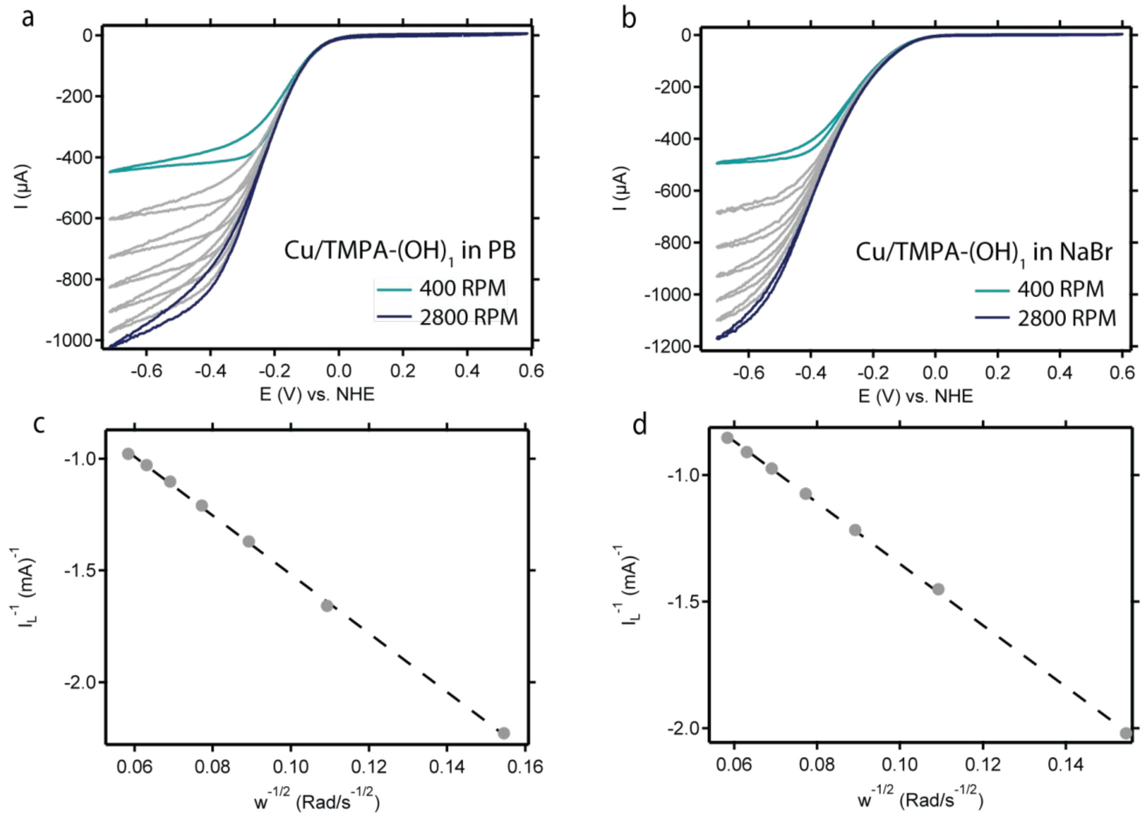

Figure S13 RDE CVs of Cu/TMPA-$(OH)_1$ under oxygen atmosphere with varying rotation rates between 400 RPM (light blue trace) and 2800 RPM (dark blue trace) measured in 0.1 M PB (**a**) and 0.1 M NaBr (**b**). Corresponding Koutecky-Levich plots derived from the catalytic plateau currents in 0.1 M PB (**c**) and 0.1 M NaBr (**d**). Conditions: 0.1 M PB pH 7 or 0.1 M NaBr, O_2 atmosphere, 293 K, 50 mV/s scan rate, 1600 RPM, 0.3 mM Cu/TMPA-$(OH)_1$.

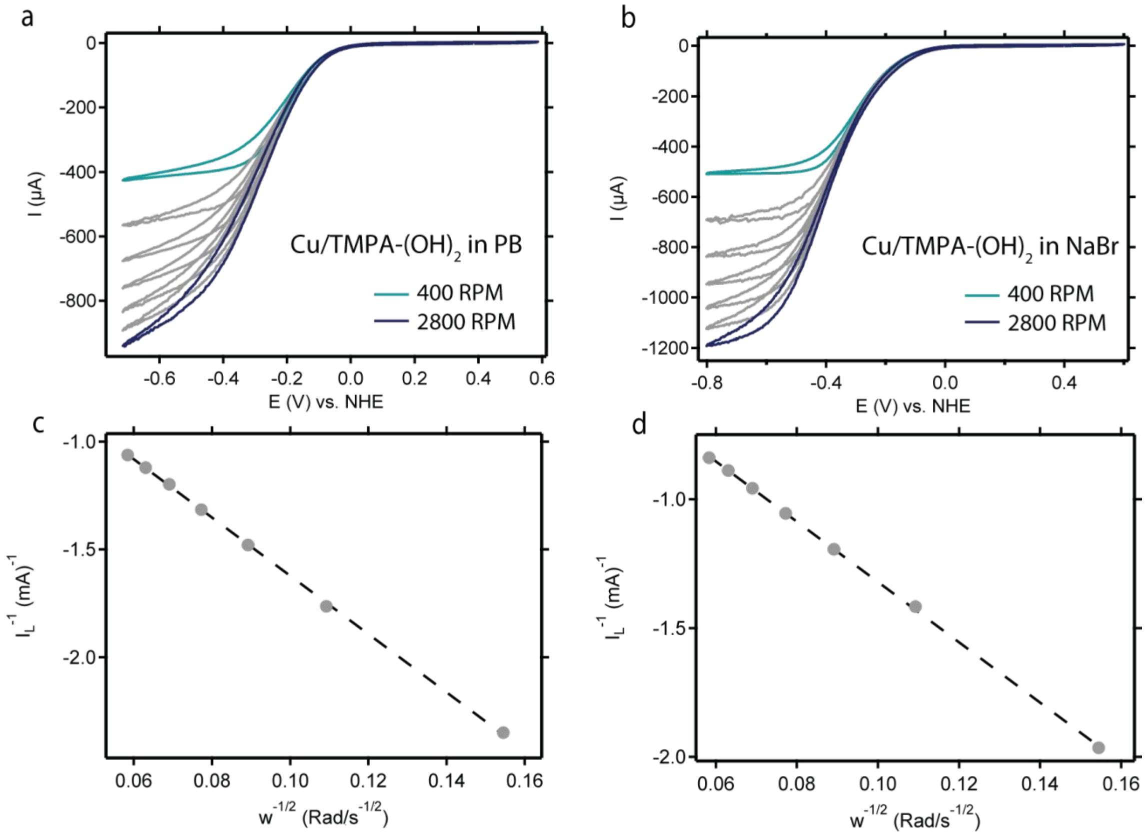

Figure S20 RDE CVs of Cu/TMPA-(OH)$_2$ under oxygen atmosphere with varying rotation rates between 400 RPM (light blue trace) and 2800 RPM (dark blue trace) measured in 0.1 M PB (**a**) and 0.1 M NaBr (**b**). Corresponding Koutecky-Levich plots derived from the catalytic plateau currents in 0.1 M PB (**c**) and 0.1 M NaBr (**d**). Conditions: 0.1 M PB pH 7 or 0.1 M NaBr, O_2 atmosphere, 293 K, 50 mV/s scan rate, 1600 RPM, 0.3 mM Cu/TMPA-(OH)$_2$.

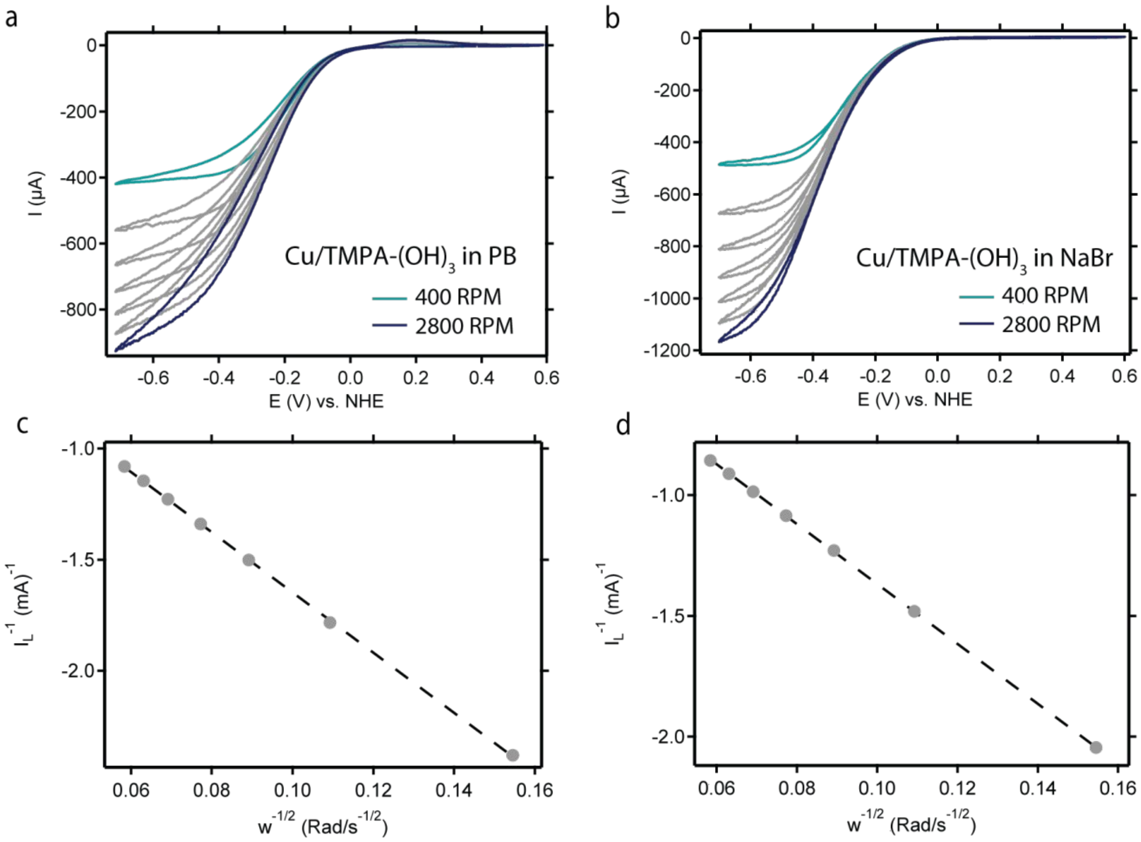

Figure S14 RDE CVs of Cu/TMPA-$(OH)_3$ under oxygen atmosphere with varying rotation rates between 400 RPM (light blue trace) and 2800 RPM (dark blue trace) measured in 0.1 M PB (**a**) and 0.1 M NaBr (**b**). Corresponding Koutecky-Levich plots derived from the catalytic plateau currents in 0.1 M PB (**c**) and 0.1 M NaBr (**d**). Conditions: 0.1 M PB pH 7 or 0.1 M NaBr, O_2 atmosphere, 293 K, 50 mV/s scan rate, 1600 RPM, 0.3 mM Cu/TMPA-$(OH)_3$.

9.2 RRDE Selectivity Measurements

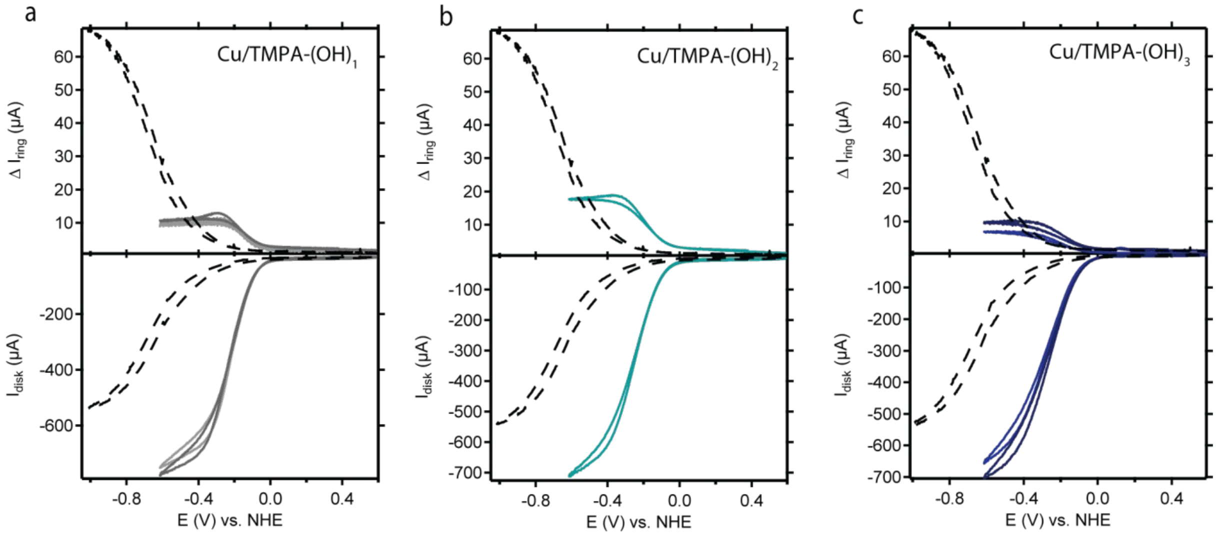

Figure S15 RRDE CVs recorded of Cu/TMPA-$(OH)_1$ (**a**), Cu/TMPA-$(OH)_2$ (**b**), and Cu/TMPA-$(OH)_3$ (**c**) in PB compared to the ORR activity of the bare GC electrode in absence of catalyst (black dashed line). Conditions: p = 1 atm O_2, 0.1 M PB pH 7, 0.3 mM catalyst concentration, ω = 1600 rpm, Pt ring @ 1.2 V vs. RHE, 50 mV/s scan rate.

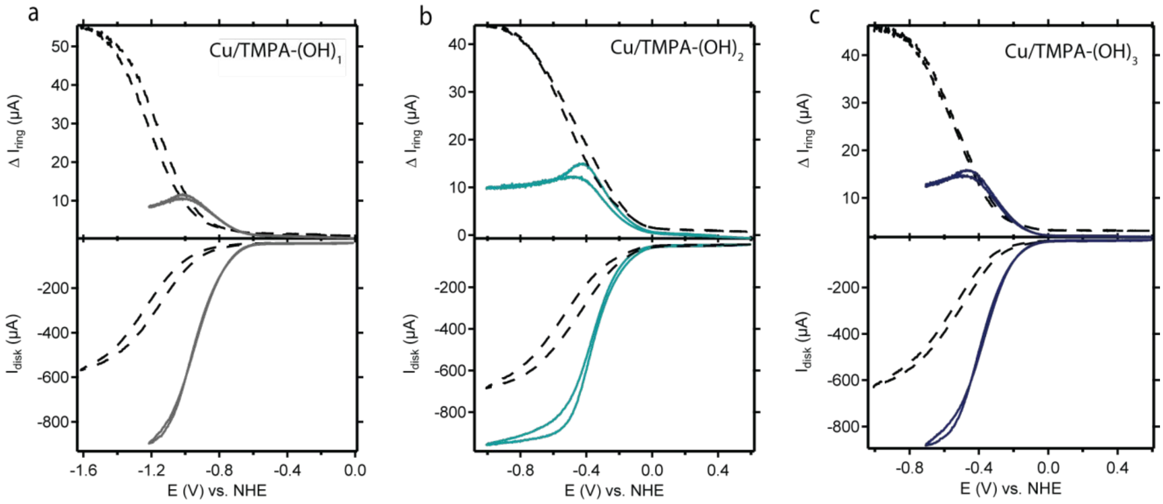

Figure S16 RRDE CVs recorded of Cu/TMPA-$(OH)_1$ (**a**), Cu/TMPA-$(OH)_2$ (**b**), and Cu/TMPA-$(OH)_3$ (**c**) in NaBr compared to the ORR activity of the bare GC electrode in absence of catalyst (black dashed line). Conditions: p = 1 atm O_2, 0.1 M NaBr, 0.3 mM catalyst concentration, ω = 1600 rpm, Pt ring @ 1.2 V vs. RHE, 50 mV/s scan rate.

10. Catalysis in presence of Sodium Pyruvate

RRDE CVs in the presence of SP were recorded for both PB and PB + NaBr (**Figure S17**). From these measurements, it is evident that only a part of the generated H_2O_2 reached the ring in the presence of SP. Therefore, we calculated the transit time of H_2O_2 between the outer edge of the GC disk and inner edge of the Pt ring. This transit time (t') can be calculated from **Equation 5**, which was previously reported.[11]

$$\omega t' = 43.1\left(\frac{v}{D}\right)^{1/3}\left[log\frac{r_2}{r_1}\right]^{2/3} \quad \textbf{(5)}$$

In this equation ω is the rotation speed of the electrode in rpm, *v* is the kinematic viscosity of the electrolyte, which is 0.01 cm^2/s, *D* is the diffusion coefficient of H_2O_2 in water, which is $0.8x10^{-5}$ cm^2/s, r_1 is the radius of the disk, which is 0.5 cm, and r_2 is the inner radius of the Pt ring, which is 0.65 cm. From this equation, it follows that the transit time of H_2O_2 between the disk and ring will be 68 ms.

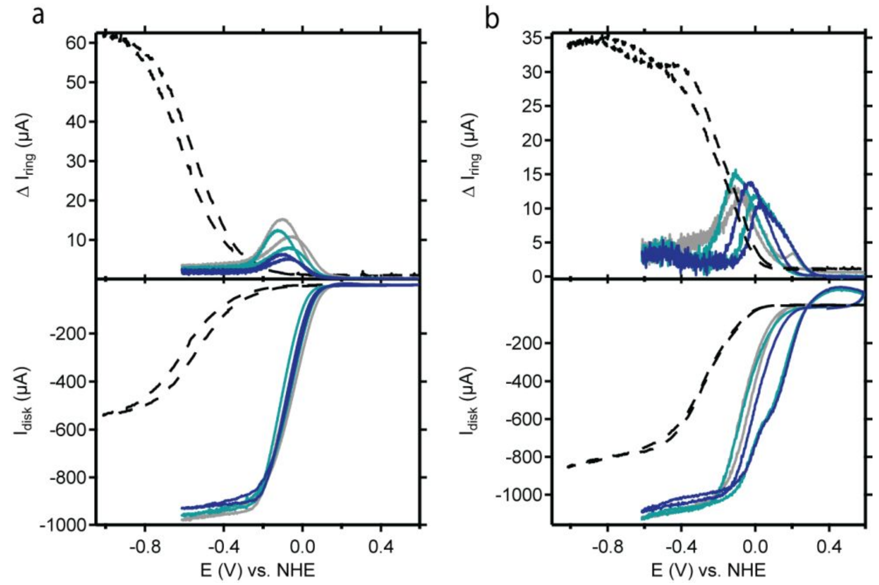

Figure S17 RRDE CV measurements of Cu/TMPA in absence (grey line) of NaPyr and in presence of 50 mM (light blue) and 100 mM (dark blue) NaPyr measured in **a**) PB and **b**) NaBr + 10 mM PB. Conditions: 1 atm O_2, 1600 RPM, Pt ring at 1.2 V vs. RHE, 0.1 M PB pH 7, 0.3 mM Cu(TMPA).

11. Catalysis at Higher Temperatures

RRDE CV measurements of Cu/TMPA were recorded at RT and at elevated temperatures, up to 60 °C (See **Figure S18**). From these measurements, it is apparent that an additional oxidation peak emerged at the ring. This additional oxidation peak likely originates from the oxidation of metallic copper that is formed during catalysis, indicating the decreased stability of Cu/TMPA at elevated temperatures. Increasing *T* also resulted in steepening of the catalytic wave, indicating faster catalysis. Such behavior is expected, as many catalytic processes and O_2 diffusion are known to accelerate with increasing temperature. The increased plateau current at higher temperatures is counterintuitive given the decreased solubility of O_2 at higher temperatures and must therefore arise from an increase in the overall number of electrons transferred due to an acceleration of the HPRR rate.

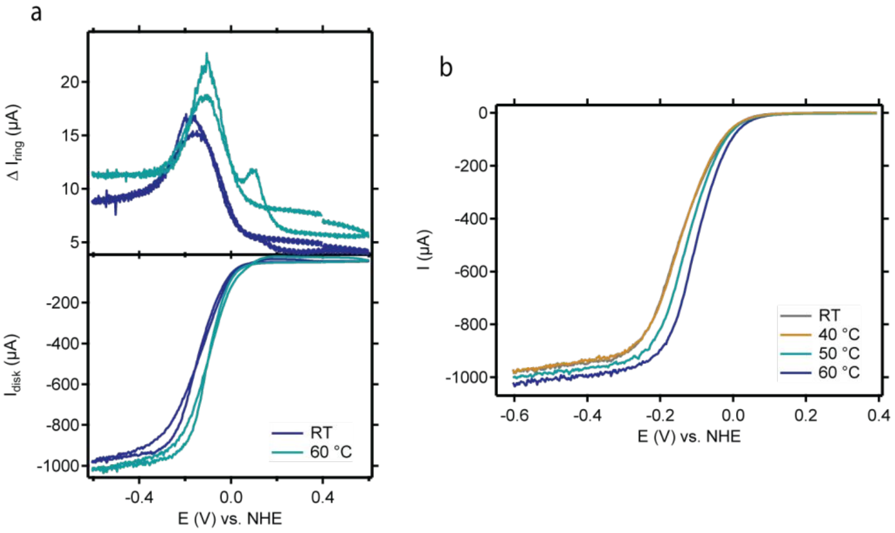

Figure S18 a) RRDE or **b**) RDE CV measurements of Cu/TMPA measured at RT (grey line), 40 °C (yellow line), 50 °C (light blue line), and 60 °C (dark blue line). Conditions: 1 atm O_2, 1600 RPM, Pt ring at 1.2 V vs. RHE, 0.1 M PB pH 7, 0.3 mM Cu/TMPA, 50 mV/s scan rate.

12. Electrochemical Data Recorded against SCE before Polymerization

The potentials were first determined against SCE (**Table S5**), after which they were recalculated to the NHE. The interconversion factor is $E_{NHE} = E_{SCE} + 0.241$ V when $C_{Cl} = 4$ M[12] at 25 °C. At 35 °C, the solubility of KCl changes slightly, so that $E_{NHE} = E_{SCE} + 0.233$ V at this temperature.

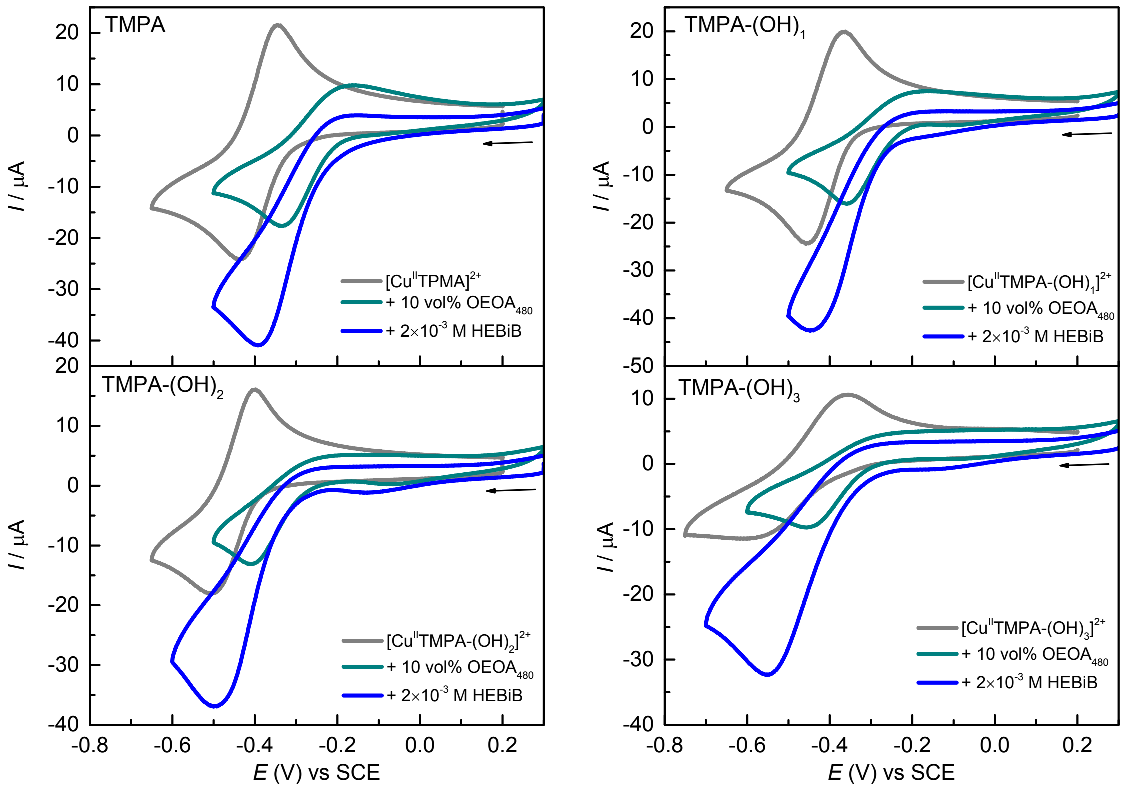

Figure S26. Cyclic voltammetry measurements of 1 mM $[Cu^{II}L]^{2+}$ complexes were recorded before the polymerization of 10 vol% $OEOA_{480}$ in H_2O at $T = 35$ °C, in the presence of 0.1 M sodium pyruvate, 4 mM NaBr and 0.01 M PB. All CVs were recorded on a GC disk electrode at a scan rate of 200 mV/s with SCE as the reference electrode. Arrow indicates the scan direction.

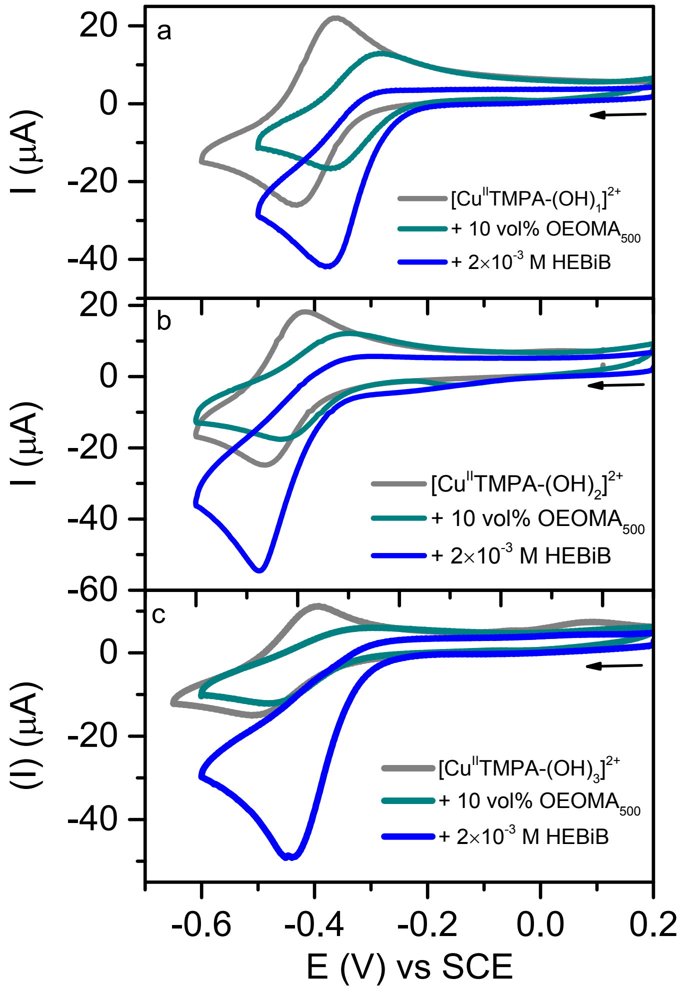

Figure S27. Cyclic voltammetry measurements of 1 mM $[Cu^{II}L]^{2+}$ complexes recorded before the polymerizations of 10 vol% $OEOMA_{500}$ in H_2O + 0.01 M PB + 0.1 M NaCl + 0.1 M SP, at *T* = 35 °C. All the CVs were recorded on a GC disk electrode at 0.2 V/s scan rate. Arrow indicates the scan direction. a) $[Cu^{II}TPMA\text{-}(OH)_1]^{2+}$, b) $[Cu^{II}TPMA\text{-}(OH)_2]^{2+}$ and c) $[Cu^{II}TPMA\text{-}(OH)_3]^{2+}$.

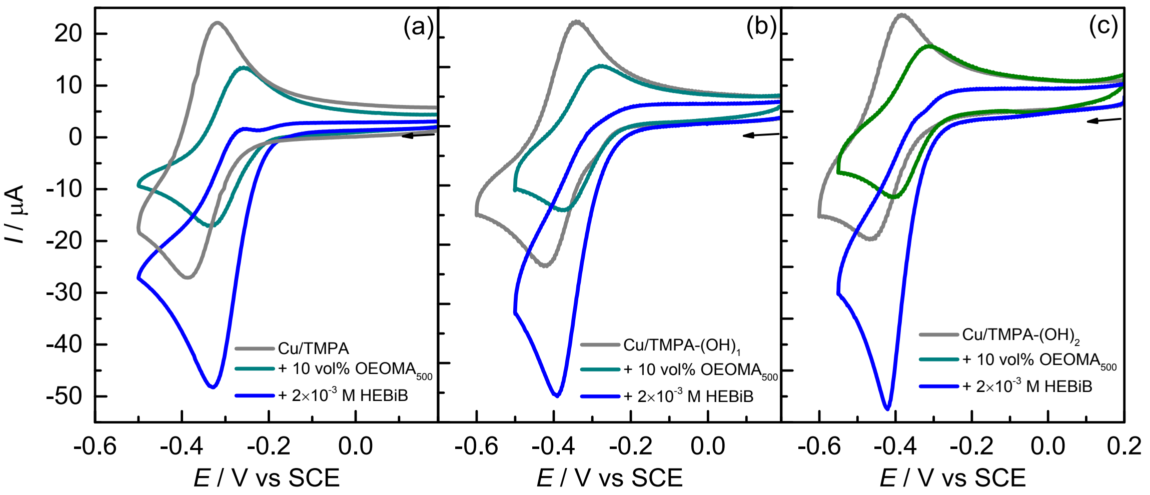

Figure S28. Cyclic voltammetry measurements of 1 mM $[Cu^{II}L]^{2+}$ complexes recorded before the polymerizations of 10 vol% $OEOMA_{500}$ in H_2O + 0.1 M NaBr + 0.01 M PB + 0.1 M SP, at T = 35 °C. All the CVs were recorded on a GC disk electrode at a scan rate of 200 mV/s. Arrow indicates the scan direction. a) $[Cu^{II}TPMA]^{2+}$, b) $[Cu^{II}TPMA\text{-}(OH)_1]^{2+}$ and c) $[Cu^{II}TPMA\text{-}(OH)_2]^{2+}$.

13. *se*ATRP of $OEOMA_{500}$ in the presence of NaCl

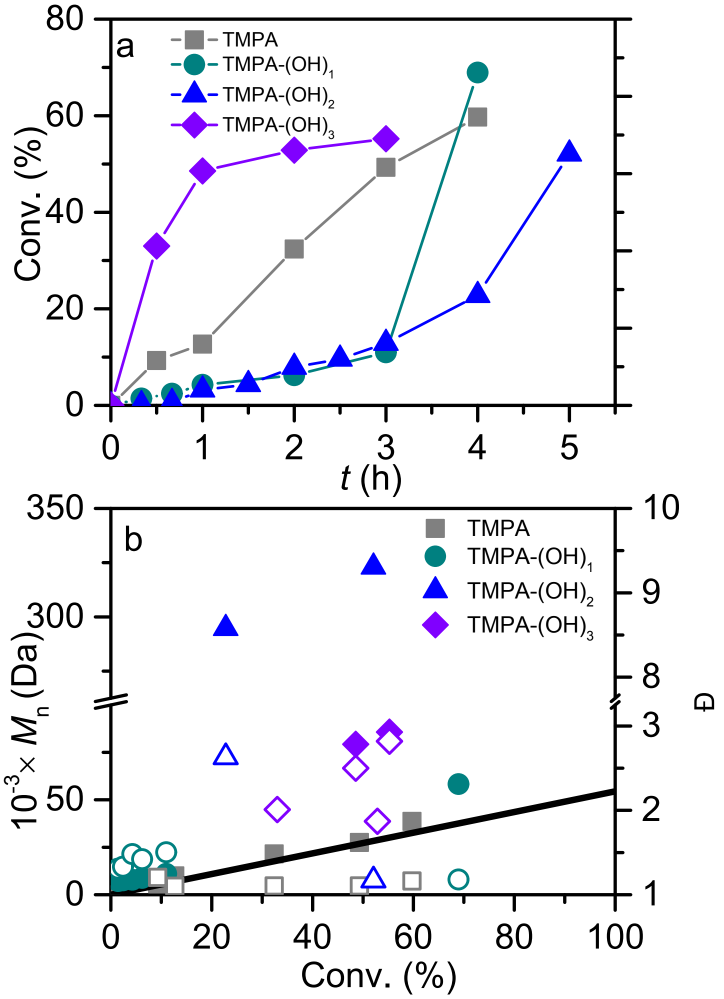

Figure S29. (a) Kinetic plot and evolution of conversion of $OEOMA_{500}$ *vs* *t*; (b) evolution of M_n^{app} and *Đ* against conversion for the *se*ATRP of 10 vol% $OEOMA_{500}$ in H_2O + 0.1 M NaCl at *T* = 35 °C with different catalysts. The straight black line in (b) indicates the theoretical molecular weight. Cu/TMPA (■), Cu/TMPA-$(OH)_1$ (●), Cu/TMPA-$(OH)_2$ (▲), and Cu/TMPA-$(OH)_3$ (♦).

14. Diffusional Limitations during electrochemical ORR-ATRP

Assuming that $[Cu^{I}TMPA]^{+}$ and $[Cu^{II}TMPA]^{2+}$ have the same diffusion coefficient, a diffusion-controlled rate constant of k_d ~ 8 × 10^{10} $M^{-1}s^{-1}$ can be estimated in the general regime of the ORR rate constant according to the following equation[13]:

$$k_d = 4\pi N_A\left(r_{Cu-tmpa} + r_{O_2}\right)\left(D_{Cu-tmpa} + D_{O_2}\right) = \sim 8 \times 10^{10} M^{-1}s^{-1}.$$

The diffusion coefficients of Cu/TMPA and O_2 in water are 4.9 × 10^{-6} cm^2s^{-1} and 1.96 × 10^{-5} cm^2s^{-1}, respectively.[14] The radii of O_2 and Cu/TMPA are 2.0 Å and approximately 2.44 Å, respectively.[15]

15. Supporting References

(1) Beni, A.; Dei, A.; Laschi, S.; Rizzitano, M.; Sorace, L. Tuning the charge distribution and photoswitchable properties of cobalt-dioxolene complexes by using molecular techniques. *Chemistry* **2008**, *14* (6), 1804-1813. DOI: 10.1002/chem.200701163.
(2) Ren, L.; Zhang, J.; Hardy, C. G.; Doxie, D.; Fleming, B.; Tang, C. Preparation of Cobaltocenium-Labeled Polymers by Atom Transfer Radical Polymerization. *Macromolecules* **2012**, *45* (5), 2267-2275. DOI: 10.1021/ma202725c.
(3) Langerman, M.; Hetterscheid, D. G. H. Fast Oxygen Reduction Catalyzed by a Copper(II) Tris(2-pyridylmethyl)amine Complex through a Stepwise Mechanism. *Angew. Chem. Int. Ed.* **2019**, *58* (37), 12974-12978. DOI: 10.1002/anie.201904075.
(4) De Bon, F.; Fantin, M.; Pereira, V. A.; Lourenco Bernardino, T. J.; Serra, A. C.; Matyjaszewski, K.; Coelho, J. F. J. Electrochemically Mediated Atom Transfer Radical Polymerization Driven by Alternating Current. *Angew. Chem. Int. Ed. Engl.* **2024**, *63* (29), e202406484. DOI: 10.1002/anie.202406484. Enciso, A. E.; Lorandi, F.; Mehmood, A.; Fantin, M.; Szczepaniak, G.; Janesko, B. G.; Matyjaszewski, K. p-Substituted Tris(2-pyridylmethyl)amines as Ligands for Highly Active ATRP Catalysts: Facile Synthesis and Characterization. *Angew. Chem. Int. Ed. Engl.* **2020**, *59* (35), 14910-14920. DOI: 10.1002/anie.202004724.
(5) Higuchi, M.; Hitomi, Y.; Minami, H.; Tanaka, T.; Funabiki, T. Correlation of Spin States and Spin Delocalization with the Dioxygen Reactivity of Catecholatoiron(III) Complexes. *Inorg. Chem.* **2005**, *44* (24), 8810-8821. DOI: 10.1021/ic051173y.
(6) Huber, F.; Kirsch, S. F. Site-Selective Acylations with Tailor-Made Catalysts. *Chemistry – A European Journal* **2016**, *22* (17), 5914-5918. DOI: https://doi.org/10.1002/chem.201600790.
(7) Langerman, M.; Hetterscheid, D. G. H. Fast Oxygen Reduction Catalyzed by a Copper(II) Tris(2-pyridylmethyl)amine Complex through a Stepwise Mechanism. *Angew. Chem. Int. Ed. Engl.* **2019**, *58* (37), 12974-12978. DOI: 10.1002/anie.201904075.
(8) Langerman, M.; Hetterscheid, D. G. H. Mechanistic Study of the Activation and the Electrocatalytic Reduction of Hydrogen Peroxide by Cu-tmpa in Neutral Aqueous Solution. *ChemElectroChem* **2021**, *8* (15), 2783-2791. DOI: 10.1002/celc.202100436.
(9) Rountree, E. S.; McCarthy, B. D.; Eisenhart, T. T.; Dempsey, J. L. Evaluation of Homogeneous Electrocatalysts by Cyclic Voltammetry. *Inorg. Chem.* **2014**, *53* (19), 9983-10002. DOI: 10.1021/ic500658x.
(10) Zhou, R.; Zheng, Y.; Jaroniec, M.; Qiao, S.-Z. Determination of the Electron Transfer Number for the Oxygen Reduction Reaction: From Theory to Experiment. *ACS Catalysis* **2016**, *6* (7), 4720-4728. DOI: 10.1021/acscatal.6b01581.
(11) Stockgen, U.; Heusler, K. E. A mathematical method to eliminate the transfer time from disc to ring at a rotating ring-disc electrode. *Electrochim. Acta* **1999**, *44*, 2765-2770. Bruckenstein, S.; Feldman, G. A. Radial transport times at rotating ring-disk electrodes. Limitations on the detection of electrode intermediates undergoing homogeneous chemical reactions. *J. Electroanal. Chem.* **1965**, *9*, 395-399.
(12) Banus, M. G. A Design for a Saturated Calomel Electrode. *Science* **1941**, *93* (2425), 601-602. DOI: 10.1126/science.93.2425.601-a.
(13) Alberty, R. A.; Hammes, G. G. Application of the Theory of Diffusion-Controlled Reactions to Enzyme Kinetics. *J. Phys. Chem.* **1958**, *62* (2), 154-159. DOI: DOI 10.1021/j150560a005.
(14) Han, P.; Bartels, D. M. Temperature dependence of oxygen diffusion in H_2O and D_2O. *J. Phys. Chem.* **1996**, *100* (13), 5597-5602. DOI: DOI 10.1021/jp952903y.
(15) Lim, B. S.; Holm, R. Molecular Heme− Cyanide− Copper Bridged Assemblies: Linkage Isomerism, Trends in vCN Values, and Relation to the Heme-a 3/CuB Site in Cyanide-Inhibited Heme− Copper Oxidases. *Inorg. Chem.* **1998**, *37* (19), 4898-4908.
